# Identification of chemosensory receptor genes in Manduca sexta and knockdown by RNA interference

**DOI:** 10.1186/1471-2164-13-211

**Published:** 2012-05-30

**Authors:** Natalie Howlett, Katherine L Dauber, Aditi Shukla, Brian Morton, John I Glendinning, Elyssa Brent, Caroline Gleason, Fahmida Islam, Denisse Izquierdo, Sweta Sanghavi, Anika Afroz, Aanam Aslam, Marissa Barbaro, Rebekah Blutstein, Margarita Borovka, Brianna Desire, Ayala Elikhis, Qing Fan, Katherine Hoffman, Amy Huang, Dominique Keefe, Sarah Lopatin, Samara Miller, Priyata Patel, Danielle Rizzini, Alyssa Robinson, Karimah Rokins, Aneta Turlik, Jennifer H Mansfield

**Affiliations:** 1 Department of Biological Sciences Barnard College, Columbia University, 3009 Broadway, New York, NY, 10027, USA

## Abstract

**Background:**

Insects detect environmental chemicals via a large and rapidly evolving family of chemosensory receptor proteins. Although our understanding of the molecular genetic basis for Drosophila chemoreception has increased enormously in the last decade, similar understanding in other insects remains limited. The tobacco hornworm, Manduca sexta, has long been an important model for insect chemosensation, particularly from ecological, behavioral, and physiological standpoints. It is also a major agricultural pest on solanaceous crops. However, little sequence information and lack of genetic tools has prevented molecular genetic analysis in this species. The ability to connect molecular genetic mechanisms, including potential lineage-specific changes in chemosensory genes, to ecologically relevant behaviors and specializations in M. sexta would be greatly beneficial.

**Results:**

Here, we sequenced transcriptomes from adult and larval chemosensory tissues and identified chemosensory genes based on sequence homology. We also used dsRNA feeding as a method to induce RNA interference in larval chemosensory tissues.

**Conclusions:**

We report identification of new chemosensory receptor genes including 17 novel odorant receptors and one novel gustatory receptor. Further, we demonstrate that systemic RNA interference can be used in larval olfactory neurons to reduce expression of chemosensory receptor transcripts. Together, our results further the development of M. sexta as a model for functional analysis of insect chemosensation.

## Background

Animals rely on their olfactory and gustatory systems to detect chemicals in their environments. Chemosensation mediates essential behaviors such as locating food and shelter, avoiding predators, locating mates, and selecting appropriate sites for nesting or laying eggs. The chemosensory organs and the gene families that encode chemosensory proteins are different in vertebrates and insects, but the underlying logic is in some respects quite similar [1,2].

The molecular basis of insect chemosensation is best understood in Drosophila. Responses to gustatory and olfactory cues are mediated by the olfactory (OR) and gustatory (GR) receptors, which together comprise an insect chemoreceptor super-family. Receptor protein complexes (which for ORs include the conserved co-receptor protein Orco) function as ionotropic membrane channels, whose precise mechanism of action is still under investigation [reviewed in [2], and see also [3-5]]. OR and GR receptors are expressed in Olfactory Receptor Neurons (ORNs) and Gustatory Receptor Neurons (GRNs) respectively. Most ORNs express a single conventional OR, and the range of compounds to which they respond depends on the tuning of that receptor. GRNs, in contrast, can express multiple GRs [6]. The overall variety of chemical cues an insect can detect is the result of the diversity of its receptors. There are 120 chemoreceptor genes in the Drosophila melanogaster genome which encode 130 different proteins [6]. Additional gene families, encoding Odorant Binding Proteins (OBPs) and Chemosensory Proteins (CSPs) also contribute to taste and olfaction. OBPs are small, water-soluble extracellular proteins within the lymphatic cavity of olfactory sensilla that facilitate ligand binding to ORs [7]. Similarly, CSPs may help mediate binding between ligands and receptors, but their exact role is unclear and the CSPs appear to have additional non-chemosensory functions [8].

Although the gene families encoding chemosensory proteins are conserved across all insects, both chemosensory gene families and the neuroanatomy of chemosensory systems evolve rapidly. Correspondingly, insects show an extraordinary diversity of olfactory and gustatory responses [reviewed in [9,10]]. The full complement of chemosensory genes is now known in species from several insect orders. Frequent losses and lineage-specific expansions of chemosensory genes appear to be the rule, exemplified by particularly large OR gene expansions observed in honeybee, jewel wasp and flour beetle genomes [11-13]. However, outside of Drosophila melanogaster, virtually nothing is known about the function of chemosensory genes, and very few studies have linked specific receptors or lineage-specific genetic changes to particular chemical responses, behaviors or ecology [but see [14-16]]. Such characterization will require not only chemosensory gene identification, but also development of methods for functional analysis of these genes in non-model insects.

The tobacco hornworm Manduca sexta (Lepidoptera: Sphingidae) is a major agricultural pest that feeds on solanaceous plants. Manduca sexta has long been an important non-genetic model organism for insect chemosensation, particularly from anatomical, neurophysiological, behavioral and ecological perspectives [17-21]. Recent analyses have identified 48 ORs and 1 GR, as well as multiple OBP and CSP gene sequences from this species [22-24]. Despite this substantial progress, additional receptors remain to be identified, and a method for functional analysis of these genes is lacking.

RNA interference (RNAi) is an invaluable tool for loss-of-function analysis in non-genetic organisms, and will be important to establish in this context. Systemic RNAi has been used before in M. sexta, particularly to target genes expressed in gut and hemolymph [reviewed in [25]], but there have been no reports of RNAi targeting neural tissue in this species. A previous report showed that peripheral chemosensory neurons could be targeted by feeding double-stranded RNA (dsRNA) to larvae of the moth Epiphyas postvittana [26], suggesting that this approach could potentially be effective in M. sexta chemosensory tissue as well.

Here, we have taken significant steps to further the development of M. sexta as a model for functional analysis of insect chemosensation. We used high-throughput cDNA sequencing to characterize the transcriptome of three chemosensory organs (adult antennae and larval antennae and maxilla), and we identified 17 OR genes not previously known for M. sexta as well as a putative trehalose receptor-related GR gene from this organism. Further, we developed a systemic RNA interference (RNAi) method that can be used to knock down expression of a chemoreceptor transcript in larval olfactory neurons. Although this method will need to be optimized for each gene tested, and physiological assays developed, these findings will allow exploration of the functional role of specific chemosensory receptor proteins in M. sexta.

## Results

### Identification and analysis of M. sexta chemoreceptor genes

**Figure 1:**
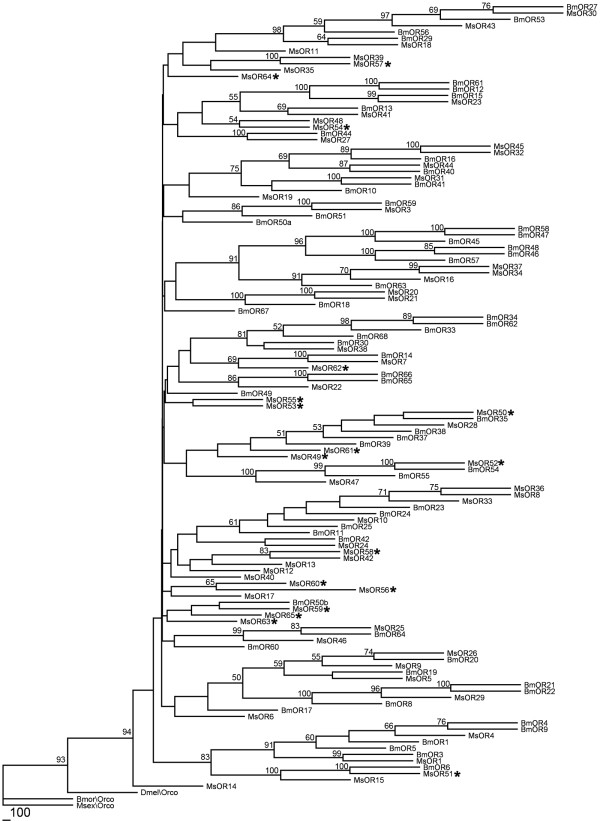
Odorant Receptor genes from M. sexta. Neighbor-joining tree showing similarity of M. sexta and Bombyx OR genes. Bootstrap values from 1000 replicates greater than or equal to 50% are shown. ORs newly identified in this study are marked with an asterisk. The tree was rooted using the Orco co-receptor following previous analyses of this gene family{Robertson, 2006 #111}. Branch lengths are not representative of substitution rate and only vary for presentation purposes.

In order to identify chemosensory genes from Manduca, we performed transcriptome sequencing on mixed chemosensory tissues (adult and larval antenna and larval maxilla). From approximately 52,000 assembled contigs, we identified 84 with significant similarity to insect (Drosophila or Bombyx) ORs. After eliminating those with greater than 95% identity at the nucleotide level to previously identified M. sexta ORs, we identified 17 novel putative OR genes (see Additional File 1: Figure S1). These were numbered randomly starting with MsOR49, building upon the 48 ORs previously identified in M. sexta. A neighbor-joining cluster analysis is shown in Figure 1, which allows inference of potential orthology. Of the 17 putative ORs, 11 show greater sequence similarity to another M. sexta OR than to a Bombyx OR, suggesting that they are the products of recent duplications.`

**Figure 2:**
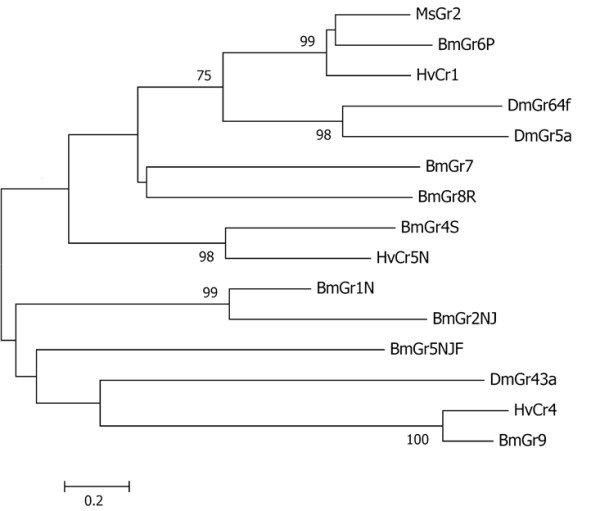
MsGR2 is a putative sugar receptor. Neighbor-joining tree showing similarity of MsGR2 to GR genes from Bombyx, Drosophila and Heliothis. Bootstrap values from 1000 replicates greater than 50% are shown. MsGR2, along with lepidoteran receptors BmGr6p and HvCr1, are most similar to the Drosophila GR family that includes the trehalose receptor DmGr5a [44]and putative sugar co-receptor DmGR64f[45]. Other putative Bombyx sugar receptors (BmGr7, BmG48R, BmGr4S) are more distantly related. This tree was rooted using putative CO_2_ receptors (BmGr1N and BmGr2NJ) and theDmGr43a group, for which ligands are unknown. Sources of sequences shown here are listed in the experimental procedures. Scale bar represents number of amino acid substitutions per site.

By the same criteria, we identified 4 contigs matching other insect GRs, although 3 contigs were redundant leaving 2 putative GR genes from M. sexta (see Additional File 1: Figure S1). One is most closely related to Bombyx Gr6p, both of which show significant similarity to the DmGR5a trehalose (sugar) receptor and the putative sugar co-receptor DmGR64f (Figure 2). The other corresponded to the single bitter GR previously identified [22]. Sequences corresponding to both GR genes were PCR amplified from genomic DNA and cloned to confirm their sequences (data not shown).

Of the contigs with significant similarity to CSP and OBP genes, none were unique to this study (data not shown). This suggests that both gene families are essentially fully known in M. sexta [22].

### Delivery of dsRNA via feeding induces RNA interference in larval antennae

**Figure 3:**
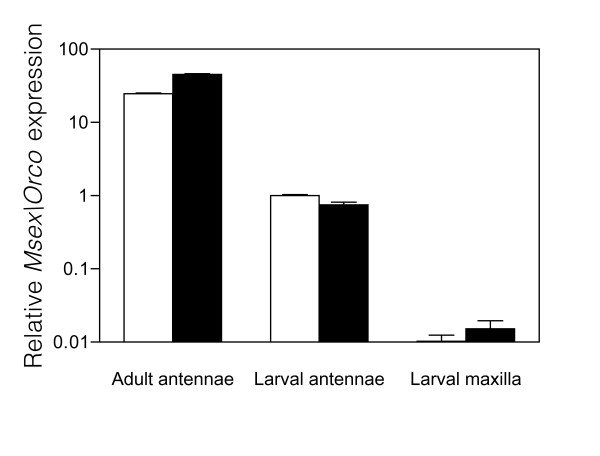
Expression of Msex\Orco in chemosensory tissues. Expression was measured by quantitative RT-PCR (qPCR) in male (white bars) or female (grey bars) adult antennae, 5th instar larval antennae, or 5^th^ instar larval maxilla. For qPCR, relative expression of Msex\Orco was measured and normalized to an endogenous control (rpS3) as described in the Experimental Procedures. Expression was normalized relative to male larval antennal expression and plotted on a log scale. Error bars represent SEM from three analytical replicates on samples that contained tissue from 2–5 individuals (larval samples) or a single individual (adult samples).

We targeted the M. sexta ortholog of the olfactory co-receptor Orco (previously MsOR2) [24,27] for RNAi. In a preliminary analysis, we observed that Msex\Orco transcripts are developmentally regulated and sexually dimorphic. Expression was highest in adult antennae, and was approximately 25-fold lower in larval antennae and 2500-fold lower in larval maxilla (which contain gustatory as well as olfactory sensilla) (Figure 3). As has been previously reported, expression was higher in adult female antennae than in males (Figure 3 and [24]). In contrast, in larval antennae, expression was approximately 1.3-fold higher in males than in females; this difference, though slight, was reproducible across different experiments (Figure 3 and data not shown). To minimize variation in endogenous expression, subsequent RNAi experiments were conducted in staged-matched larvae of the same sex.

dsRNA corresponding to a fragment of the Msex\Orco coding sequence was fed to larvae on the first day of the fifth instar. Transcript levels were monitored by quantitative RT-PCR after 1, 3 or 5 days. For each time-point, three separate samples were prepared from pooled antennal tissue of 2–5 larvae each. Msex\Orco expression was reduced to an average of 80% of expression in paired control samples after 1 day, and to 72% after 3 days. By 5 days, transcript levels were comparable to controls, and even slightly increased (to an average of 110%). None of these differences was statistically significant. However, this was due in part to variability across trials. Although mean Msex\Orco expression 1 day after dsRNA treatment was 80% of controls, in individual trials it was 61%, 72% and 106%.

**Figure 4:**
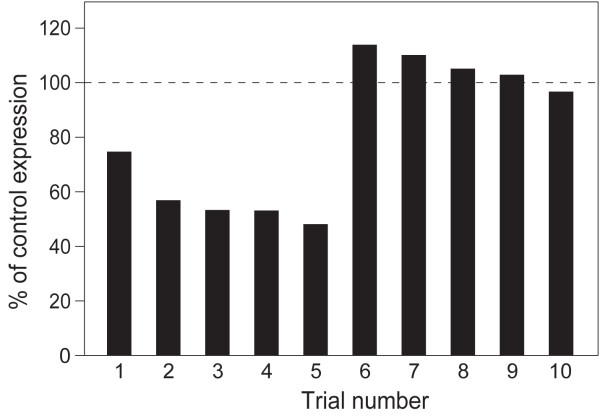
Msex\Orco expression in larval antennae is variably reduced following dsRNA treatment. Relative expression of Msex\Orco was measured by quantitative RT-PCR in male larval antennae 3 days after dsRNA delivery, and here is shown as a percentage of expression compared with paired control samples (dashed line indicates expression in control samples, normalized to 100%). Note that in half of trials, Msex\Orco expression was similar in treated and control animals (trials 6–10) while in the other half, Msex\Orco expression is markedly reduced following dsRNA treatment (trials 1–5). Cumulatively, dsRNA treatment results in significant reduction of Msex\Orco expression (p = .03, paired, one-tailed t-test). For quantitative RT-PCR, relative expression of Msex\Orco was measured and normalized to an endogenous control (rpS3) as described in the Experimental Procedures and shown in Additional file 3: Table S.sx 1

To better characterize this effect, we performed a total of ten trials on Msex\Orco expression 3 days after dsRNA treatment, when transcript reduction was maximal. As described above, each sample contained pooled antennal tissue from 2–5 larvae. In half of the trials, Msex\Orco levels were unaffected (ranging from 97%-114% of expression compared to paired controls; Figure 4 Trials 6–10). In the other half of the trials, transcript levels were reduced following dsRNA treatment, and ranged between 49-75% of control levels (Figure 4; trials 1–5). Despite this variability in success across trials, dsRNA treatment led to a significant overall reduction in Msex\Orco transcript (relative to a null value of 100%), according to a one-sample, one-tailed t-test (t-value = 2.14, df = 9; P ≤ 0.03). Reduction in Msex\Orco transcript levels was specific to Msex\Orco dsRNA, and was not a general effect of dsRNA treatment. When larvae were fed an unrelated dsRNA, corresponding to the olfactory receptor MsOR-30, Msex\Orco expression was not affected, averaging 106% compared to control samples. In the same samples, the targeted MsOR-30 transcript was reduced to an average of 44% compared to untreated controls (not shown; n = 3 trials, 2–3 larvae per sample). Therefore, as discussed further below, feeding of dsRNA can be used to induce RNAi in larval chemosensory tissues.

## Discussion

Manduca sexta has great potential as a model for exploring the functional aspects of chemosensation at the molecular level, due to its agricultural importance and its position as a key model for chemosensory physiology, behavior and ecology. Here, we report identification of OR and GR genes from M. sexta that, along with previously reported genes, means that several dozen chemosensory genes are known in this species. Using the conserved olfactory co-receptor Msex\Orco as a test case, we have shown that systemic RNA interference can be induced in M. sexta larvae, and that it can target peripheral neurons in larval antennae. Although details of RNAi assays will need to be optimized for each gene targeted, together, these results will prove useful for future functional studies of M. sexta chemosensory genes.

The 17 putative ORs that we report here bring the total number of M. sexta OR genes to 64 [22-24]. The M. sexta adult antennal lobe contains approximately 70 glomeruli [22], suggesting that this is a likely approximate number of adult-specific odorant receptor genes, including both ORs and IRs [a second a second class of receptors that we did not investigate in this work; 6 IRs were previously identified in reference [22]]. However, since individual chemoreceptor genes are expressed at low levels, and given known variation in their developmental expression patterns (corresponding to different ecologies of larvae and adults), there are likely additional receptor genes present in the M. sexta genome. In fact, while several ORs identified in our approach were also previously identified (data not shown) it will be interesting to determine whether the 17 ORs unique to our study and reported here are enriched for expression in larvae, because our transcriptome data is derived from both adult and larval tissue, while a previous transcriptome analysis focused on adult antennal tissue [22].

Together, the 64 known M. sexta OR genes provide a good set of targets to explore for future functional analyses. Although potential Bombyx orthologs are clear for many of the genes, many also appear to be the result of recent duplications (Figure 1). Characterizing the function for these receptors could allow us to determine whether particular types of ORs have tended to duplicate recently. The identification of a GR similar to Drosophila sugar receptors also has great potential for functional analysis through RNAi since the physiological and behavioral aspects of sugar response in larval M. sexta is well understood [28].

For RNAi, we targeted the putative ortholog of Drosophila Or83b, Msex\Orco (previously MsOR2) [24,27], which encodes an olfactory co-receptor required for localization and function of conventional ORs [29,30]. Consistent with this role, we detected high levels of Msex\Orco in antennal tissue, which has a large number of ORNs, and much lower expression in the maxilla, which primarily mediate gustation. Expression was higher in adult than larval antennae. In agreement with a previous report, we found that relative expression was higher in adult female than male antennae [24]. In contrast, expression was slightly higher in larval male than female antennae (Figure 3). The functional significance of these differences in expression, if any, is unknown.

We observed significant reduction of Msex\Orco transcripts in larval antennae after feeding dsRNA, indicating that systemic RNAi can be used to target Manduca chemosensory neurons in vivo. Knockdown was statistically significant three days after feeding dsRNA, with successful knockdown observed in about half of the trials conducted. The effect was transient, and lost by 5 days following dsRNA delivery. Together, our results delineate a time-course over which Msex\Orco RNAi can be induced by feeding in larval antennae.

Variation in relative Msex\Orco to rpS3 expression was observed across biological replicates. Among 10 control samples collected at the same stage, expression ratios varied more than three-fold (Additional file 3: Table S 1). The source of this variation is unknown. Importantly, however, we only observed two results in any RNAi trial: expression in paired control vs. treated samples was either approximately equal, or it was markedly lower in dsRNA treated samples. The reverse (markedly higher expression in treated animals) was never observed. This suggests that despite variation in relative Msex\Orco expression, significant differences between dsRNA-treated and control samples are indeed due to RNAi-mediated knockdown.

Variation in relative Msex\Orco expression could account for some of the observed variability RNAi trials. In addition, it is likely that RNAi was not successfully induced in all treated animals. The dsRNA dosage was chosen based on previous reports [in particular, [26,31], and reviewed in [32]], but this should be optimized for each future experiment. Further, it was recently found that starvation improves dsRNA stability in the insect gut and thereby the effectiveness of RNAi [33]. Although animals in our assay were starved overnight prior to dsRNA delivery, there could have been variation in how much (untreated) food they consumed following treatment, as they were offered ad libitum access to the artificial diet. Regardless of its cause, however, variation in knockdown success can be accounted for in future experiments by quantifying target gene expression in each experimental and control animal following phenotypic analysis.

In successful trials, transcript levels were reduced to approximately 50-75% of control levels (Figure 4). These fall within the wide range of 30-98% knockdown reported in previous feeding experiments in other insects [reviewed in [32]], although it is important to note many differences in dsRNA concentration, tissue, and species targeted in these experiments. Interestingly, the mechanism(s) of systemic RNAi are not well understood in insects, and may be somewhat variable. Lepidopteran genomes (and those of other insect orders) do contain a putative ortholog of C. elegans sid-1, which encodes a membrane transporter essential for systemic RNAi in worms [34]. Drosophilids lack a sid-1 ortholog and notably, also lack systemic RNAi in most tissues. However, the role of insect sid-1 family genes is unclear; in Tribolium they are apparently dispensable for systemic RNAi [35]. Further, there are differences in the complement of RNAi effectors in the genomes of Drosophila, Bombyx and Tribolium suggesting that there could be some differences in RNAi mechanisms across insects [35,36].

The degree of RNA knockdown required for detecting a phenotype, and thus for functional analysis of a gene, is expected to vary with the dosage requirement for the targeted gene and with the sensitivity of phenotypic assays. Electrophysiological assays are likely to be able to detect even small differences in taste or olfactory sensitivity, while behavioral assays might require more robust knockdown. While this will have to be established on a case-by-case basis for each gene, partial transcript knockdown in the range of 50% has produced informative phenotypes in several recent cases in insects (reviewed in [32]). Thus, we expect this method will be useful for elucidating the functions of targeted genes.

Manduca has long been a model for studying insect chemosensation, and a wealth of behavioral, physiological and ecological data exists [reviewed in [17,18]]. The ability to pair the identification and functional analysis of chemosensory genes with well-established behavioral, electrophysiological, and molecular methods in this species is likely to significantly advance our understanding of insect chemosensation.

## Conclusions

Chemosensory systems and the gene families that mediate chemosensory responses evolve rapidly. A molecular genetic understanding of insect chemosensation, including an understanding of how evolution of chemosensory genes has contributed to lineage-specific olfactory and gustatory responses, will require both identification and functional analysis of chemosensory genes in non-model insects. In this work, we have identified 18 chemosensory receptors (17 ORs and 1 GR) from the tobacco hornworm Manduca sexta. Further, we demonstrate that systemic RNAi can be used to knock-down expression of chemosensory receptor transcripts in larval olfactory neurons. Together, our results further the development of M. sexta as a model for functional analysis of insect chemosensation.

## Methods

### Rearing conditions and chemosensory tissue collection

All tissues were obtained from male and female Manduca sexta in our rearing facility. The insects were derived from eggs donated by the Manduca rearing facility at the University of Arizona, AZ, USA. Caterpillars were reared on a wheat germ-based artificial diet [37], and maintained in an environmental chamber with a 16 hr light: 8 hr dark cycle at 25°C. Upon pupating, adults were maintained in a holding cage (1 x 1 x 1.5 m) under the same environmental conditions.

Chemosensory tissues were collected from adults (antennae) and 5^th^ instar larvae (antenna and maxilla). Each larval maxilla contained a single lateral styloconic sensillum (taste), medial styloconic sensillum (taste) and maxillary palp (taste and olfaction). The distal tip of the maxillary palp had five uniporous taste sensilla and two multi-porous olfactory sensilla. Tissues were removed under a dissecting scope with iridectomy scissors. For the larval dissections, the caterpillar was anesthetized by immersing its body in a water-filled glass vial, while its head protruded from a latex gasket.

### cDNA library preparation and sequencing

Dissected chemosensory tissues were immersed immediately in Trizol reagent (Invitrogen, 15596–026). Tissue was homogenized with a micro-pestle and stored at −80°C until RNA extraction. Total RNA purification was performed according to manufacturer’s instructions. Construction of two normalized cDNA libraries and 454 pyrosequencing were carried out at the W.M.Keck Center for Comparative and Functional Genomics, Roy J. Carver Biotechnology Center, University of Illinois at Urbana-Champaign. First, messenger RNA was isolated from 20 μg of total RNA using the Oligotex mRNA Mini kit (Qiagen, CA). cDNA was synthesized from 200 ng of mRNA using the SuperScript® Double-Stranded cDNA Synthesis Kit (Invitrogen, CA) with 100 μM random hexamer primers (Fermentas, USA). Double-stranded DNAs were nebulized at 32 psi for one minute, concentrated with a Qiaquick PCR minelute column (Qiagen, CA) and blunt-ended (25 μl water, 10 μl 10x T4 DNA Ligase buffer (NEB), 4 μl 10 mM dNTP mix, 5 μl T4 DNA polymerase (3 U/μl) (NEB), 1 μl Klenow polymerase (5 U/ μl) (NEB), and 5 μl Polynucleotide kinase (10 U/μl) (NEB). A dA-overhang was added at 3’ ends of cDNA by adding the following to the blunt-ended cDNA: 5 μl 10x buffer 2 (NEB), 10 μl 1 mM dATP and 3 μl Klenow exo-minus polymerase (5 U/μl) (NEB). The reaction was incubated at 37°C for 30 minutes and then cleaned up with a QIAquick MinElute column and eluted in 10 μl EB. The cDNA was adaptored with Titanium adaptors (454 Life Sciences, Branford, CT) by adding 9 μl water, 25 μl 2x Rapid Ligase buffer (Enzymatics, MA) 5 μl (50 μM) Titanium adapter A/B mix and 1 μl T4 DNA Ligase (600 U/μl (Enzymatics, MA) and incubated the ligation reaction at room temperature for 15 minutes. The reaction was cleaned up using a Qiaquick MiniElute column (Qiagen), eluting the cDNA in 20 μl EB. Adaptored cDNA was run on a E-GEL EX 2% agarose (Invitrogen, CA) following the manufacturer instructions and cDNAs in the size range of 400-800 bp were excised from the gel and purified with a Qiagen’s Gel Extraction kit and the cDNA was eluted in 30 μl EB. The gel- purified cDNA was used as template for amplification in 50 μl PCR reactions containing 10 μl 5x Phusion Buffer HF (NEB), 25 μM Adapter A_For primer (5’CCATCTCATCCCTGCGTGTCTCCGACTCAGACGAGTGCGT3’), 25 μM Adapter B_For primer (5’CCATCCCCTGTGTGCCTTGGCAGTCTCAGT3’), 3% DMSO, 10 mM dNTPs and 1 U Phusion polymerase (Finnzymes/NEB, USA). The PCR conditions were as follows: 98°C for 30 seconds, followed by 10 cycles with 98°C for 10 seconds, 68°C for 30 seconds and 72°C for 30 seconds, with a final extension of 72°C for 5 minute and cleaned up with a Qiaquick minelute PCR column. The cDNA librares were normalized with the Trimmer Direct Kit (Evrogen, Russia). In brief, 300 ng of cDNA were denatured at 95°C for 5 minutes and allowed to reanneal at 68°C for 4 hours in the hybridization buffer included in the kit (50 mM Hepes, pH7.5 and 0.5 M NaCl). After the incubation, the reactions were treated with ¼ units of duplex specific nuclease (DSN). The normalized cDNAs were then PCR amplified as described above.

After library construction, the samples were quantified using a Qubit fluorometer (Invitrogen, CA) and average fragment sizes were determined by analyzing 1 μl of the samples on a Bioanalyzer (Agilent, CA) using a DNA 7500 chip. The libraries were mixed in equimolar concentration and diluted to 1x10^6^molecules/μl. Emulsion-based clonal amplification and sequencing on the 454 Genome Sequencer FLX Titanium system were performed according to the manufacturer’s instructions (454 Life Sciences, Branford, CT). Signal processing and base calling were performed using the bundled 454 Data Analysis Software version 2.0.01.

### Contig identification

Primary cDNA sequences were assembled into approximately 52,000 contigs. A standalone tblastx was run using default parameters for each of the OR, CSP, and OBP gene families, and a standalone tblastn was run using default parameters for GR gene families. For the ORs and GRs, all available sequences in the gene family from both Bombyx mori and Drosophila melanogaster were used as queries. In the other two cases, all protein sequences from Bombyx mori were used (see Additional file 2: Figure S 2). All query sequences were downloaded from NCBI except for the Bombyx OBP sequences, which were obtained from the authors of [7]. All contigs with matches to at least one query sequence with an E value less than e^-45^ were retained and for each of these contigs the sequence within the range given in the Blast hit was used for further analyses. In some cases the Blast result gave multiple significant results for a contig in different reading frames along the same strand. When these sequences were contiguous in both the query and in the contig, indicating that it was most likely the result of an indel sequencing error, the two protein sequences corresponding to the two reading frames in the contig were joined contiguously.

### Phylogenetic analysis

For each gene family, amino acid query sequences and translated contig sequences were aligned using ClustalW under default parameters [38]. Phylogenetic analyses on these alignments were performed using Phylip 3.69 (Felsenstein, 2010). A Neighbor-Joining cluster was generated for each alignment using the JTT model without rate variation across sites. Bootstrapping was performed by generating 1000 resampled alignments with the SeqBoot program of the Phylip package and clustering each of them in the same manner as the original alignment. The 1000 clusters were then used to generate a majority rule consensus tree.

For phylogenetic analysis of gustatory receptors, predicted amino acid sequences were aligned using ClustalW with default parameters in Mega 5.04. A neighbor-joining cluster was generated, with bootstrap values calculated by resampling 1000 times, and a consensus tree generated, all using default parameters in Mega 5.04. The following sequences were used: HvCr1, CAD31850.1; DmGr64f, NP_728924.2; DmGr5a, BAB68248.1; DmGr43a, NP_001036531.1; HvCr4, CAD31946.1; HvCr5n, AJ487480.1; BmGr9, EU769120.1; BmGr8R, NM_001130872; BmGr7, BK006594. Remaining Bombyx sequences are from [39].

### Preparation of dsRNA

A 957 bp fragment of the Msex\Orco open reading frame was amplified from genomic DNA using the following primers 1893A5a, 5’- TATAACCATGCAATAACAAA-3’; 1893A3a 5’-AGTAACCTGGGAAAAATAAT-3’ and subcloned into the pDrive vector (Qiagen, cat #231122) according to manufacturers instructions. dsRNA was prepared as recommended by the Drosophila RNAi Screening Center (flyrnai.org and [40]. Briefly, the subcloned fragment was PCR amplified to append T7 sites to each end (primers: 5’-TAATACGACTCACTATAGGGTATAACCATGCAATAACAAA-3’ and 5’-TAATACGACTCACTATAGGGAGTAACCTGGGAAAAATAAT-3’). Both strands of the resulting product were in vitro transcribed with the Ambion MEGAscript kit using T7 RNA polymerase according to manufacturer’s instructions (Ambion, cat #AM1333). RNA was purified by LiCl/ethanol precipitation and redissolved in depc-dH_2_O. RNA strands were annealed by incubating in a boiling water bath and immediately allowing to slow-cool to <30°C, followed by dilution in depc-dH_2_O to 1 μg/μL. dsRNA was used immediately or aliquoted and stored at −80°C until use. dsRNA corresponding to a 208 bp fragment of MsOR-30 was generated following the same procedure. Template DNA was amplified from genomic DNA using the following primers Fwd: 5’-TTCGCAGTTCAAGAAGAGCA-3’ and Rev: 5’-TTCGTGCATATATTTTTGAAAGTGA-3’.

### dsRNA delivery

Individual larvae were collected on the last day of the fourth larval instar, placed in separate containers, and starved overnight. On the first day of the fifth larval instar, dsRNA was delivered by feeding starved caterpillars a small pellet of food that had been injected with 3 μg Msex\Orco dsRNA (1 μg/μL in depc-dH_2_0) or with 3μL depc-dH_2_0. After 5–6 hours, larvae that had consumed the entire food pellet were kept for subsequent analysis; they were returned to an unrestricted diet and housed individually until tissue collection. For paired samples (dsRNA treated and control), larvae were collected either on the same day or within a few days of one another, with the exception of 2 trials, in which samples were collected 3 or 5 weeks apart. For Msex\Orco dsRNA trials, all larvae were males; for MsOR-30 trials, all larvae were females. RNA extraction, cDNA synthesis and qPCR was carried out in parallel for each paired sample.

### Quantification of gene expression

Chemosensory tissues were harvested at 24, 72 or 120 hours after dsRNA delivery for quantification of Msex\Orco expression. Larval antennae (antennae preps) or maxilla (maxilla preps) were dissected and combined from 2–5 individuals of the same sex into a single tube. The sex of each larva was determined as previously described [41]. Dissected tissues were immersed immediately in Trizol reagent (Invitrogen, 15596–026) and tissue was homogenized with a micro-pestle and stored at −80°C until RNA extraction. Total RNA purification was performed according to manufacturer’s instructions. First strand cDNA synthesis was performed using random hexamer primers and the AMV First Strand cDNA synthesis kit (Invitrogen, 12328–032) according to manufacturer’s instructions.

Real-time quantitative RT-PCR was performed using the SYBR Green method with a Lightcycler 480 instrument (Roche Applied Sciences). Primers were designed with Primer3 [42] to amplify both MsOR2 and the 16 S ribosomal protein transcript rpS3 [43], which was used as an endogenous control for relative quantification of expression. The primer sequences were as follows: Msex\Orco-Fwd1: 5’-GAACACTTGTCCGAGGGTGT-3’, Msex\Orco-Rev1: 5’-ACTGGGTTGAACGCCATAAG-3’; rpS3-Fwd2: 5’-GCAGAAGCGGTTCAACATC-3’, rpS3-Rev2: 5’- AGACCTCCAATGAGTTTGTATC-3’; MsOR-30 Fwd: 5’- CAAAGGAACACGAAAGACGA,-3’, MsOR-30 Rev: 5’- CGACCACAATAACCACCGTA-3’. The specificity of each pair was confirmed by melt-curve analysis and efficiency was calculated over a 50-fold cDNA dilution range (2.050 for Msex\Orco, 1.977 for rpS3 and 1.965 for MsOR-30 primers). Experimental reaction conditions were as follows: 20μL reactions were prepared with SYBR Green master I mix (Roche, 04 707 516 001), 20pmol each primer, and 0.5μL cDNA template, no-RT control template, or dH_2_0 negative control. Reactions were carried out for a varied number of cycles (5 min 95°C incubation followed by cycles of 10 sec at 95°C, 10 sec at 57°C, 16 sec at 72°C until experimental reactions had gone to completion). Each reaction was performed in triplicate. The fit-points method was used to calculate crossing points, and expression of Msex\Orco normalized to rpS3 using the Roche Lightcycler 480 software version 1.5.

## Misc

Natalie Howlett and Katherine L Dauber contributed equally to this work.

## Competing interests

The authors declare that they have no competing interests.

## Authors’ contributions

This study was conceived of by BM, JIG and JHM, and experimental design was performed by BM, JHM, JIG, NH and KLD. Gene identification was performed by NH and BM, with contributions from KLD, EB, CG, FI, DI, and SS. Verification of selected genes by PCR was performed by NH, KLD and AS, with contributions from DI, AA, MB, RB, MB, BD, AE, QF, KH, AH, DK, SL, SM, PP, DR, AR, KR and AT. RNAi analysis was performed by KLD, AS and JHM. The manuscript was written by JHM and BM. All authors read and approved the final manuscript.

## Supplementary Material

Additional file 1 **Figure S1.** Predicted amino acid sequences of M. sexta OR and GR genes newly identified in this study. Genbank accession numbers are given at the beginning of each file name. Click here for file

Additional file 3 **Figure S3.** Table S1. Relative expression of Msex\Orco/RPS3 measured 3 days after dsRNA treatment. Quantitative PCR data from 10 paired trials are shown; these data are graphed in Figure 4. The SEM was calculated from triplicate measurements of each sample. Click here for file

Additional file 2 **Figure S2.** Amino acid sequences used as BLAST queries to identify putative M. sexta OR, GR, OBP and CSP sequences. Click here for file
