# Supplementary material for: Identification of chemosensory receptor genes in Manduca sexta and knockdown by RNA interference
Source: BMC Genomics. 2012 May 30;13:211. doi: 10.1186/1471-2164-13-211 (PMC3464597; doi:10.1186/1471-2164-13-211)
Supplement: Additional file 1 — Figure S1. Predicted amino acid sequences of M. sexta OR and GR genes newly identified in this study. Genbank accession numbers are given at the beginning of each file name. [file 1471-2164-13-211-S1.pdf]

Figure S1: Predicted amino acid sequences of *M. sexta OR* and *GR* genes newly identified in this study. Genbank accession numbers are given at the beginning of each file name.

>JQ794807 MsOR49 [organism=Manduca sexta] Manduca sexta odorant receptor 49, partial
GLEPTIETPNYENFVFIKVVAVSFVCFSGIIIILVGYVEANMLSLSVEIKEIWNDAV
ARDKKEKKEYQHLKNQPMKIVNMYVKRRLVNIIERHKANINLLSDVEHLFRIPIG
AGFLCQTISIVAELLGGLKNTYLEVPFGLVQITMDCYIGQKVMDASLIFEEAVYD
CKWEKFDNSNMKLVLTILQNSQKTMRLSAGGMATMSFSCLASVLNSIYSTYTTL
HAKIN

>JQ794808 MsOR50 [organism=Manduca sexta] Manduca sexta odorant receptor 50, partial
MISIWTGIRKFGLEYCDLPTMLWNVAFLLRPLTLNIDSRHKKPIPLLFYVLTVVITS
SYFYVYLISMSWFVFVRSLETKELIPAMVVLSLGISSEIGTLKFFYTFVYIDKVRKI
VDEYLECDALVVPFSRFSKNLLTTLRFVKKRAIIYWLVIIGNGFAYWSKPLFMKG
RHHLEDNLVIYGLEPMLESPKYEIAYFLMTAGVCFICYPPANVTILLIVVVGYTEA
QMLALSEEILNIWDDANDHYNNLPSTNQNTNELKSKIINKYIKDRLKDITKSHAR
NINLLRQVEFVFRGAIAIGYVFLILGLIAELLGGLENTFLQIPFAFIQVAIDCFTGQR
VMDANIVFETAVYDCKWENFDKTNMKTVLLLLQNSQKTLTLSAGGXTMLNFKC
XMSVIKSIYSAYTTLRT

>JQ794809 MsOR51 [organism=Manduca sexta] Manduca sexta odorant receptor 51
MGQDYYLQPPRTQLFYTILGHISTIFGTCQMKWWGPEYKISKWLAWLYFLQRLS
VGTFGKVVCLSQSVFMALNFNTLSSSVLIVILTITPVGYLVAVKAETAKLKSYEK
VMKAFMDKIHIYNCYVRNKDSEYVKTKVKQVEFLSRFTTWFLTFFLYICWSAW
MLQPTLYNIRNIDAILNKTQDFQYYIYLWTPLEYHHNLRNYLIIHTVCVYLGVTAI
TVIITFDCLNMIIVYHIVGHIYILKHNFRTKLADDFTDEKAKEFLVDTIKYHSFIITT
FKDVQNAFGINVAANYLQKFIEDGLCLYQMMKGDMENFVKYGLMLVIYLGSLI
MLSVVLEEIKRQNSDLSEVVYSIPWQRMSVSNQKTVMLLLFRTQPDLEFRAACG
MKAGVQPALSIIKSMFSYYVMIKSR

>JQ794810 MsOR52 [organism=Manduca sexta] Manduca sexta odorant receptor 52
MLSKLKARLNNINERFRDVSFDSLMWIVNIAPNLVGFSVRGDKAGAAFWFIHLF
LLTYVYGVGSVVYQWKFAHTIGDYIKSYINVSLVIFIANNSWWFISKRLQVKDVL
EKIALSDQLACQTDASQKKYKKMLKIIKHIIIGFYAVNYIDELFIYLPHRVDVRND
YSLTPCVGLEPLTQSPNKEICSFILCLQECTIATAVLNYQALLLLLIAHTAAMYRIL
SAEMMSFNDYDNLEEHQARAKKILPSLVERHVLILCVIDKLKSLYSVSLGVNFGS
HAVCLLLLLYLPIRESFMFIPIFAYCFSVFFLYCFLCQKLVNASEDFERSVYCCGW
ENVLKERRMVYFMLCEAQRPVEILAADIVPVNIYTFATTLQAMYKFVTVVK

>JQ794811 MsOR53 [organism=Manduca sexta] Manduca sexta odorant receptor 53, partial
GNTPHTKIFLRQIKINMWVSGIPFGDTKIYIRYYILLSLLISMIIAEGSFFVSRISSEN
FLELTQLAPCACIGLLSFLKILPITVKRRKIFDLTERLGRLYENILDDTTKKANCKN

VNCNLFKACSQSIFFVLNAVLIS

>JQ794812 MsOR54 [organism=Manduca sexta] Manduca sexta odorant receptor 54, partial
YEIFQITWYLYKKLPISFLQRLFNNVNIYFSFIFYDLLCTINFFYLPVQLDLIIEELVF
YFMELAAISKVLTFVFFRDKLAKILDALEDPMFQAANGKEQKIIDRAKRFNKRY
WKIVATVSLTSHATHILSPIVEHLFLSVPLQPPTCSYSFLSENTIQQFIYPLYLYQTL
GMHCHLWFNVNTDSFFLGLVILIIAQLQILDLRLRTVTDVKKNDDIGQVSTSEAR
ANYSLTQLNK

>JQ794813 MsOR55 [organism=Manduca sexta] Manduca sexta odorant receptor 55, partial
MGWLERIKGIVLKKSFDFDRPDICLYNFHPQLRILFALKGIFFNKQNSKLRFILPAY
FNILTLLGMVFESMFIYRGLTIKDYSFAIESFLYFIILTSTPLIYSCLFYHKDKIIQLL
DDMNEEFK

>JQ794814 MsOR56 [organism=Manduca sexta] Manduca sexta odorant receptor 56, partial
DLIQVWGDITLMTGTSFLLFTNMAFITKIINVMVRRDAVLAIIDEGDEVLRSERRIE
GKAIVKSSNQETSRLLYLYGLFTVVTVFGWAASAEKGSLPLRAWYPYDTSKSPA
YELTYLHQSVAVILLAFLNVSLDVLVSSLTAV

>JQ794815 MsOR57 [organism=Manduca sexta] Manduca sexta odorant receptor 57, partial
WFPWSLDNFKVYIASFVFHAYGASLCCITNPGFQSTIILLVGQLIRQLRILTFILLNL
NDLVIELVGNKNPRWQAHCTSVLVQCVKHYIKLKRFSNGLNYICRPFYLTLILVS
TMLVCMCSVKIATSEKLTPDTIKYYVHEFCFILIVLMFCLLGQQVENECRELEVT
ALEKWYIFNKQHQSNVRIFHIAVSQRMPIYIFGTIPLSLPTFTWFMKT

>JQ794816 MsOR58 [organism=Manduca sexta] Manduca sexta odorant receptor 58, partial
SLWRKYFTKEIQVLKRVYERSDYEDTYEIPRKYLKWSGIRMKHIISKPVSICWLV
YYWFYFANIVFAFTSELMGTCMTASANTFTDAIAFFQMVPCIGYCGMSLVKSFK
MVKHRPVFENLITEIGDMWPQRQVDEEEHKIISSALREIKNVVKGYQWGNNCLIL
SFLYAPFWELFKRFSGEKWEPKLQFIYWLPFDPSQPVYYECTLVLQTWQAITVIW
TNMTSDIMFCLFLSHISTQFNLLSVKIEKLIYVPTDQQLIEFYPLGQYSEEYLRKNK
EAVDSYTPQQWEEKNFKEITEIVLQHQALIRLSEDIENMFSLTLLVNVINSSLLICL
CGFCSVVLEKWNETA

>JQ794817 MsOR59 [organism=Manduca sexta] Manduca sexta odorant receptor 59, partial
KMMAKVKTQGLVTDVMPNIKLLQLSGHFLFNYYADNSGMTMLLRKMYSTVHA
ILIFVQFVCMGVNMAMYADEVNELTANTITVLFFAHSIIKLGFLAFTSKSFYRTM
AVWNQSNSHPLFTESDARYHQIALTKMRR

>JQ794818 MsOR60 [organism=Manduca sexta] Manduca sexta odorant receptor 60,

partial
ITLRLKQYIDYHREVLEFTQDISEAFGPMLFVYYLFHQVSGCLLLLECSQMSDAA
ALMRYGLLTAVLFQQLIQLSVVVESVGTVTGYLKDAVYNVPWEYMD
TQDRKTVCIFLMNVQEPVHINALGLAKVGVQAMD

>JQ794819 MsOR61 [organism=Manduca sexta] Manduca sexta odorant receptor 61, partial
KIFQGLEPMLESPKYEIAYFLMTAGVCFICYPPANVTILLIVVVGYTEAQMLALSE
EILNIWDDANDHYNNLPSTNQNTNELKSKIINSILKDRLKDITKSHARNINLLRQV
EFVFRGAIAIGYVFLILGLIAELLGGLENTFLQIPFAFIQVAIDCFTG

>JQ794820 MsOR62 [organism=Manduca sexta] Manduca sexta odorant receptor 62, partial
RNTKMEIKTDKPREKKFKTFNETFSLCAFALAFAFLYPNRTNAVKRAVIITLIILFN
GGQLFWFITYTLKCLYTLDILNFARNMTLAVVLILFFIKTYYVIYATKDFAPILIK
MSNDLLAANELEEDYQAIYEEHIRQGKVGQISWLLIPIVLSAQFPIYAGICMSIESI
KTDNFTRLMVHDMELLFVEDIQSETPFFQCMFAYNCCQCIVLVPNYCGFDGSFCI
ATTHLRMKLKLMTHKVHRAFAHAQNTNELRKMVNEAIQDHQDALKFYKDMQ
HVYGPWLFAVFMLTSFMISFNLYMIYLLKRVDPKYTLFGLVGVLHIYLPCHYAS
TLTKVGEEIGPDLYDTPWEKWADPEVTKLLIFMIARAQKTLIVTGNGLVVFNME
LFKSIIQSSYSFFTLIT

>JQ794821 MsOR63 [organism=Manduca sexta] Manduca sexta odorant receptor 63, partial
HDLVAAMVILSLGVCSEVSVIKLGFMAFNSDEVRSIVDHFLECDASVLPGTRYYN
NIMKTLRIVKRRAVGYWVTYRDERGLFITSPPYLGLGRHLSEDLMVIYGLEPTIET
PNYE

>JQ794822 MsOR64 [organism=Manduca sexta] Manduca sexta odorant receptor 64, partial
PKPKRNREILMNNIYLGMVLFVLTYMPVALAVHLYTEWQDIMSSLDKIADCLPL
LVSVAIVSYYALYRNDLYTLVDYMEKNFKHHSARGLTNMTMWGSYCKARNFG
RIYTACTMFSVTMYVTLPLILHLWTKEPIQSWVYSDVTQSPFLEFVFLRSFLTQFF
VGL

>JQ794823 MsOR65 [organism=Manduca sexta] Manduca sexta odorant receptor 65, partial
MALKYPWNKSKEWFXRIVFWAYLSGLPNFWIEDLNFSKHFMKFYDKYARALDII
SCIFVLLELLSVFTQHELSKKQQTIQILFCVGHPFLCLYSALMGHYKEKTRGVLLE
LVVTLKQVHNDPNVEKMMIKQCNMYSIAFTFSCFCSMLFYCVDSLIQVLKTGVT
FNVVIPVLADHR

>JQ794824 MsGR2 [organism=Manduca sexta] Manduca sexta gustatory receptor 2, partial
FYCHFTTLLVIKYGTTEHTKEFWRFWVPVKRNKVHPANFKKQTPATFQK
SLRATLLIGQAFFTFYLWSEYFSNDANNVKFIITSWKCFYSLLSFFGQIF

IVVMCIIRVVSTETTLNATTPIIFYGTTCFTMLMFFRVATAWPDLVQHVA
KTEELYPNYDNKLTRTCQ
