# Supplementary material for: Identification of chemosensory receptor genes in Manduca sexta and knockdown by RNA interference
Source: BMC Genomics. 2012 May 30;13:211. doi: 10.1186/1471-2164-13-211 (PMC3464597; doi:10.1186/1471-2164-13-211)
Supplement: Additional file 3 — Figure S3. Table S1. Relative expression of Msex\Orco/RPS3 measured 3 days after dsRNA treatment. Quantitative PCR data from 10 paired trials are shown; these data are graphed in Figure 4. The SEM was calculated from triplicate measurements of each sample. [file 1471-2164-13-211-S3.pdf]

Table S1: **Relative expression of *Msex*\Orco/*RPS3* measured 3 days after dsRNA treatment.** Quantitative PCR data from 10 paired trials are shown; these data are graphed in **Figure 4**. The SEM was calculated from triplicate measurements of each sample.

Trial	Control ratio *Msex*\Orco /*RPS3*	Control SEM	dsRNA-treated ratio *Msex*\Orco /*RPS3*	dsRNA-treated SEM
1	1.356E-01	2.410E-03	1.019E-01	2.640E-02
2	2.279E-01	1.990E-02	1.303E-01	3.250E-03
3	1.412E-01	1.210E-02	7.600E-02	6.130E-03
4	1.671E-01	1.280E-02	8.960E-02	1.650E-02
5	7.230E-02	1.470E-02	3.510E-02	8.580E-03
6	6.990E-02	6.350E-03	7.990E-02	2.180E-02
7	1.130E-01	8.860E-03	1.247E-01	9.820E-03
8	1.239E-01	1.410E-02	1.306E-01	2.210E-02
9	2.427E-01	3.360E-02	2.507E-01	4.040E-02
10	1.722E-01	3.330E-02	1.672E-01	2.140E-02
