# Supplementary material for: Identification of chemosensory receptor genes in Manduca sexta and knockdown by RNA interference
Source: BMC Genomics. 2012 May 30;13:211. doi: 10.1186/1471-2164-13-211 (PMC3464597; doi:10.1186/1471-2164-13-211)
Supplement: Additional file 2 — Figure S2. Amino acid sequences used as BLAST queries to identify putative M. sexta OR, GR, OBP and CSP sequences. [file 1471-2164-13-211-S2.pdf]

Figure S2. Amino acid sequences used as BLAST queries to identify putative *M. sexta OR*, *GR*, *OBP* and *CSP* sequences. OR queries are listed on p. 1-18; GR queries on p. 18-38; OBP queries on p. 39-42; CSP queries on p. 43-44.

I. Predicted amino acid sequences of Odorant Receptor queries

```
>DmOR83bIa
MTTSMQPSKYTGLVADLMPNIRAMKYSGLFMHNFTGGSAFMKKVYSSVHLVFLLMQFTFILVNMALNAEE
VNELSGNTITTLFFTHCITKFIYLAVNQKNFYRTLNIWNQVNTHPLFAESDARYHSIALAKMRKLFFLVM
LTTVASATAWTTITFFGDSVKMVVDHETNSSIPVEIPRLPIKSFYPWNASHGMFYMISFAFQIYYVLFSM
IHSNLCDVMFCSWLIFACEQLQHLKGIMKPLMELSASLDTYRPNSAALFRSLSANSKSELIHNEEKDPGT
DMDMSGIYSSKADWGAQFRAPSTLQSFGGNGGGGNGLVNGANPNGLTKKQEMMVRSAIKYWVERHKHVVR
LVAAIGDTYGAALLLHMLTSTIKLTLLAYQATKINGVNVYAFTVVGYLGYALAQVFHFCIFGNRLIEESS
SVMEAAYSCHWYDGSEEAKTFVQIVCQQCQKAMSISGAKFFTVSLDLFASVLGAVVTYFMVLVQLK

>DmOR83bIb
MTTSMQPSKYTGLVADLMPNIRAMKYSGLFMHNFTGGSAFMKKVYSSVHLVFLLMQFTFILVNMALNAEE
VNELSGNTITTLFFTHCITKFIYLAVNQKNFYRTLNIWNQVNTHPLFAESDARYHSIALAKMRKLFFLVM
LTTVASATAWTTITFFGDSVKMVVDHETNSSIPVEIPRLPIKSFYPWNASHGMFYMISFAFQIYYVLFSM
IHSNLCDVMFCSWLIFACEQLQHLKGIMKPLMELSASLDTYRPNSAALFRSLSANSKSELIHNEEKDPGT
DMDMSGIYSSKADWGAQFRAPSTLQSFGGNGGGGNGLVNGANPNGLTKKQEMMVRSAIKYWVERHKHVVR
LVAAIGDTYGAALLLHMLTSTIKLTLLAYQATKINGVNVYAFTVVGYLGYALAQVFHFCIFGNRLIEESS
SVMEAAYSCHWYDGSEEAKTFVQIVCQQCQKAMSISGAKFFTVSLDLFASVLGAVVTYFMVLVQLK

>DmOR69aIa
MQLHDHMKYIDLGCKMACIPRYQWKGRPTERQFYASEQRIVFLLGTICQIFQITGVLIYWYCNGRLATET
GTFVAQLSEMCSSFCLTFVGFCNVYAISTNRNQIETLLEELHQIYPRYRKNHYRCQHYFDMAMTIMRIEF
LFYMILYVYYNSAPLWVLLWEHLHEEYDLSFKTQTNTWFPWKVHGSALGFGMAVLSITVGSFVGVGFSIV
TQNLICLLTFQLKLHYDGISSQLVSLDCRRPGAHKELSILIAHHSRILQLGDQVNDIMNFVFGSSLVGAT
IAICMSSVSIMLLDLASAFKYASGLVAFVLYNFVICYMGTEVTLASGKVLPAAFYNNWYEGDLVYRRMLL
ILMMRATKPYMWKTYKLAPVSITTYMATLKFSYQMFTCVRSLK

>DmOR69aIb
MQLEDFMRYPDLVCQAAQLPRYTWNGRRSLEVKRNLAKRIIFWLGAVNLVYHNIGCVMYGYFGDGRTKDP
IAYLAELASVASMLGFTIVGTLNLWKMLSLKTHFENLLNEFEELFQLIKHRAYRIHHYQEKYTRHIRNTF
IFHTSAVVYYNSLPILLMIREHFSNSQQLGYRIQSNTWYPWQVQGSIPGFFAAVACQIFSCQTNMCVNMF
IQFLINFFGIQLEIHFDGLARQLETIDARNPHAKDQLKYLIVYHTKLLNLADRVNRSFNFTFLISLSVSM
ISNCFLAFSMTMFDFGTSLKHLLGLLLFITYNFSMCRSGTHLILTSGKVLPAAFYNNWYEGDLVYRRMLL
ILMMRATKPYMWKTYKLAPVSITTYMATLKFSYQMFTCVRSLK

>DmOR46aIa
MSKGVEIFYKGQKAFLNILSLWPQIERRWRIIHQVNYVHVIVFWVLLFDLLLVLHVMANLSYMSEVVKAI
FILATSAGHTTKLLSIKANNVQMEELFRRLDNEEFRPRGANEELIFAAACERSRKLRDFYGALSFAALSM
ILIPQFALDWSHLPLKTYNPLGENTGSPAYWLLYCYQCLALSVSCITNIGFDSLCSSLFIFLKCQLDILA
VRLDKIGRLITTSGGTVEQQLKENIRYHMTIVELSKTVERLLCKPISVQIFCSVLVLTANFYAIAVLSDE
RLELFKYVTYQACMLIQIFILCYYAGEVTQRSLDLPHELYKTSWVDWDYRSRRIALLFMQRLHSTLRIRT
LNPSLGFDLMLFSSIVNCSYSYFALLKRVNS

>DmOR46aIb
MVTEDFYKYQVWYFQILGVWQLPTWAADHQRRFQSMRFGFILVILFIMLLLFSFEMLNNISQVREILKVF
FMFATEISCMAKLLHLKLKSRKLAGLVDAMLSPEFGVKSEQEMQMLELDRVAVVRMRNSYGIMSLGAASL
ILIVPCFDNFGELPLAMLEVCSIEGWICYWSQYLFHSICLLPTCVLNITYDSVAYSLLCFLKVQLQMLVL
RLEKLGPVIEPQDNEKIAMELRECAAYYNRIVRFKDLVELFIKGPGSVQLMCSVLVLVSNLYDMSTMSIA
NGDAIFMLKTCIYQLVMLWQIFIICYASNEVTVQSSRLCHSIYSSQWTGWNRANRRIVLLMMQRFNSPML
LSTFNPTFAFSLEAFGSIVNCSYSYFALLKRVNS
```

>DmOR63aIa
MYSPEEAAELKRRNYRSIREMIRLSYTVGFNLLDPSRCGQVLRIWTIVLSVSSLASLYGHWQMLARYIHD
IPRIGETAGTALQFLTSIAKMWYFLFAHRQIYELLRKARCHELLQKCELFERMSDLPVIKEIRQQVESTM
NRYWASTRRQILIYLYSCICITTNYFINSFVINLYRYFTKPKGSYDIMLPLPSLYPAWEHKGLEFPYYHI
QMYLETCSLYICGMCAVSFDGVFIVLCLHSVGLMRSLNQMVEQATSELVPPDRRVEYLRCCIYQYQRVAN
FATEVNNCFRHITFTQFLLSLFNWGLALFQMSVGLGNNSSITMIRMTMYLVAAGYQIVVYCYNGQRFATA
SEEIANAFYQVRWYGESREFRHLIRMMLMRTNRGFRLDVSWFMQMSLPTLMAMVRTSGQYFLLLQNVNQK

>DmOR63aIb
MYSPEEAAELKRRNYRSIREMIRLSYTVGFNLLDPSRCGQVLRIWTIVLSVSSLASLYGHWQMLARYIHD
IPRIGETAGTALQFLTSIAKMWYFLFAHRQIYELLRKARCHELLQKCELFERMSDLPVIKEIRQQVESTM
NRYWASTRRQILIYLYSCICITTNYFINSFVINLYRYFTKPKGSYDIMLRK

>DmOR43b
MFGHFKLVYPAPISEPIQSRDSNAYMMETLRNSGLNLKNDFGIGRKIWRVFSFTYNMVILPVSFPINYVI
HLAEFPPELLLQSLQLCLNTWCFALKFFTLIVYTHRLELANKHFDELDKYCVKPAEKRKVRDMVATITRL
YLTFVVVYVLYATSTLLDGLLHHRVPYNTYYPFINWRVDRTQMYIQSFLEYFTVGYAIYVATATDSYPVI
YVAALRTHILLLKDRIIYLGDPSNEGSSDPSYMFKSLVDCIKAHRTMLNFCDAIQPIISGTIFAQFIICG
SILGIIMINMVLFADQSTRFGIVIYVMAVLLQTFPLCFYCNAIVDDCKELAHALFHSAWWVQDKRYQRTV
IQFLQKLQQPMTFTAMNIFNINLATNINVAKFAFTVYAIASGMNLDQKLSIKE

>DmOR23a
MKLSETLKIDYFRVQLNAWRICGALDLSEGRYWSWSMLLCILVYLPTPMLLRGVYSFEDPVENNFSLSLT
VTSLSNLMKFCMYVAQLTKMVEVQSLIGQLDARVSGESQSERHRNMTEHLLRMSKLFQITYAVVFIIAAV
PFVFETELSLPMPMWFPFDWKNSMVAYIGALVFQEIGYVFQIMQCFAADSFPPLVLYLISEQCQLLILRI
SEIGYGYKTLEENEQDLVNCIRDQNALYRLLDVTKSLVSYPMMVQFMVIGINIAITLFVLIFYVETLYDR
IYYLCFLLGITVQTYPLCYYGTMVQESFAELHYAVFCSNWVDQSASYRGHMLILAERTKRMQLLLAGNLV
PIHLSTYVACWKGAYSFFTLMADRDGLGS

>DmOR22b
MLSQFFPHIKEKPLSERVKSRDAFVYLDRVMWSFGWTVPENKRWDLHYKLWSTFVTLLIFILLPISVSVE
YIQRFKTFSAGEFLSSIQIGVNMYGSSFKSYLTMMGYKKRQEAKMSLDELDKRCVCDEERTIVHRHVALG
NFCYIFYHIAYTSFLISNFLSFIMKRIHAWRMYFPYVDPEKQFYISSIAEVILRGWAVFMDLCTDVCPLI
SMVIARCHITLLKQRLRNLRSEPGRTEDEYLKELADCVRDHRLILDYVDALRSVFSGTIFVQFLLIGIVL
GLSMINIMFFSTLSTGVAVVLFMSCVSMQTFPFCYLCNMIMDDCQEMADSLFQSDWTSADRRYKSTLVYF
LHNLQQPIILTAGGVFPISMQTNLNMVKLAFTVVTIVKQFNLAEKFQ

>DmOR22a
MLSKFFPHIKEKPLSERVKSRDAFIYLDRVMWSFGWTEPENKRWILPYKLWLAFVNIVMLILLPISISIE
YLHRFKTFSAGEFLSSLEIGVNMYGSSFKCAFTLIGFKKRQEAKVLLDQLDKRCLSDKERSTVHRYVAMG
NFFDILYHIFYSTFVVMNFPYFLLERRHAWRMYFPYIDSDEQFYISSIAECFLMTEAIYMDLCTDVCPLI
SMLMARCHISLLKQRLRNLRSKPGRTEDEYLEELTECIRDHRLLLDYVDALRPVFSGTIFVQFLLIGTVL
GLSMINLMFFSTFWTGVATCLFMFDVSMETFPFCYLCNMIIDDCQEMSNCLFQSDWTSADRRYKSTLVYF
LHNLQQPITLTAGGVFPISMQTNLAMVKLAFSVVTVIKQFNLAERFQ

>DmOR82a
MGRLFQLQEYCLRAMGHKDDMDSTDSTALSLKHISSLIFVISAQYPLISYVAYNRNDMEKVTACLSVVFT
NMLTVIKISTFLANRKDFWEMIHRFRKMHEQSASHIPRYREGLDYVAEANKLASFLGRAYCVSCGLTGLY
FMLGPIVKIGVCRWHGTTCDKELPMPMKFPFNDLESPGYEVCFLYTVLVTVVVVAYASAVDGLFISFAIN
LRAHFQTLQRQIENWEFPSSEPDTQIRLKSIVEYHVLLLSLSRKLRSIYTPTVMGQFVITSLQVGVIIYQ
LVTNMDSVMDLLLYASFFGSIMLQLFIYCYGGEIIKAESLQVDTAVRLSNWHLASPKTRTSLSLIILQSQ
KEVLIRAGFFVASLANFVGICRTALSLITLIKSIE

>DmOR43a
MTIEDIGLVGINVRMWRHLAVLYPTPGSSWRKFAFVLPVTAMNLMQFVYLLRMWGDLPAFILNMFFFSAI
FNALMRTWLVIIKRRQFEEFLGQLATLFHSILDSTDEWGRGILRRAEREARNLAILNLSASFLDIVGALV
SPLFREERAHPFGLALPGVSMTSSPVYEVIYLAQLPTPLLLSMMYMPFVSLFAGLAIFGKAMLQILVHRL
GQIGGEEQSEEERFQRLASCIAYHTQVMRYVWQLNKLVANIVAVEAIIFGSIICSLLFCLNIITSPTQVI

SIVMYILTMLYVLFTYYNRANEICLENNRVAEAVYNVPWYEAGTRFRKTLLIFLMQTQHPMEIRVGNVYP
MTLAMFQSLLNASYSYFTMLRGVTG

>DmOR2a
MEKQEDFKLNTHSAVYYHWRVWELTGLMRPPGVSSLLYVVYSITVNLVVTVLFPLSLLARLLFTTNMAGL
CENLTITITDIVANLKFANVYMVRKQLHEIRSLLRLMDARARLVGDPEEISALRKEVNIAQGTFRTFASI
FVFGTTLSCVRVVVRPDRELLYPAWFGVDWMHSTRNYVLINIYQLFGLIVQAIQNCASDSYPPAFLCLLT
GHMRALELRVRRIGCRTEKSNKGQTYEAWREEVYQELIECIRDLARVHRLREIIQRVLSVPCMAQFVCSA
AVQCTVAMHFLYVADDHDHTAMIISIVFFSAVTLEVFVICYFGDRMRTQSEALCDAFYDCNWIEQLPKFK
RELLFTLARTQRPSLIYAGNYIALSLETFEQVMRFTYSVFTLLLRAK

>DmOR67d
MLKMAKVEPVERYCKVIRMIRFCVGFCGNDVADPNFRMWWLTYAVMAAIAFFFACTGYTIYVGVVINGDL
TIILQALAMVGSAVQGLTKLLVTANNASHMREVQNTYEDIYREYGSKGDEYAKCLEKRIRITWTLLIGFM
LVYIILLGLVITFPIFYLLILHQKVLVMQFLIPFLDHTTDGGHLILTAAHVILITFGGFGNYGGDMYLFL
FVTHVPLIKDIFCVKLTEFNELVMKRNDFPKVRAMLCDLLVWHQLYTRMLQTTKKIYSIVLFVQLSTTCV
GLLCTISCIFMKAWPAAPLYLLYAAITLYTFCGLGTLVENSNEDFLSVIYTNCLWYELPVKEEKLIIMML
AKAQNEVVLTAADMAPLSMNTALQLTKGIYSFSMMLMNYLG

>DmOR59c
MTKFFFKRLQTAPLDQEVSSLDASDYYYRIAFFLGWTPPKGALLRWIYSLWTLTTMWLGIVYLPLGLSLT
YVKHFDRFTPTEFLTSLQVDINCIGNVIKSCVTYSQMWRFRRMNELISSLDKRCVTTTQRRIFHKMVARV
NLIVILFLSTYLGFCFLTLFTSVFAGKAPWQLYNPLVDWRKGHWQLWIASILEYCVVSIGTMQELMSDTY
AIVFISLFRCHLAILRDRIANLRQDPKLSEMEHYEQMVACIQDHRTIIQCSQIIRPILSITIFAQFMLVG
IDLGLAAISILFFPNTIWTIMANVSFIVAICTESFPCCMLCEHLIEDSVHVSNALFHSNWITADRSYKSA
VLYFLHRAQQPIQFTAGSIFPISVQSNIAVAKFAFTIITIVNQMNLGEKFFSDRSNGDINP

>DmOR59b
MAVFKLIKPAPLTEKVQSRQGNIYLYRAMWLIGWIPPKEGVLRYVYLFWTCVPFAFGVFYLPVGFIISYV
QEFKNFTPGEFLTSLQVCINVYGASVKSTITYLFLWRLRKTEILLDSLDKRLANDSDRERIHNMVARCNY
AFLIYSFIYCGYAGSTFLSYALSGRPPWSVYNPFIDWRDGMGSLWIQAIFEYITMSFAVLQDQLSDTYPL
MFTIMFRAHMEVLKDHVRSLRMDPERSEADNYQDLVNCVLDHKTILKCCDMIRPMISRTIFVQFALIGSV
LGLTLVNVFFFSNFWKGVASLLFVITILLQTFPFCYTCNMLIDDAQDLSNEIFQSNWVDAEPRYKATLVL
FMHHVQQPIIFIAGGIFPISMNSNITVAKFAFSIITIVRQMNLAEQFQ

>DmOR59a
MAEVRVDSLEFFKSHWTAWRYLGVAHFRVENWKNLYVFYSIVSNLLVTLCYPVHLGISLFRNRTITEDIL
NLTTFATCTACSVKCLLYAYNIKDVLEMERLLRLLDERVVGPEQRSIYGQVRVQLRNVLYVFIGIYMPCA
LFAELSFLFKEERGLMYPAWFPFDWLHSTRNYYIANAYQIVGISFQLLQNYVSDCFPAVVLCLISSHIKM
LYNRFEEVGLDPARDAEKDLEACITDHKHILELFRRIEAFISLPMLIQFTVTALNVCIGLAALVFFVSEP
MARMYFIFYSLAMPLQIFPSCFFGTDNEYWFGRLHYAAFSCNWHTQNRSFKRKMMLFVEQSLKKSTAVAG
GMMRIHLDTFFSTLKGAYSLFTIIIRMRK

>DmOR88a
MKPTEIKKPYRMEEFLRPQMFQEVAQMVHFQWRRNPVDNSMVNASMVPFCLSAFLNVLFFGCNGWDIIGH
FWLGHPANQNPPVLSITIYFSIRGLMLYLKRKEIVEFVNDLDRECPRDLVSQLDMQMDETYRNFWQRYRF
IRIYSHLGGPMFCVVPLALFLLTHEGKDTPVAQHEQLLGGWLPCGVRKDPNFYLLVWSFDLMCTTCGVSF
FVTFDNLFNVMQGHLVMHLGHLARQFSAIDPRQSLTDEKRFFVDLRLLVQRQQLLNGLCRKYNDIFKVAF
LVSNFVGAGSLCFYLFMLSETSDVLIIAQYILPTLVLVGFTFEICLRGTQLEKASEGLESSLRSQEWYLG
SRRYRKFYLLWTQYCQRTQQLGAFGLIQVNMVHFTEIMQLAYRLFTFLKSH

>DmOR22c
MTDSGQPAIADHFYRIPRISGLIVGLWPQRIRGGGGRPWHAHLLFVFAFAMVVVGAVGEVSYGCVHLDNL
VVALEAFCPGTTKAVCVLKLWVFFRSNRRWAELVQRLRAILWESRRQEAQRMLVGLATTANRLSLLLLSS
GTATNAAFTLQPLIMGLYRWIVQLPGQTELPFNIILPSFAVQPGVFPLTYVLLTASGACTVFAFSFVDGF
FICSCLYICGAFRLVQQDIRRIFADLHGDSVDVFTEEMNAEVRHRLAQVVERHNAIIDFCTDLTRQFTVI
VLMHFLSAAFVLCSTILDIMLNTSSLSGLTYICYIIAALTQLFLYCFGGNHVSESSAAVADVLYDMEWYK
CDARTRKVILMILRRSQRAKTIAVPFFTPSLPALRSILSTAGSYITLLKTFL

>DmOR56a
MFKDLLLSPTTFEDPIFGTHLRYFQWYGYVASKDQNRPLLSLIRCTILTASIWLSCALMLARVFRGYE
NLNDGATSYATAVQYFAVSIAMFNAYVQRDKVISLLRVAHSDIQNLMHEADNREMELLVATQAYTRTITL
LIWIPSVIAGLMAYSDCIYRSLFLPKSVFNVPAVRRGEEHPILLFQLFPFGELCDNFVVGYLGPWYALGL
GITAIPLWHTFITCLMKYVNLKLQILNKRVEEMDITRLNSKLVIGRLTASELTFWQMQLFKEFVKEQLRI
RKFVQELQYLICVPVMADFIIFSVLICFLFFALTVGVPSKMDYFFMFIYLFVMAGILWIYHWHATLIVEC
HDELSLAYFSCGWYNFEMPLQKMLVFMMMHAQRPMKMRALLVDLNLRTFIDIGRGAYSYFNLLRSSHLY

>DmOR85f
MEPVQYSYEDFARLPTTVFWIMGYDMLGVPKTRSRRILYWIYRFLCLASHGVCVGVMVFRMVEAKTIDNV
SLIMRYATLVTYIINSDTKFATVLQRSAIQSLNSKLAELYPKTTLDRIYHRVNDHYWTKSFVYLVIIYIG
SSIMVVIGPIITSIIAYFTHNVFTYMHCYPYFLYDPEKDPVWIYISIYALEWLHSTQMVISNIGADIWLL
YFQVQINLHFRGIIRSLADHKPSVKHDQEDRKFIAKIVDKQVHLVSLQNDLNGIFGKSLLLSLLTTAAVI
CTVAVYTLIQGPTLEGFTYVIFIGTSVMQVYLVCYYGQQVLDLSGEVAHAVYNHDFHDASIAYKRYLLII
IIRAQQPVELNAMGYLSISLDTFKQLMSVSYRVITMLMQMIQ

>DmOR85d
MLTKKDTQSAKEQEKLKAIPLHSFLKYANVFYLSIGMMAYDHKYSQKWKEVLLHWTFIAQMVNLNTVLIS
ELIYVFLAIGKGSNFLEATMNLSFIGFVIVGDFKIWNISRQRKRLTQVVSRLEELHPQGLAQQEPYNIGH
HLSGYSRYSKFYFGMHMVLIWTYNLYWAVYYLVCDFWLGMRQFERMLPYYCWVPWDWSTGYSYYFMYISQ
NIGGQACLSGQLAADMLMCALVTLVVMHFIRLSAHIESHVAGIGSFQHDLEFLQATVAYHQSLIHLCQDI
NEIFGVSLLSNFVSSSFIICFVGFQMTIGSKIDNLVMLVLFLFCAMVQVFMIATHAQRLVDASEQIGQAV
YNHDWFRADLRYRKMLILIIKRAQQPSRLKATMFLNISLVTVSDLLQLSYKFFALLRTMYVN

>DmOR85c
MKFMKYAVFFYTSVGIEPYTIDSRSKKASLWSHLLFWANVINLSVIVFGEILYLGVAYSDGKFIDAVTVL
SYIGFVIVGMSKMFFIWWKKTDLSDLVKELEHIYPNGKAEEEMYRLDRYLRSCSRISITYALLYSVLIWT
FNLFSIMQFLVYEKLLKIRVVGQTLPYLMYFPWNWHENWTYYVLLFCQNFAGHTSASGQISTDLLLCAVA
TQVVMHFDYLARVVEKQVLDRDWSENSRFLAKTVQYHQRILRLMDVLNDIFGIPLLLNFMVSTFVICFVG
FQMTVGVPPDIMIKLFLFLFSSLSQVYLICHYGQLIADASSSLSISAYKQNWQNADIRYRRALVFFIARP
QRTTYLKATIFMNITRATMTDLLQVSYKFFALLRTMYIK

>DmOR85b
MEKLMKYASFFYTAVGIRPYTNGEESKMNKLIFHIVFWSNVINLSFVGLFESIYVYSAFMDNKFLEAVTA
LSYIGFVTVGMSKMFFIRWKKTAITELINELKEIYPNGLIREERYNLPMYLGTCSRISLIYSLLYSVLIW
TFNLFCVMEYWVYDKWLNIRVVGKQLPYLMYIPWKWQDNWSYYPLLFSQNFAGYTSAAGQISTDVLLCAV
ATQLVMHFDFLSNSMERHELSGDWKKDSRFLVDIVRYHERILRLSDAVNDIFGIPLLLNFMVSSFVICFV
GFQMTVGVPPDIVVKLFLFLVSSMSQVYLICHYGQLVADASYGFSVATYNQKWYKADVRYKRALVIIIAR
SQKVTFLKATIFLDITRSTMTDLLQISYKFFALLRTMYTQ

>DmOR85a
MIFKYIQEPVLGSLFRSRDSLIYLNRSIDQMGWRLPPRTKPYWWLYYIWTLVVIVLVFIFIPYGLIMTGI
KEFKNFTTTDLFTYVQVPVNTNASIMKGIIVLFMRRRFSRAQKMMDAMDIRCTKMEEKVQVHRAAALCNR
VVVIYHCIYFGYLSMALTGALVIGKTPFCLYNPLVNPDDHFYLATAIESVTMAGIIILANLILDVYPIIYV
VVLRIHMELLSERIKTLRTDVEKGDDQHYAELVECVKDHKLIVEYGNTLRPMISATMFIQLLSVGLLLGL
AAVSMQFYNTVMERVVSGVYTIAILSQTFPFCYVCEQLSSDCESLTNTLFHSKWIGAERRYRTTMLYFIH
NVQQSILFTAGGIFPICLNTNIKMAKFAFSVVTIVNEMDLAEKLRRE

>DmOR13a
MFYSYPYKALSFPIQCVWLKLNGSWPLTESSRPWRSQSLLATAYIVWAWYVIASVGITISYQTAFLLNNL
SDIIITTENCCTTFMGVLNFVRLIHLRLNQRKFRQLIENFSYEIWIPNSSKNNVAAECRRRMVTFSIMTS
LLACLIIMYCVLPLVEIFFGPAFDAQNKPFPYKMIFPYDAQSSWIRYVMTYIFTSYAGICVVTTLFAEDT
ILGFFITYTCGQFHLLHQRIAGLFAGSNAELAESIQLERLKRIVEKHNNIISFAKRLEDFFNPILLANLM
ISSVLICMVGFQIVTGKNMFIGDYVKFIIYISSALSQLYVLCENGDALIKQSTLTAQILYECQWEGSDRI
EIQSFTPTTKRIRNQIWFMILCSQQPVRITAFKFSTLSLQSFTAILSTSISYFTLLRSVYFDDEKKLD

>DmOR65c

MDIRGNVHRFVKFYIDGWKHFRDPTMESSYSAVYYWREQMKAMFLYTTSKERQMPYRSSWHTLVIIQATV
CFLTMCYGVTESLGDKVQMGRDIAFIIGFFYIAFKIYYFQWYGDELDEVVEALETFHPWAQKGPGAVDYR
TAKRWYFTLAFFLASSWLVFLCIFILLLITSPLWVHQQILPLHAAFPFQWHEKSIHPISHAFIYLFQTWN
VMYFLTWLVCIEGLSVSIYVEITFAIEVLCLELRHLHQRCHGYEQLRLETNRLVQFHQKIVHILDHTNKV
FHGTLIMQMGVNFFLVSLSVLEAMEARKDPKVVAQFAVLMLLALGHLSMWSYFGDLLSQKSLTISEAAYE

AYDPIKGSKDVYRDLCLIIRRGQEPLIMRASPFPSFNFINYSAILNQCYGILTFLLKTLD

>DmOR65b
MDIQRFLKFYKVGWKTYRDPLMEASHSSIYYWREQMKAMALFTTTEERLLPYRSKWHTLVYIQMVIFFAS
MSFGLTESMGDHVQMGRDLAFILGAFFIIFKTYYFCWYGDELDQVISDLDALHPWAQKGPNPVEYQTGKR
WYFVMAFFLATSWSFFLCILLLLLITSPMWVHQQNLPFHAAFPFQWHEKSLHPISHAIIYLFQSYFAVYC
LTWLLCIEGLSICIYAEITFGIEVLCLELRQIHRHNYGLQELRMETNRLVKLHQKIVEILDRTNDVFHGT
LIMQMGVNFSLVSLSVLEAVEARKDPKVVAQFAVLMLLALGHLSMWSYCGDQLSQKSLQISEAAYEAYDP
TKGSKDVYRDLCVIIRRGQDPLIMRASPFPSFNLINYSAILNQCYGILTFLLKTLD

>DmOR65a
MTELRSERKNGNWDRLFGPFFESWAVFKAPQAKSRHIIAYWTRDQLKALGFYMNSEQRRLPRIVAWQYFV
SIQLATALASLFYGISESIGDIVNLGRDLVFIITIIFICFRLVFFAQYAGELDVIIDALEDIYHWSIKGP
ATKEVQETKRLHFLLFMALIITWFSFLILFMLIKISTPFWIESQTLPFHVSWPFQLHDPSKHPIAYIIIF
VSQSTTMLYFLIWLGVVENMGVSLFFELTSALRVLCIELRNLQELCLGDEDMLYRELCRMTKFHQQIILL
TDRCNHIFNGAFIMQMLINFLLVSLSLFEVLAAKKNPQVAVEYMIIMLMTLGHLSFWSKFGDMFSKESEQ
VALAVYEAYDPNVGSKSIHRQFCFFIQRAQKPLIMKASPFPPFNLENYMFILKQCYSILTILANTLE

>DmOR83c
MSTSESPSSRFRELSKYINSLTNLLGVDFLSPKLKFNYRTWTTIFAIANYTGFTVFTILNNGGDWRVGLK
ASLMTGGLFHGLGKFLTCLLKHQDMRRLVLYSQSIYDEYETRGDSYHRTLNSNIDRLLGIMKIIRNGYVF
AFCLMELLPLAMLMYDGTRVTAMQYLIPGLPLENNYCYVVTYMIQTVTMLVQGVGFYSGDLFVFLGLTQI
LTFADMLQVKVKELNDALEQKAEYRALVRVGASIDGAENRQRLLLDVIRWHQLFTDYCRAINALYYELIA
TQVLSMALAMMLSFCINLSSFHMPSAIFFVVSAYSMSIYCILGTILEFAYDQVYESICNVTWYELSGEQR
KLFGFLLRESQYPHNIQILGVMSLSVRTALQIVKLIYSVSMMMMNRA

>DmOR83a
MKSTFKEERIKDDSKRRDLFVFVRQTMCIAAMYPFGYYVNGSGVLAVLVRFCDLTYELFNYFVSVHIAGL
YICTIYINYGQGDLDFFVNCLIQTIIYLWTIAMKLYFRRFRPGLLNTILSNINDEYETRSAVGFSFVTMA
GSYRMSKLWIKTYVYCCYIGTIFWLALPIAYRDRSLPLACWYPFDYTQPGVYEVVFLLQAMGQIQVAASF
ASSSGLHMVLCVLISGQYDVLFCSLKNVLASSYVLMGANMTELNQLQAEQSAADVEPGQYAYSVEEETPL
QELLKVGSSMDFSSAFRLSFVRCIQHHRYIVAALKKIESFYSPIWFVKIGEVTFLMCLVAFVSTKSTAAN
SFMRMVSLGQYLLLVLYELFIICYFADIVFQNSQRCGEALWRSPWQRHLKDVRSDYMFFMLNSRRQFQLT
AGKISNLNVDRFRGTITTAFSFLTLLQKMDARE

>DmOR49b
MFEDIQLIYMNIKILRFWALLYDKNLRRYVCIGLASFHIFTQIVYMMSTNEGLTGIIRNSYMLVLWINTV
LRAYLLLADHDRYLALIQKLTEAYYDLLNLNDSYISEILDQVNKVGKLMARGNLFFGMLTSMGFGLYPLS
SSERVLPFGSKIPGLNEYESPYYEMWYIFQMLITPMGCCMYIPYTSLIVGLIMFGIVRCKALQHRLRQVA
LKHPYGDRDPRELREEIIACIRYQQSIIEYMDHINELTTMMFLFELMAFSALLCALLFMLIIVSGTSQLI
IVCMYINMILAQILALYWYANELREQNLAVATAAYETEWFTFDVPLRKNILFMMMRAQRPAAILLGNIRP
ITLELFQNLLNTTYTFFTVLKRVYG

>DmOR49a
MEKLRSYEDFIFMANMMFKTLGYDLFHTPKPWWRYLLVRGYFVLCTISNFYEASMVTTRIIEWESLAGSP
SKIMRQGLHFFYMLSSQLKFITFMINRKRLLQLSHRLKELYPHKEQNQRKYEVNKYYLSCSTRNVLYVYY
FVMVVMALEPLVQSCIMYLIGFGKADFTYKRIFPTRLTFDSEKPLGYVLAYVIDFTYSQFIVNVSLGTDL
WMMCVSSQISMHLGYLANMLASIRPSPETEQQDCDFLASIIKRHQLMIRLQKDVNYVFGLLLASNLFTTS
CLLCCMAYYTVVEGFNWEGISYMMLFASVAAQFYVVSSHGQMLIDLSTNLAKAAFESKWYEGSLRYKKEI
LILMAQAQRPLEISARGVIIISLDTFKILMTITYRFFAVIRQTVEK

>DmOR47b
MNDSGYQSNLSLLRVFLDEFRSVLRQESPGLIPRLAFYYVRAFLSLLCQYPNKKLASLPLYRWINLFIMC
NVMTIFWTMFVALPESKNVIEMGDDLVWISGMALVFTKIFYMHLRCDEIDELISDFEYYNRELRPHNIDE
EVLGWQRLCYVIESGLYINCFCLVNFFSAAIFLQPLLGEGKLPFHSVYPFQWHRLDLHPYTFWFLYIWQS
LTSQHNLMSILMVDMVGISTFLQTALNLKLLCIEIRKLGDMEVSDKRFHEEFCRVVRFHQHIIKLVGKAN
RAFNGAFNAQLMASFSLISISTFETMAAAAVDPKMAAKFVLLMLVAFIQLSLWCVSGTLVYTQSVEVAQA
AFDINDWHTKSPGIQRDISFVILRAQKPLMYVAEPFLPFTLGTYMLVLKNCYRLLALMQESM

>DmOR10a
MSEWLRFLKRDQQLDVYFFAVPRLSLDIMGYWPGKTGDTWPWRSLIHFAILAIGVATELHAGMCFLDRQQ
ITLALETLCPAGTSAVTLLKMFLMLRFRQDLSIMWNRLRGLLFDPNWERPEQRDIRLKHSAMAARINFWP
LSAGFFTCTTYNLKPILIAMILYLQNRYEDFVWFTPFNMTMPKVLLNYPFFPLTYIFIAYTGYVTIFMFG
GCDGFYFEFCAHLSALFEVLQAEIESMFRPYTDHLELSPVQLYILEQKMRSVIIRHNAIIDLTRFFRDRY
TIITLAHFVSAAMVIGFSMVNLLTLGNNGLGAMLYVAYTVAALSQLLVYCYGGTLVAESSTGLCRAMFSC
PWQLFKPKQRRLVQLLILRSQRPVSMAVPFFSPSLATFAAILQTSGSIIALVKSFQ

>DmOR47a
MDSFLQVQKSTIALLGFDLFSENREMWKRPYRAMNVFSIAAIFPFILAAVLHNWKNVLLLADAMVALLIT
ILGLFKFSMILYLRRDFKRLIDKFRLLMSNEAEQGEEYAEILNAANKQDQRMCTLFRTCFLLAWALNSVL
PLVRMGLSYWLAGHAEPELPFPCLFPWNIHIIRNYVLSFIWSAFASTGVVLPAVSLDTIFCSFTSNLCAF
FKIAQYKVVRFKGGSLKESQATLNKVFALYQTSLDMCNDLNQCYQPIICAQFFISSLQLCMLGYLFSITF
AQTEGVYYASFIATIIIQAYIYCYCGENLKTESASFEWAIYDSPWHESLGAGGASTSICRSLLISMMRAH
RGFRITGYFFEANMEAFSSIVRTAMSYITMLRSFS

>DmOR9a
MSDKVKGKKQEEKDQSLRVQILVYRCMGIDLWSPTMANDRPWLTFVTMGPLFLFMVPMFLAAHEYITQVS
LLSDTLGSTFASMLTLVKFLLFCYHRKEFVGLIYHIRAILAKEIEVWPDAREIIEVENQSDQMLSLTYTR
CFGLAGIFAALKPFVGIILSSIRGDEIHLELPHNGVYPYDLQVVMFYVPTYLWNVMASYSAVTMALCVDS
LLFFFTYNVCAIFKIAKHRMIHLPAVGGKEELEGLVQVLLLHQKGLQIADHIADKYRPLIFLQFFLSALQ
ICFIGFQVADLFPNPQSLYFIAFVGSLLIALFIYSKCGENIKSASLDFGNGLYETNWTDFSPPTKRALLI
AAMRAQRPCQMKGYFFEASMATFSTIVRSAVSYIMMLRSFNA

>DmOR45b
MYPRFLSRNYPLAKHLFFVTRYSFGLLGLRFGKEQSWLHLLWLVFNFVNLAHCCQAEFVFGWSHLRTSPV
DAMDAFCPLACSFTTLFKLGWMWWRRQEVADLMDRIRLLIGEQEKREDSRRKVAQRSYYLMVTRCGMLVF
TLGSITTGAFVLRSLWEMWVRRHQEFKFDMPFRMLFHDFAHRMPWFPVFYLYSTWSGQVTVYAFAGTDGF
FFGFTLYMAFLLQALRYDIQDALKPIRDPSLRESKICCQRLADIVDRHNEIEKIVKEFSGIMAAPTFVHF
VSASLVIATSVIDILLYSGYNIIRYVVYTFTVSSAIFLYCYGGTEMSTESLSLGEAAYSSAWYTWDRETR
RRVFLIIILRAQRPITVRVPFFAPSLPVFTSVIKFTGSIVALAKTIL

>DmOR45a
MDASYFAVQRRALEIVGFDPSTPQLSLKHPIWAGILILSLISHNWPMVVYALQDLSDLTRLTDNFAVFMQ
GSQSTFKFLVMMAKRRRIGSLIHRLHKLNQAASATPNHLEKIERENQLDRYVARSFRNAAYGVICASAIA
PMLLGLWGYVETGVFTPTTPMEFNFWLDERKPHFYWPIYVWGVLGVAAAAWLAIATDTLFSWLTHNVVIQ
FQLLELVLEEKDLNGGDSRLTGFVSRHRIALDLAKELSSIFGEIVFVKYMLSYLQLCMLAFRFSRSGWSA
QVPFRATFLVAIIIQLSSYCYGGEYIKQQSLAIAQAVYGQINWPEMTPKKRRLWQMVIMRAQRPAKIFGF
MFVVDLPLLLWVIRTAGSFLAMLRTFER

>DmOR7a
MAVSTRVATKQEVPESRRAFRNLFNCFYALGMQAPDGSRPTTSSTWQRIYACFSVVMYVWQLLLVPTFFV
ISYRYMGGMEITQVLTSAQVAIDAVILPAKIVALAWNLPLLRRAEHHLAALDARCREQEEFQLILDAVRF
CNYLVWFYQICYAIYSSSTFVCAFLLGQPPYALYLPGLDWQRSQMQFCIQAWIEFLIMNWTCLHQASDDV
YAVIYLYVVRIQVQLLARRVEKLGTDDSGQVEIYPDERRQEEHCAELQRCIVDHQTMLQLLDCISPVISR
TIFVQFLITAAIMGTTMINIFIFANTNTKIASIIYLLAVTLQTAPCCYQATSLMLDNERLALAIFQCQWL
GQSARFRKMLLYYLHRAQQPITLTAMKLFPINLATYFSIAKFSFSLYTLIKGMNLGERFNRTN

>DmOR74a
MSFHRYRPRLPGGELAPMPWPVSLYRVLNHVAWPLEAESGRWTVFLDRLMIFLGFLVFCEHNEVDFHYLI

ANRQDMDNMLTGLPTYLILVEMQIRCFQLAWHKDRFRALLQRFYAEIYVSEEMEPHLFASIQRQMLATRV
NSTVYLLALLNFFLVPVTNVIYHRREMLYKQVYPFDNTQLHFFIPLLVLNFWVGFIITSMLFGELNVMGE
LMMHLNARYIQLGQDLRRSAQMLLKKSSSLNVAIAYRLNLTHILRRNAALRDFGQRVEKEFTLRIFVMFA
FSAGLLCALFFKAFTNPWGNVAYIVWFLAKFMELLALGMLGSILLKTTDELGMMYYTADWEQVIHQSDNV
GENVKLMKLVTLAIQLNSRPFFITGLNYFRVSLTAVLKIIQGAFSYFTFLNSMR

>DmOR42b
MVFELIRPAPLTEQKRSRDGCIYLYRAMKFIGWLPPKQGVLRYVYLTWTLMTFVWCTTYLPLGFLGSYMT
QIKSFSPGEFLTSLQVCINAYGSSVKVAITYSMLWRLIKAKNILDQLDLRCTAMEEREKIHLVVARSNHA
FLIFTFVYCGYAGSTYLSSVLSGRPPWQLYNPFIDWHDGTLKLWVASTLEYMVMSGAVLQDQLSDSYPLI
YTLILRAHLDMLRERIRRLRSDENLSEAESYEELVKCVMDHKLILRYCAIIKPVIQGTIFTQFLLIGLVL
GFTLINVFFFSDIWTGIASFMFVITILLQTFPFCYTCNLIMEDCESLTHAIFQSNWVDASRRYKTTLLYF
LQNVQQPIVFIAGGIFQISMSSNISVAKFAFSVITITKQMNIADKFKTD

>DmOR42a
MDLRRWFPTLYTQSKDSPVRSRDATLYLLRCVFLMGVRKPPAKFFVAYVLWSFALNFCSTFYQPIGFLTG
YISHLSEFSPGEFLTSLQVAFNAWSCSTKVLIVWALVKRFDEANNLLDEMDRRITDPGERLQIHRAVSLS
NRIFFFFMAVYMVYATNTFLSAIFIGRPPYQNYYPFLDWRSSTLHLALQAGLEYFAMAGACFQDVCVDCY
PVNFVLVLRAHMSIFAERLRRLGTYPYESQEQKYERLVQCIQDHKVILRFVDCLRPVISGTIFVQFLVVG
LVLGFTLINIVLFANLGSAIAALSFMAAVLLETTPFCILCNYLTEDCYKLADALFQSNWIDEEKRYQKTL
MYFLQKLQQPITFMAMNVFPISVGTNISVTKFSFSVFTLVKQMNISEKLAKSEMEE

>DmOR71a
MDYDRIRPVRFLTGVLKWWRLWPRKESVSTPDWTNWQAYALHVPFTFLFVLLLWLEAIKSRDIQHTADVL
LICLTTTALGGKVINIWKYAHVAQGILSEWSTWDLFELRSKQEVDMWRFEHRRFNRVFMFYCLCSAGVIP
FIVIQPLFDIPNRLPFWMWTPFDWQQPVLFWYAFIYQATTIPIACACNVTMDAVNWYLMLHLSLCLRMLG
QRLSKLQHDDKDLREKFLELIHLHQRLKQQALSIEIFISKSTFTQILVSSLIICFTIYSMQMSPVLQDLP
GFAAMMQYLVAMIMQVMLPTIYGNAVIDSANMLTDSMYNSDWPDMNCRMRRLVLMFMVYLNRPVTLKAGG
FFHIGLPLFTKTMNQAYSLLALLLNMNQ

>DmOR1a
MSKLIEVFLGNLWTQRFTFARMGLDLQPDKKGNVLRSPLLYCIMCLTTSFELCTVCAFMVQNRNQIVLCS
EALMHGLQMVSSLLKMAIFLAKSHDLVDLIQQIQSPFTEEDLVGTEWRSQNQRGQLMAAIYFMMCAGTSV
SFLLMPVALTMLKYHSTGEFAPVSSFRVLLPYDVTQPHVYAMDCCLMVFVLSFFCCSTTGVDTLYGWCAL
GVSLQYRRLGQQLKRIPSCFNPSRSDFGLSGIFVEHARLLKIVQHFNYSFMEIAFVEVVIICGLYCSVIC
QYIMPHTNQNFAFLGFFSLVVTTQLCIYLFGAEQVRLEAERFSRLLYEVIPWQNLPPKHRKLFLFPIERA
QRETVLGAYFFELGRPLLVWIFRTAGSFTTLMNALYAKYETH

>DmOR98b
MLTDKFLRLQSALFRLLGLELLHEQDVGHRYPWRSICCILSVASFMPLTIAFGLQNVQNVEQLTDSLCSV
LVDLLALCKIGLFLWLYKDFKFLIGQFYCVLQTETHTAVAEMIVTRESRRDQFISAMYAYCFITAGLSAC
LMSPLSMLISYQRTGELQPKFPFPSVYPWDNMKLSNYIISYFWNVCAALGVALPTVCVDTLFCSLSHNLC
ALFQIARHKMMHFEGRNTKETHENLKHVFQLYALCLNLGHFLNEYFRPLICQFVAASLHLCVLCYQLSAN
ILQPALLFYAAFTAAVVGQVSIYCFCGSSIHSECQLFGQAIYESSWPHLLQENLQLVSSLKIAMMRSSLG
CPIDGYFFEANRETLITVSKAFIKVSKKTPQVND

>DmOR98a
MLFNYLRKPNPTNLLTSPDSFRYFEYGMFCMGWHTPATHKIIYYITSCLIFAWCAVYLPIGIIISFKTDI
NTFTPNELLTVMQLFFNSVGMPFKVLFFNLYISGFYKAKKLLSEMDKRCTTLKERVEVHQGVVRCNKAYL
IYQFIYTAYTISTFLSAALSGKLPWRIYNPFVDFRESRSSFWKAALNETALMLFAVTQTLMSDIYPLLYG
LILRVHLKLLRLRVESLCTDSGKSDAENEQDLIKCIKDHNLIIDYAAAIRPAVTRTIFVQFLLIGICLGL
SMINLLFFADIWTGLATVAYINGLMVQTFPFCFVCDLLKKDCELLVSAIFHSNWINSSRSYKSSLRYFLK
NAQKSIAFTAGSIFPISTGSNIKVAKLAFSVVTFVNQLNIADRLTKN

>DmOR67c
METAKDNTARTFMELMRVPVQFYRTIGEDIYAHRSTNPLKSLLFKIYLYAGFINFNLLVIGELVFFYNSI
QDFETIRLAIAVAPCIGFSLVADFKQAAMIRGKKTLIMLLDDLENMHPKTLAKQMEYKLPDFEKTMKRVI
NIFTFLCLAYTTTFSFYPAIKASVKFNFLGYDTFDRNFGFLIWFPFDATRNNLIYWIMYWDIAHGAYLAG

IAFLCADLLLVVVITQICMHFNYISMRLEDHPCNSNEDKENIEFLIGIIRYHDKCLKLCEHVNDLYSFSL
LLNFLMASMQICFIAFQVTESTVEVIIIYCIFLMTSMVQVFMVCYYGDTLIAASLKVGDAAYNQKWFQCS
KSYCTMLKLLIMRSQKPASIRPPTFPPISLVTYMKVISMSYQFFALLRTTYSNN

>DmOR67b
MQDQLDHELERIDKLPKLGLLWVEYSAYALGVNIAPRKRSSKYCRLTRILVLIVNLSIIYSLVAFIMENY
MISFETYVEAVLLTFQLSVGVVKMFHFQNKVESCSQLVFSTETGEVLKSLGLFQLDLPRKKELLSSVSLI
LLNNWMIIDRQVMFFFKIVCMPVLYYCVRPYFQYIFDCYIKDKDTCEMTLTYPAIVPYLQLGNYEFPSYV
IRFFLLQSGPLWCFFAVFGFNSLFVVLTRYESGLIKVLRFLVQNSTSDILVPKDQRVKYLQCCVRLFARI
SSHHNQIENLFKYIILVQCSVSSILICMLLYKISTVLEVGWVWMGMIMVYFVTIALEITLYNVSAQKVES
QSELLFHDWYNCSWYNESREFKFMIKMMLLFSRRTFVLSVGGFTSLSHKFLVQVFRLSANFFLLLRNMNN
K

>DmOR67a
MDNVAEMPEEKYVEVDDFLRLAVKFYNTLGIDPYETGRKRTIWFQIYFALNMFNMVFSFYAEVATLVDRL
RDNENFLESCILLSYVSFVVMGLSKIGAVMKKKPKMTALVRQLETCFPSPSAKVQEEYAVKSWLKRCHIY
TKGFGGLFMIMYFAHALIPLFIYFIQRVLLHYPDAKQIMPFYQLEPWEFRDSWLFYPSYFHQSSAGYTAT
CGSIAGDLMIFAVVLQVIMHYERLAKVLREFKIQAHNAPNGAKEDIRKLQSLVANHIDILRLTDLMNEVF
GIPLLLNFIASALLVCLVGVQLTIALSPEYFCKQMLFLISVLLEVYLLCSFSQRLIDASENVGHAAYDMD
WLGSDKRFKKILIFISMRSQKPVCLKATVVLDLSMPTMSIFLGMSYKFFCAVRTMYQ

>DmOR35a
MVRYVPRFADGQKVKLAWPLAVFRLNHIFWPLDPSTGKWGRYLDKVLAVAMSLVFMQHNDAELRYLRFEA
SNRNLDAFLTGMPTYLILVEAQFRSLHILLHFEKLQKFLEIFYANIYIDPRKEPEMFRKVDGKMIINRLV
SAMYGAVISLYLIAPVFSIINQSKDFLYSMIFPFDSDPLYIFVPLLLTNVWVGIVIDTMMFGETNLLCEL
IVHLNGSYMLLKRDLQLAIEKILVARDRPHMAKQLKVLITKTLRKNVALNQFGQQLEAQYTVRVFIMFAF
AAGLLCALSFKAYTNPMANYIYAIWFGAKTVELLSLGQIGSDLAFTTDSLSTMYYLTHWEQILQYSTNPS
ENLRLLKLINLAIEMNSKPFYVTGLKYFRVSLQAGLKILQASFSYFTFLTSMQRRQMSN

>DmOR33c
MVIIDSLSFYRPFWICMRLLVPTFFKDSSRPVQLYVVLLHILVTLWFPLHLLLHLLLLPSTAEFFKNLTM
SLTCVACSLKHVAHLYHLPQIVEIESLIEQLDTFIASEQEHRYYRDHVHCHARRFTRCLYISFGMIYALF
LFGVFVQVISGNWELLYPAYFPFDLESNRFLGAVALGYQVFSMLVEGFQGLGNDTYTPLTLCLLAGHVHL
WSIRMGQLGYFDDETVVNHQRLLDYIEQHKLLVRFHNLVSRTISEVQLVQLGGCGATLCIIVSYMLFFVG
DTISLVYYLVFFGVVCVQLFPSCYFASEVAEELERLPYAIFSSRWYDQSRDHRFDLLIFTQLTLGNRGWI
IKAGGLIELNLNAFFATLKMAYSLFAVVVRAKGI

>DmOR33b
MDLKPRVIRSEDIYRTYWLYWHLLGLESNFFLNRLLDLVITIFVTIWYPIHLILGLFMERSLGDVCKGLP
ITAACFFASFKFICFRFKLSEIKEIEILFKELDQRALSREECEFFNQNTRREANFIWKSFIVAYGLSNIS
AIASVLFGGGHKLLYPAWFPYDVQATELIFWLSVTYQIAGVSLAILQNLANDSYPPMTFCVVAGHVRLLA
MRLSRIGQGPEETIYLTGKQLIESIEDHRKLMKIVELLRSTMNISQLGQFISSGVNISITLVNILFFADN
NFAITYYGVYFLSMVLELFPCCYYGTLISVEMNQLTYAIYSSNWMSMNRSYSRILLIFMQLTLAEVQIKA
GGMIGIGMNAFFATVRLAYSFFTLAMSLR

>DmOR33a
MDSRRKVRSENLYKTYWLYWRLLGVEGDYPFRRLVDFTITSFITILFPVHLILGMYKKPQIQVFRSLHFT
SECLFCSYKFFCFRWKLKEIKTIEGLLQDLDSRVESEEERNYFNQNPSRVARMLSKSYLVAAISAIITAT
VAGLFSTGRNLMYLGWFPYDFQATAAIYWISFSYQAIGSSLLILENLANDSYPPITFCVVSGHVRLLIMR
LSRIGHDVKLSSSENTRKLIEGIQDHRKLMKIIRLLRSTLHLSQLGQFLSSGINISITLINILFFAENNF
AMLYYAVFFAAMLIELFPSCYYGILMTMEFDKLPYAIFSSNWLKMDKRYNRSLIILMQLTLVPVNIKAGG
IVGIDMSAFFATVRMAYSFYTLALSFRV

>DmOR94b
MESTNRLSAIQTLLVIQRWIGLLKWENEGEDGVLTWLKRIYPFVLHLPLTFTYIALMWYEAITSSDFEEA
GQVLYMSITELALVTKLLNIWYRRHEAASLIHELQHDPAFNLRNSEEIKFWQQNQRNFKRIFYWYIWGSL
FVAVMGYISVFFQEDYELPFGYYVPFEWRTRERYFYAWGYNVVAMTLCCLSNILLDTLGCYFMFHIASLF
RLLGMRLEALKNAAEEKARPELRRIFQLHTKVRRLTRECEVLVSPYVLSQVVFSAFIICFSAYRLVHMGF

KQRPGLFVTTVQFVAVMIVQIFLPCYYGNELTFHANALTNSVFGTNWLEYSVGTRKLLNCYMEFLKRPVK
VRAGVFFEIGLPIFVKTINNAYSFFALLLKISK

>DmOR94a
MDKHKDRIESMRLILQVMQLFGLWPWSLKSEEEWTFTGFVKRNYRFLLHLPITFTFIGLMWLEAFISSNL
EQAGQVLYMSITEMALVVKILSIWHYRTEAWRLMYELQHAPDYQLHNQEEVDFWRREQRFFKWFFYIYIL
ISLGVVYSGCTGVLFLEGYELPFAYYVPFEWQNERRYWFAYGYDMAGMTLTCISNITLDTLGCYFLFHIS
LLYRLLGLRLRETKNMKNDTIFGQQLRAIFIMHQRIRSLTLTCQRIVSPYILSQIILSALIICFSGYRLQ
HVGIRDNPGQFISMLQFVSVMILQIYLPCYYGNEITVYANQLTNEVYHTNWLECRPPIRKLLNAYMEHLK
KPVTIRAGNFFAVGLPIFVKTINNAYSFLALLLNVSN

>DmOR30a
MELKSMDPVEMPIFGSTLKLMKFWSYLFVHNWRRYVAMTPYIIINCTQYVDIYLSTESLDFIIRNVYLAV
LFTNTVVRGVLLCVQRFSYERFINILKSFYIELLQSDDPIINILVKETTRLSVLISRINLLMGCCTCIGF
VTYPIFGSERVLPYGMYLPTIDEYKYASPYYEIFFVIQAIMAPMGCCMYIPYTNMVVTFTLFAILMCRVL
QHKLRSLEKLKNEQVRGEIIWCIKYQLKLSGFVDSMNALNTHLHLVEFLCFGAMLCVLLFSLIIAQTIAQ
TVIVIAYMVMIFANSVVLYYVANELYFQSFDIAIAAYESNWMDFDVDTQKTLKFLIMRSQKPLAILVGGT
YPMNLKMLQSLLNAIYSFFTLLRRVYG

>DmOR19a
MDISKVDSTRALVNHWRIFRIMGIHPPGKRTFWGRHYTAYSMVWNVTFHICIWVSFSVNLLQSNSLETFC
ESLCVTMPHTLYMLKLINVRRMRGQMISSHWLLRLLDKRLGCDDERQIIMAGIERAEFIFRTIFRGLACT
VVLGIIYISASSEPTLMYPTWIPWNWRDSTSAYLATAMLHTTALMANATLVLNLSSYPGTYLILVSVHTK
ALALRVSKLGYGAPLPAVRMQAILVGYIHDHQIILRLFKSLERSLSMTCFLQFFSTACAQCTICYFLLFG
NVGIMRFMNMLFLLVILTTETLLLCYTAELPCKEGESLLTAVYSCNWLSQSVNFRRLLLLMLARCQIPMI
LVSGVIVPISMKTFTVMIKGAYTMLTLLNEIRKTSLE

>DmOR92a
MLFRKRKPKSDDEVITFDELTRFPMTFYKTIGEDLYSDRDPNVIRRYLLRFYLVLGFLNFNAYVVGEIAY
FIVHIMSTTTLLEATAVAPCIGFSFMADFKQFGLTVNRKRLVRLLDDLKEIFPLDLEAQRKYNVSFYRKH
MNRVMTLFTILCMTYTSSFSFYPAIKSTIKYYLMGSEIFERNYGFHILFPYDAETDLTVYWFSYWGLAHC
AYVAGVSYVCVDLLLIATITQLTMHFNFIANDLEAYEGGDHTDEENIKYLHNLVVYHARALDLSEEVNNI
FSFLILWNFIAASLVICFAGFQITASNVEDIVLYFIFFSASLVQVFVVCYYGDEMISSSSRIGHSAFNQN
WLPCSTKYKRILQFIIARSQKPASIRPPTFPPISFNTFMKVISMSYQFFALLRTTYYG

>DmOR19b
MDISKVDSTRALVNHWRIFRIMGIHPPGKRTFWGRHYTAYSMVWNVTFHICIWVSFSVNLLQSNSLETFC
ESLCVTMPHTLYMLKLINVRRMRGEMISSHWLLRLLDKRLGCADERQIIMAGIERAEFIFRTIFRGLACT
VVLGIIYISASSEPTLMYPTWIPWNWKDSTSAYLATAMLHTTALMANATLVLNLSSYPGTYLILVSVHTK
ALALRVSKLGYGAPLPAVRMQAILVGYIHDHQIILRLFKSLERSLSMTCFLQFFSTACAQCTICYFLLFG
NVGIMRFMNMLFLLVILTTETLLLCYTAELPCKEGESLLTAVYSCNWLSQSVNFRRLLLLMLARCQIPMI
LVSGVIVPISMKTFTVMIKGAYTMLTLLNEIRKTSLE

>DmOR24a
MLPRFLTASYPMERHYFMVPKFALSLIGFYPEQKRTVLVKLWSFFNFFILTYGCYAEAYYGIHYIPINIA
TALDALCPVASSILSLVKMVAIWWYQDELRSLIERVRFLTEQQKSKRKLGYKKRFYTLATQLTFLLLCCG
FCTSTSYSVRHLIDNILRRTHGKDWIYETPFKMMFPDLLLRLPLYPITYILVHWHGYITVVCFVGADGFF
LGFCLYFTVLLLCLQDDVCDLLEVENIEKSPSEAEEARIVREMEKLVDRHNEVAELTERLSGVMVEITLA
HFVTSSLIIGTSVVDILLFSGLGIIVYVVYTCAVGVEIFLYCLGGSHIMEACSNLARSTFSSHWYGHSVR
VQKMTLLMVARAQRVLTIKIPFFSPSLETLTSILRFTGSLIALAKSVI

>BmOR1
MLLSFKDDSRSPDIQKPQNFQYMKILRFNLKIICAWPEKQLNEIRSLGHSIHRVILPIQSVVCLACGILY
IHFHFNEIPFFILASTFITVMMNLVTCSRTALVMLFERYLVLTGRFITVMHLFNFQKNSDYAYKLCTFVN
RMSHFYTLYVLFSMFMGLGLFNLLPLYNNYVSGAFSDPYGPNVTFFHSVYFAFPFDYSHNFRGYIIMALF
NSYVSVTCSIGLVMFDLLMCLMVMHVWGHLKILSHNLINFPRPKASHVITTPNGPTNVETYTEEESKEVF
ARLRECIKHYGTVDDFANDMSETFGVILLVYYGFHQVSLCMLLLECSDLSTKAMLRYGPLTLIMIQQLIQ
ISIIFELLGSVADRIPDAVYQLPWECMDVKNRRVVYGFLRRTQNPVRFKAMGMLDVGVQTMASILKTSIS

YFVMLRTVAT

>BmOR2
MMTKVKTQGLVTDLMPCIRLLQAAGHFLFNYHADTSGMNMLLRKIYSSAHAVLIVVHYICMGINMAQYKD
EVNELTANTITVLFFAHSIIKLAFFAFNSKSFYRTLAVWNQSNSHPLFTESDARYHQISLSKMRRLLYFI
CGMTVFSVISWVTLTFFGESVRMIASKETNETLTEPAPRLPLKAWYPFKTMSGGGYVFAFIYQIYFLLFS
MALANLLDVIFCSWLIFACEQLQHLKAIMKPLMELSAALDTYRPNTAELFRVSSTDKTEKVPDAVDMDIR
GIYSTQQDFGMTLRGAGGKLQNFNAENNPNGLTAKQEMLARSAIKYWVERHKHVVRLVASIGDTYGTALL
FHMLVSTITLTLLAYQATKINGINVYAFSTIGYLVYTLGQVFHFCIFGNRLIEESSSVMEAAYSCQWYDG
SEEAKTFVQIVCQQCQKAMTISGAKFFNVSLDLFASVLGAVVTYFMVLIQLK

>BmOR3
MIFVDDAVIGIKDPREYRHLRVLRTSLRLLGAWPGHYLGEETGSKYECAPMFLLMFIKIACLYLTIVYLR
NNADVLGFFELGHVYLTIFMTFVTLSRGFSLTWNPNYHKVVKKFITEMHLLYFKDNSEYAMKTHRRVHKI
SHFYTVFLKVQMIAGLTLFNVIPMYNNYRQGNYASDRPANITYDLSIYYETFDILNTPNGYIFICVFNWF
ASYICCSFFCSFDLILSLMISTVSGHFRILIHNLLTFPLPEAITASKKFVDKHRCNGNRSEFVLEEAKLY
SPAEMWQVTDRLRQCIDYHRKLVEFTGDISEAFGPMLFVYYLFHQVSGCLLLLECSQLNTAALVRYGVLT
VVLYQQLIQLSVIVESVGTVTGRLKDAVYEVPWEYMDTSNRKTVAIFLMNVQEPLHVNALGLAKVGVQSM
AAILKTSFSYFTFLRTVSE

>BmOR4
MFKIIKNIIVENDALKQVEKPQEFQYMKWVQYHLKYIDGWPNMDMNKKNVSKIRFHKRHLLVVEQTITFL
SQMFYIVKNYGKLSFFEIGHSYITALMTIVIFSRSVVTALGRYRKIARYFVSSLHLYHYKDISEYALQTH
LLVHRLSHYYTVYLISLVVTGMLLFNITPLYNNISSGVFNSPRPENMTFQHAVYLGLPFDYTTDIKGYFV
VFILNWHLSHIAASYFCTFDLFLSLLILHLWGHLRIIILNNLKTFPKPYTNNSMYTEEENQVVLLKLQECI
RYHNFIISFTVMMSNVYDVVIIVYYLFHQVTGCLLLLQCSTLDWESLSRYGPLTLIIFQQLIQVSMIFEI
LGFLSDKLPNAVYSIPWEAMNVTNRKLVQVLLQKSQKPIQFKAMNMMSVGVQTMASIIKTSISYFIMLRT
IARD

>BmOR5
MLLYYPNTQVKEKVNNVEEFTYIKFLKSFCKIMDFWPEREEKNSKTRIFRLRYILVLQFCFTLVAGVLYL
TNSVGKQTFYDLGHTIITVLMNVVSLSRLILRCFKKYDVVGQQFINKIHLYHYRNDSEYAMKIHTVVHKI
SHNMTYIFSFCIIFGTVTFNLTPIFNNIGSDAYKNPRPDNVTLQQCVYYALPFDYTGNFKWYLLVAIFNV
QKTFFCTSLFILFELSLSLMIICLWGHLRIFIHNLNHIPAPRNSFEYTKEERQEVDDTLKKCIQHHTLII
GFVRIMSETYGLAVLIYYAFQQVVGCLLLLQCSQMELKTVTRFGFLTLVLNQQLIQISVIFELLGYMSDK
LQDAVYCVPWEYMDTSHRKMVYMMFRQSQIPLQLKAMNMLSIGVKTMVSILKTSVTYYLILKTVTTD

>BmOR6
MKEEYYLQHPRTQLFYKVLAHVSTIESTIDLTWWGYTFPKYVGWFYHLQCNVVRLFGKCVVVSQILFIIL
NYQTIDKSVFIIAITITPLGALVGIKAESAKAECYVNLMKNFMDKVHIHSIYRKNENNEFVKKKVIQIER
VSRFTAYFLVILIAINCLSWMLKPTLHNIKHFEEIMNKSMEFQYYIYFWTPLDYKYNLRDYIIIHTLCIY
LGATAVTVIVTFDIFNFIAVFHVVAHIQILKNNVKSNWSDDFNESEKKGYLVSILEYHAYIIRIFGEVQS
AFGLNVASNYLQNLIEDGLFLYQIMNGEKENVLMYGLMIIILYLGGLIFLSIVLEEIRRQNYDLCEYVYAL
PWEGMSLENQKIFVVFLQRTQPDLEFETVCGMKAGVKPAFSIVKSMFSYYVMINSRF

>BmOR8
MSLSTRCLLKDFCKYVYYAGAGNFWYEDIYKETVPYKMYVVISFFTYTVMIFLENLAALFGKLPEVEKNS
AVMFAAIHNIVLTKMFLLLYHKRSISKLNCEMAAVGENLEEASIMRRQFRKMRLGTALYFISVYLSLVAY
GVESARRTIVEGAPFYTVVTYLPDYDNTTVLASFLRIFFYITWLYMMLPMMSADCMPIAHLITMTYKFVT
LCRHFDQIREKFQINVKIMAKTEATEILKLGFIEGIKMHQKLMYLADEIHRVFGIIMALQVCESSAVAVL
LLLRLALSPHLDLTNAFMTYTFVCSLFLLLALNLWNAGELTYQASLLSNAMFYSGWYFCDFEKDWCRDIR
RLVLIGCAQAQKPLILKAFGVLDLSYETFVSVARMTYSVFAVFYKRGD

>BmOR9
MLALDDPLQNVDNVEDFKYVKWLRNHLKTVDAWPVYSKSKRKIQKRYVLPIFSAACFISQTVYLKNGIGT
LSFVVLVHSYICFLINGSCLCRGILIATERCKRLATCYLKTVHLFHHKNRSEHAMKIHVIVHRLSHYYTI
YLISLVFVGMVLFNFMPIYNNINSGAFKSPRPESVTFQHAMYLALPFDYTTNIKGYFVVFILNWYISLVT
TSHFCTFDLFISLMIIHLWGHIKILMCSLEDIEGFVLGGSFKFTIEQNRKINSILQECIRHHQFTIDFTN

EMSSTFGLVILFYYFFYQVSGCLLLLACSQMDIESLSRFGPMTFILFQQLIQLSIVFELISSLSENLPNA
VYNVPWESMDKNNRKMIQVLLLQSQKLTRFKATSMMNVGVQAMATILKTSVSYFIMLRTMYQEH

>BmOR10
MRTNAKSFLFVPSKVLTLCGVWPVEKTSIFSLIYRSIMLSSQFCFLVFNGIYIGLMWGDLKAVSDALYMF
FTQTTCCSKAIGFYFNFMKIKRIVASMDDVLFTAMSIEDQATIFSHSRTVNKLYKGVLGFTGFTLVQWTV
LSLIGSGRTLPFNEMWVPTDISKSPNYEITFVVELWMMVISAALFMSVDTITVATMMFSCAQLDIIMKKT
QQIQEIPLSPDLSSRNRSELHEKNNGILIDCIKQHQAIVRFSELCEGTFQVHSFFHLGGIVFMICVIGFR
MAGESPVSAQFWAALSYLVIILGQLYLYCWCANELTTKSEQLRDKLYLTPWYDQDVKFKRNLCIAMECMA
KALTFRAGSYIPLSRAMFVSILRSSYSYFAFLNQANEQ

>BmOR11
MDEHSHFETSLNKIKVLFKYSGMNLENTVTNTYEFLNHRWVYILNHAWTLAAVTFICIGISNGQNFIEMT
CIAPCVAMTVLAVSKSFFHYINENAVKSLLENLIELERTDFERTKSVQRTEIVATEKQLLNMVINVLYVL
NCSMILVFDMTPLIIIAIKYWTTNKFVRLLPYLDIFVFVPYKFEYWVMAYILQIWAECIVLLFIGAADCL
FFTCCTYIRIHFRLLQYDFERLTSSRRESDGLRDDEDFRETYTNLVKRHQGLIESSSILEMIYSKSTLSN
FVLSSLVICLSAFNVTVVNDVTIVMTYLIFLAMSLMQVYFLCFFDMLMSASEEVGNAVYNCSWYTEKAST
GKDLLFTITRAQKPCELTAAHFAYVNLKAFMRVSFTSASITTLPTI

>BmOR12
MTRITDVFSLNFIFWKFLGLWGKSAPSKYNMAYTVFYLFASLFVYDIFLTLNLIHTPRKLETLVRETMFY
FNHLVAVTKILMMFIMRKKILVIFDLLDCEEFKPNDENSQEIMKRKTDFYYIYWRIVAVTSNLSCFMLVI
GPLIKMLIWKIELGLPVCKFYFMSDELRNKYFVIWYIYQSFGIYNQMVNNLNLDTFNCGMLWMAVGQLQI
LKTKFVNLKLNDFENGLDLKSRDDMQIERLRKYLTHYEIILKYCATVQDILNITIFVQLGMSSIVICVGL
CGFVAMPSNTETAIFMSSYLITMTMQIFVPSWMGTQISFECGELMSAAYCCEWIPRSKLFKRSLILFVER
AKTPVRITGLKIFTLSLDTFTSIMKTTYSFFTLIRQLQVDEVN

>BmOR13
MAPKQIDCFEINWKFWKFLGIWSENKPHRYYKYYSKIFITFFVILYDVLYTINFYFVPRQLDLIIGEMLF
YLTELSVLSKVFTFIIMRHKLKIIFEILESDAFQTDTEEELKILHRAKVFIKRYWKIVALVSITANLTHI
SSPLLKNLIFKVELVLPVCSYSFLSESFLKTFEYPLYFYQIVGIHFHMLYNLNIDTYFLGLMILIIAQLD
ILNVKFRNLKSGKDHTQLNESIMGLNKNLDHYNEIERFCSLVQNIFSFTLFVQFSMASCIICVCLFSFTL
SVPVEYYIFLATYMFIMIIQIMVPCWFGSRIMDKSILLSSAIYNCDWTSNSKDFKINMRLFVERANKPLS
ITGGKMFSLSLATFTSIMNSAYSFFTLLRYIQTRE

>BmOR14
MSNYIFKPFHETYRIITFTMIAAMIYPNPATEKRRLIYIGLMLLSVIPLAFMIVTEMYEFFMASDLNNTI
RHSTVIGPFIGGFVKVALMYYKRRQANELVSEINRDHLAYNGLKGEDREIAASSIRNCQIYCELGWTLIV
MSCGLSFPVIAILLKIHSFTFKLDSTKHMIHDINNPFTDDPEDRFESPFFEIMFVYTFFSSFIYIINYVG
YDGFFGLCINHACLKMKLYCRALEDAMRSDSRRHEKIVAVIEEQRRTYEYIALIQDTFNIWLGLIYVATM
IQMCTCMYHIVQSFNIDVRYIIFVISIIHIYLPCRYAANLKCMAAETPTLIYCCGWESVSDLRIKRMMPF
MVARSQVIVEITAFNMFAFDMELFVWIMKTSYSMFTLMRS

>BmOR15
MMTLVYQTDIFKPNVFFWKMFGIWADRKSSKTYKYYSFVFLFITLIMYNSLLAINLLYTPLKIELLIREV
IFCFTEITVSTKVLMILFKRNKILDAFDLLNKNEFRGNSEESSAIIQKNNSAYKTYWKLYAILSNFAYSS
QVLGPLIVKLIWKTKLELPICNYYFLNEELRHDFFSGWYIYQSFGMYGHMMYNVNIDTFISGLLMMAVTQ
LKIIQTKLLSLKLNPRERKMDRGLMNITEVLKLNEILKHYELVLKYCSTVQSILDVAMFVQFGVASAIIC
VAMCGLIMVRSSTETLLFMVTYLFAMTLQIFVPAWMGTQLHFQSQELVFAAYNSEWIPRCQSFKRSIIIF
VERAKIPITITGLKMFPLSLATFTSIMKTAYSFFTLIRNMQALQEE

>BmOR16
MSFNSEDLYLNRAKFVMKYLGVWVPPENENFARKFYKIFMMSLQHLFLFFQIIYIVEVWGDLEAVSQASY
LLFTQACLCFKITVFQINMNKLKELLKQMNGYVFQPKNINQQNIIKVQATRIKRLLFAFMISSQLTCGMW
ALKPLFDDVGSRKFPFDMWMPVSPERSPHYHLGYSFQLVTICMSAYMYFGVDSVAFSSVIFGCAQIGVIK
DKIMSIKPLGIYRNHKTYTKISRYNRKTLIECVKHHQAVISFTELVEDTYNSYLLFQLVGSVGIICMSAL
RILVVDWRSVQFFSILCYLSVMISQLFVCCWCGHELSATSEELHTILYNCAWYDQDVKFKRDLNFMMARA
RRPILLRAGYYISLSRQSFVSILRMSYSYFAVLDQTNK

>BmOR17
MREDKMEINNSQKFYTKMIFRYLYSVGLGDWWYQHEDRSDSHRKLYCLWAVISNAYIFLNICNELLANFR
KDLTDVEKNDAIQFSFAHPLIFAKIASFFFNRKKIREVFGRLLEENRSVYSCGELEKESMKQIKRYSLAF
IGVSYMTLVMSTIDGLRAHFKEGIPIRTEVTYYPSPSNSGVIVNILRFLVEFHWWYIVSVMVAIDSLAVA
SFVFVTFKFKLLQRYFKDMGLTVRRDQSNMTDEALADKFRRDFIVGVKLHENALWCAENVQKAFGWVYSV
QVFETVALLVMCLVKLVTTNHNMIFLLANFAFMLCVIILNGSYMMPAGDVTYEASEVPTSIFLCGWELVR
QTDLRFLVVVAIQRSQVPVIMKAFGIMTLSYSNFIAVSLFKFYVQFQINLF

>BmOR18
MGDRMVTRGHFFDFNIKYLFYVGLWPSNEAKRIEKIAYKIYEYQLHVLSLIFLVTTGIGTYKNHKDIIAL
LTNLDKTLVAYNFVFKVIVFVYKREELRKLIEQIVQSGDQITEDRKALMAKLVIVLTGISTVIITAFSCL
ALFEGEMTIDAWMPFDPMKSKMNLFAASQILAATFVVPCGYRAFAMLGIVCSLILYLRDQLVDLQNKIRD
LRFATGNVEKLRDDFKLIVKKHVRLLGYSKVIEMIFKEYFFIQNMAVTAELCLNAMMVSVVGLEQKTLAA
SFLAFLSVALLNAYIYCYLGNELIVQSEGIAMAAYESSWILWPVDMQKDLLIVITAAQKPMKLSAGGMAV
LSVQTYSQTLYNGYSIFAVLNDIVN

>BmOR19
MHEFVINVQNETTKLYDQLNIILYILGLQGIWVDEIKLSRRFHVFFKVVTFILHIMCGMFAGLQFFAIFT
QNSLNSQQKSDVIVIGISNPMAYIFCINFIRNRNEIKDLFYHLAVVLKIYYNDVEIEKSMVNKIKSYLST
YVFASITILVSNGIIAFFQTINSDEPFLGIITAWPDKTDTSKTASYARIGFYLFWCIHFFRISTVFAVIV
CILISIKYQYKILCSYFESLNKIFDDETSSHEVKEAEFENAFCNGIKIHTQIIWCVRRCQIMCRTVFSAN
IMLDTFVLVILMLAMVNSENDFYGLCSQMSSVLVTVVLMAFFMWTAGDINVQASQLPDAIYGSGWYNCRG
KSSARIRSLVTISMNKAQQPILMWALGFVELSHKNFVAIIKSAYSVFSVFY

>BmOR20
MIQASKYPNSKTKELFRKIAHIAYICGLPNFWIEELNLPKSFIRVYDKIVRIFNVATYFFLGIEIAAHFT
QHHLTNKQKFDLLLYSISHPILNGYGVIVSRQVGNVKKVLLDLIVNLKVKYNDPVIEEAMIKISMTYSVS
FITNCVLSMLTYTFDALLMVYKKGVTFNVIITAWPDVEDTTTEASIGRIGFHIFWWLFVTRPFAVYVLVI
NLTTCLSHQYMNLQSYFFHLEDIFKENLSQNEKEAKYEAEYKIGVMLHANTLRCTRRCHMVWNGVMSGQI
IFNISLIVIIMAQMMNSDRTLVNTFGTVLTASAILISTGFFMWNAGDVTVQASRLATAMYCSGWQNCRGK
SSVSIRNMVMNTIAVAQRPLVLRGLGVIDLSYQSYLSIVKASYTVFSVIY

>BmOR21
MNKNMNKNHYILKTYCDKIFLVGSGNFWYQKTESRNDKTLLYKIYSCVLFFTYGFMTVLEIMAAMMGDFP
EDEKRDSVTFATSHTVVMIKFISIIKNKELLKTLNRKMMMICEAHEEQTLMDEMYRTVKINVVAYCVAVY
GSATFYVFEGLRKFYNGSHFVTIVTYYPSNDDDTLAATIVRIATTLVLLMMLLTMIISVDTYTMAYLIMY
KYKFITLRHYFKRLRENVDELVAAGKARLAAEKLAQGLVEGIKMHNELLSLSKDIDKAFGTVMALQLCQS
SGSAVSLLLQIAVTMYLLLALFLCNAGEITYQASLLSDEIFYCGWHKCNSPVLSTQRNIRDIVLIAILRA
QSPLVMKAFKMVVRSTYSVFALFYAQNK

>BmOR22
MNKNHYILKTYCDKIFLVGSGNFWHQKTESRNDKTLLYKIYSCVLFFTYGFMTVLEIMAATMGDFPEDEK
RDSVTFATSHTVVMIKFISIIKNKELLKTLNRKMMMICEAHEEQTLMDEMYRTVKINVVAYCVAVYGSAT
FYVFEGLRKFYNGSHFVTIVTYYPSNDDDTMLASIVRIATTLVLLMMLLSMIISVDTYTMAYLIMYKYKF
ITLRHYFKRLRENVDELVAAGKARLAAEKLAQGLVEGIKMHNELLSLSKDIHKAFGTVMALQLCQSSGSA
VSLLLQIALSDQLTFTMGMKIFFFLAAMYLLLALFLCNAGEITYQASLLSDEIFYCGWHKCNSPVLSTQR
NIRDIVLIAILRAQSPLVMKAFKMVELTYATFILVVRSTYSVFALFYAQNK

>BmOR23
MRAKTEFEKTIKLTKTALFLSGINIFLGEWNHWTRTFVDSIAYYLNIVGLYFVLIGEMYWLIDGTITGKS
FVELSLIVPCLTISVLATAKVHYLYHNKESLLDVVDKLREIYPDEIEETANDNDQCLNDKKETVYDNDVT
EVGIVNEANELLKFVNFLLSTVSFVVTMTFCTMPLFGMAGEFMETGKFVVLYPFAVKYPFDVYNTSFWVI
VYVNQFWATIIVCTNIFGVDTLFYALCSYIGMNFRLLSYKFEHLEIKRNDRIINEIIVLIKRHQELIELV
NKTQSLYSLSTLFNIVTSSLLICLSGFNITILSRSWSYFALLKTIYS

>BmOR24
MPEELFLDRSIKKIESYFRWMGINIRSGDNNNKKDVFKIRCIYFINFVLLNTDVLGAIFWFRSGLEQGKT

FTEVTYNAPCLTFSFLANFKMLSLIFYEKTVHELIAALQKLEIKHFLRQNCAEELKMLKDEKNFLHAVFK
GSKIVNYASILTFGCSPLVLIASNYYKTGRMDYLLPLIVLYPFDVDNITVWPIIYVRQIWSVITAVIGVC
ATDYLFYTFCVYISTQFRLLGHSIERVVPNNGLSVRTRLNGNLRMKFVENLKWHQELIRAASLLEQIYTK
STLYNFVTSSVIICLTGFNVAVVEDFAVILSFLFFLFMSLLQIILLCFFGDKLMKSSTNISDAVYNSKWY
LTEKNVGKVLLMVQIRSQRACRLTAYGFAEVNLRAFMKILSTAWSYFALLQSLYSSHE

>BmOR25
MFEKALRSANFYMRVIGIPTDIRDGNRTLMERLRNRWFYCINFLWLNTDVAGEITWFVKGLLNGSSTLIE
NTYLIPCLTLCILGNVKTFFTIKYANHIIDLVAILKDLEIKNNAARKNETEIVKERLKFLTTSNKFLLFV
IGTGIIAFGIGPLMLTASIYFSSGDMKLKLPFLIWYPFDSSDIRYWPFVYVHQVWSACIACCAVYGPDCF
YFTSCTFIHIHFIHLQNDITNVIVESSRARKNGLYRGCHQAFLELTNRHKDLIRCVNLLEIIYSKSTLVN
VVSSSLLICVTGFNVMVTFCWFAAPFASFLALGLVQTYLLCYYGDTIMCSSTEVSDAVYNSTWYGTNISQ
MRDYLFVMKRAQKPCKLTAYGFSDVNLRTFSRILSTAWSYFALLITIYRGNGQQ

>BmOR27
MPSSFFLPNLENPDYPSLGPTLKGLKYWGMWQSGGIKRILYNSIHAFATFFVITQYVELWIIRNNVELAL
RNLSVTMLSTVCVVKAGTFVCWQKYWSGIIGFVSNLEKEQLSKNDAATQAAIVKYIKYSRRVTYFYWSLV
TATVFTVILAPLVGFLSSPERELIANGTLPYPEIMSSWVPFDRSRGFGYWVTALVHTLICFYGGGVVANY
DSNAVVLMSFFAGQMKLLSINCSRLFDDGNEVISNNEAMKRIKECHYHHVFSTIFNSLMSPVLFLYVIIC
SLMLCASAVQLTTDGTSNMQRIWISEYLMALIAQLFLYCWHSNQVLYMALEDRLGGLFEACLESGRFPSK
WKTGRLVLLRKDGRPADSPAGYRPIVLLDEAGKMLERIVAARIVRHLTETAPDLSAE

>BmOR29
MFDFLQNLEDSERPLLGPNFWLINKTGLLLPKTNFGKLAYILVHEIVTFFVVTQYVELYVIRSDLDLVLT
NLKISMLSIVCIVKVNTFVFWQTSWREVLEYVNEADKFERNQTDETRGKMIETYTKYCRRLTYFYWSLVF
TTFLTTTNTPLMRYWSSPIFRENLRNGTEDFPHIFSSWMPFDKNHSPGSYCTIVWHVLLCAYGAAIMAAY
DTCIVVIMVFFGEKLNLLRERCKKMLANDLYNHAFVIGQLHDIHVQLIKQSRLFNSLLSPVMFLYILMCS
LMLCASAYQLTSATSTAQKLLMAEYLIFGIAQLFVFCWHGNDVLFKNANVSLGPYESNWWSSSPRVRADV
LLLCGQLRVRHVFTAGPFADLTLSTFIKILKGAYSYYTLLRK

>BmOR30
MSVSNLKFEALFKPTTMSLHMNRSHPSIKRNKIWLLQFISLMTLTAFCATGLITSLLFHDLKFGKYMEAS
KNGTIAMLSFTTTFKYSLLLYLQKSLNRLIAKIDMDYEIAKGLPPQEKATVLNYAKKGVIVSKFWLFTAF
AITFCFPLKAFIIMGYRFFIKNEFRLEPMFDMTYPEPIESYKTSFPVYFILFVVFFLFGCYASSLFVAFD
PLVPIFVLHACGQLDLLSLRITKLFSDTKNPRIIAKELKVIISKLQELYGFVNFIKVNFSILYEYNMKIT
TISMPLSAFQVVESLRRGEFNIEFTYFFFGCILHFVMPCYYSNLLMERSENFRFAIYSCGWENHHDKNIR
QMLLFMLTRATEPLGIATVFTNNSLDTFAEMCRQSYTIFNLMNAAWA

>BmOR33
MIYYRKCKMELNFDKIFKIAIISQKFSGTYPYTKRDKKWATHFILMHGELTIICMLFIYNIIEFDLKAAD
YSQMCRNMCLSFVYMVITLLYINMLYYQSKLKMLIETMKAEYELAKTMSEEEQNVILEYAKKGRWLCRAW
AILTTCGMAQFFLKSIVCTIYSAIQGNFRIVQYYEVICPEVIERHRNNPVIFITLYFCTFFYSLYTSALY
TSVLPLGPIFLLHGCAKLEIVRLNIKNLFDNDDYVVQERLKKTVLQMQDIYCYSHEINECFQILYEFLLK
ATSLVLPITIFAVIQALGRGQFIPEFFAFIFGAFMVGTTPCYYSNMLMEKSEDVRMTLYSCGWETRFDLN
TRKCIILMLCRALRPVSIRTIFRSVSLTTLTDVFQQAYALFNLLNAVWN

>BmOR34
MIYYRKSKMELNFDKIFRIAIISQKFSGTYPYTKRDKKWATHFILMHGELTIICMLFIYNIIEFDLKAAD
YSQMCRNMCLSFVYLVITLLYINMLYYQSKLKMLIETMKAEYEIAKTMSEEEQNVILEYAKKGRWLCRAW
AILTTCGMAQFFLKSIIVCTIYSAIQGNFRIVQYYEVIYPEVIERHRNNPVIFITMYFCTFFYSLYTSAL
YTSVLPLGPIFLLHGCAKLEIVRLNIKNLFDNDDYVVQERLKKTVLQMQEIYCYSNEINECFQVIYEFLL
KSSSLVLPITIFAVIQALGRGQFIPEFFAFIFGAFVVGTTPCYYSNMLMEKSEDVCMTLYSCGWETRFDL
NTRKCIILMLCRALRPVSIRTIFRSVSLTTLTGVFQQAYALFNLLNAVWN

>BmOR35
MKLWQSIREFGLEYCDLPTTLQNVASLLRAITLNIDSRHTARIPFICYVMTVVITLSYFYVFLVSMAWFV
FVRSAETRDYLAAMVVLSLGISSEIGTLKFFYTFIYIKKVQRIVREYLECDHMVVPESRFADNVLKTMRN

VKKRAILYWVVVIGNGVVYVTKPLFMSGRHHMEDRYIVYGLEPMFESPNYEVAYFLMMFGLCFICYPPAN
VTVFLIVVVGYTEAQMIALGEEMLRIWEDAVAHYNNKYHTVGALTNSSEKNKIINQYVKFRLTEIIKMHT
TNIQLLRQVEFVFRSAIAMGYVFLVLGLIAELLGGLENTYLQIPFALIQVLVDCYTGQKVMDASSLFEQA
VYDCKWENFDKSNMKTVLLILQNSQKSMRLSVGGITVLGFSCMMSVMKSIYSAYATLRTTMS

>BmOR37
MELGCSRHLKLPCSLHPIGISKHGNTLSELLIYFPAIPKITYAILAVLLTVYYYIYLCSITWFVFVRCPQ
TGDLAAASIVFSLGVSSEIGAIKLFIIAKLRDITGEYLQCEADMAPGRLRARVGRSLRTVRRRAFVYWLV
LVVNAFAYDLMPAFLPGRHLSEDVFVIYGFEPMFESPNFEIASTLMGVSVVFICYTAGSISAFLIVIVGY
SEATMLALSDEISCVWDDACASECQQPNDFIRARLGKIVAIHTKQIRLIREVEVVFRGALAGGFACVAFG
LIAALLGGLENTFLQLPFCVIQISVDCFVGQRLRDANVAFETAVYNCKWEYFDKSNMKTVLLILQNSQKT
MGLTAGGVAALDFTSLMTIFKSVYSGVHHSQTDD

>BmOR38
MNLSQSVNEQANEYVKMRLERISKIHSPMLPFEDIQDFRELCCIPLAVYAVTGSITASYVYAFLISLLWF
LFARCTDPEDFQVAMVVFSLGISSEIGSTKFFNSIIYIKELRKLFKDYLLYDATCPAQGRLRLHLLTTLR
YVKRRAIIYWLVIIGNGFIFAIKPLLVEGRHLAQDDLVLIGLEPMRQSPNYEIAYAIMTMGVCFICYPPA
HVTMFLIIIVGYTEAQMLALSEELKHLWNDAIEHYEKHSRTEREADAAMKSKILNSFVNFRLVQIIKSHS
TNVNLIGRVENVFRGSLAVGYVFLIVGLIAELLGGLENTYLQVPFALIQVAIDCFIGQRVNDANIDFEKA
VYDCKWENFDKRNMKIVLLLLQNAQKTVSLSAGGIAKLNFSCFMSVIKSIYSAYTTLRTTMK

>BmOR39
MLWSVFSYFTRADDVLAGIVIFSLGVSSEIGLVKLCFMYANIDKIQKITEGYLKSDAASARNSRFSKNIL
HTMQSVKKRGVIFWLVIISNGVVYLVKPIVTPGRHFMEDQFIILGLEPKYETPNYEIGFFMMAVGVCVTC
YLPANITAYLITVAGYSEAQFLALGHELANLWPDAQLHCRAMNLSQSVNEQANEYVKMRLRELVKIHSTN
VNLLRDIEGAFRGAIAVEFLLLIVGLIAELLGGLENTYMQVPFALIQVSVDCLTGQRVMDANLALERAVY
DCRWEEFDASNRRVVLLLLQNAQKVATLSAGGIATLNFSCLMAVIKSIYSAYTTLRTTMK

>BmOR40
MTGAGAGTFRTGAGPGRGDGVARRGESGETTTLGRDAFAALGCFGAADGSTARARFFPRVTVLNPSEVPG
SGLAADSNSISDSESEPELDAAQDAIDAGAGVGGDIGESRARTVFGIQGHDASDSALRMHNNVAIYAKTT
MSGNSQLTFATAATIFLKNASGPNGVAIGTDYAICVVSLSLFFCYRFTELVEDTYNSYLLFQLVGSVGII
CMSALRILVVDWRSVQFFSILCYLSVMISQLFVCCWCGHELSATSEELHTILYNCAWYDKDVKFKRDLIF
MMARARRPILLRAGYYIGLSRQSFVSVSIPRIRFNAILVI

>BmOR41
MMGNSTDLFLDRTKRILNFFAMWRSFEKPIPLKVYMAFIMTTQYLFLIFEIIYIVNVWGDMAEVSEASIL
LFTQASVCYKMTAFISKTNNFVILLGLIESEIFSAQTELHEKILILKARKIKRLCMFFLVNAVTTCSLWA
VIPLLDISSKMLPFKIWMPVSTGESPHYELGYLYQMITIYISAXLFISVDSVPLSMIMFGCAQLEIIMDK
IGKVKSWSLDQQPMQKQEVLNSNYELLVECVRRYQSVVRFIELTEKTYHANIFFQLSGSVFIICNIGFRI
AIVDSNSLQFYSMLTYLVTMLSQLFQYCWCGHELTIRGEELRETLYQSPWHEQDIRFRKVLIITMERMKR
PIIFKAGHYIPLSRPTFVAILRCSYSYFAVLNRVRNE

>BmOR42/43?
MDIPKFEELLKQIKMNFWLMGIPFDNPKIQIRYYVLLLPLSLMLIEEIAFFGSRMSSENFLELTQLAPCI
CIGVLSVLKILALTAKRQKIYELTQNLECLHKIILNDTRKTELVRKNLVLIKFITKYFFVLNAVLIFVYN
FSSPVIIAYNYIVSNEVQFVLPYAVLLPFKTDSWIPWLIVYVYSIFCGFTCVLYYATVDVLYCVMTSLVC
NNFSLISFKLQKVNRNTAHLLKEVVKEQQYVLKLAEDLENIFTAPNLFNVLIGSVEICALGFNLMIGDLT
QIPGCILFLSSVLLQILIMSVFGENLISESSRIAEAAFLCKWYEMDQKSKKTILTIMIRSHKPKKLTAYK
FSVISYGSFSKIISTSWSYFTILRTMYTPPGTKFQDDL

>BmOR44
MYTYFKVLVFWLNKDKVISLQKILHCKEFKPKEPEHKEIIRKSIRKARFVMTSYATMCVGAVSVGIILPL
TENFDILPTNVEYPFFDVYKNPTYAYLYLHHIYYKPATCIIDGVMDTILAAFVASAIGQIEILAFNLRNF
DVLAERRRKRAISGNKYIGKYTNLYFTKRILKECILLHNSIIRYVSVIESAFSLASALQFMLSVMVLCLI
GIQFLSIENPTSHPMQMVWMAIYLTCMLIEVFILCWFGNELIWKSNDLRQAAFDGPWRNLNRKTCMFIII
FMERCKRPMRLSAGKIFTLSLDTYTVLINWAYKAFAVMRNMKK

>BmOR45
MKVLDNVNHAVKVTMNCCRLYGLFVSDDLTKRQLIIMRAFSLMLYLFFVGFFITTQSALIITMWGDLNLM
TNVGLVLGTHLTLSAKVFTLHYKEKEITNVIYKNEVRLRAETREQGKYIISEMNRETTLFMRLFIPFGMG
TVTAWLLCTPKGELYTPAWYPCNTTKSPAHEIILAHQGIAVILTATLEIAIVLLMTSIVAVCRCRLKLVG
LSFETICDDLPSNIMNKLTADEQVIVAKRVRENVIEHQAVLECINDIQDCFSSAMLVHIAISTMIICATA
YQLAVEKSLDLTQRMTMASFLGGMSTEIFLFCYQGGHLSIDSMEVATAVYSCPWYTFPTSLKRSLLVIMI
RAQQPALLTAGGFAPLLLDTFVSIMKASYSFFTVLQNASE

>BmOR46
MAFFIRNKMLGLTITLNTLSWAGLIMRDQYTKTQRIIVRVYGWLVFLYLFVAATYVQIADLIDIWGDLDL
MAETSLLLFMELAVISKILTLIFKYDKIMEIINGTEDILCSENRLEGQKIIASIDKETTRFFQYYTSSVI
FTTFFWFLGEHSSTFFIRAKYPFNELKSPGYEFALIHQCMMMVFTGYFEFNINIFFASVVAGCRCRLKLV
ALSLRNICINIPVNKKNLITPEEEKLITERLHCAISQHKYALDAAEDVKHCLSKVLLVQLTVSIVIICTT
AYQMAVNKSTDTIQKLSMAGYLLGASFEVFLFCFQGQSLSNASEDIADAVYECPWYTLTQPLKRTLLIIM
MRAQSPAILTAGGFVTLDITEYMAVLTGHGGFGDFLHRTGAEPMAECHHCGCDLDTVQHTLLVCPAWKGW
RRDLVVKIGNDLSSVLWHRCSAATSRGRRCLTSASAPSRRRRRGA

>BmOR47
MKLVFDNFIFALKVTLNWCRYFGIFIPDELTGRRQKLLVQAYSVFMFMLFIGFFIITQIILFILVWGDLS
LMTDVGLVLGTNLALSAKIAVFFFKREELASILKKNDDTLRFETREEGKKIISEIDRETNAFMKVFFCFG
VGTVIAWFLSTPKGELHIATWYPCDTKRSPAYEIIMIHQLAITADLLMLSMIAVCRCRVKLVGLYLQTIC
DDLPCNVKNKLTSDEEVIVAKRIREYVIEHQAILDCISELQNHFSPALLVQLLTSVVIICVTAYQLAVEK
SSDLLRKFTMASFLFAMSTEMFTFGYQGGHLSHDSMEVATAAYSCPWYTFPTSLKRSLLVIMIRAQQPAL
LTAGGFTTLSLETFVTIMKASYSFFTVLQEATD

>gi|152963629|tpg|DAA06004.1| TPA_exp: odorant receptor 48 [Bombyx
mori]
AAACVQIADIIDIWGDINLMAETALLLFMEFAVISKILTLLLRYDRIMEIINGTEEILYFENGLEGQRII
ASVDKETTRFLQFNSAFVVLSTTFWFTGEHSSTFFIRAKYPFNELKSPGYEFALIHQCMMMVFTGYTVFN
INIFFASVVAGCRCRLKLVALSIRNICINIPVNKKNLITPEEEKIVKERLHCAISQHKCALNAAKDIKNC
ISEFLLVQFTVSIIIICTTAYQLAVVCLFQNKAIGNIQKTSMFGYILGASLEVFLFCFQGEFLRNAVRDC
EEIADAAYECPWYTLTQPLKRTLLIIMMRAQSPVILTAGGFIDLSIREFMGILKASYSFFTVLQQVSE

>gi|290563354|ref|NP_001166614.1| olfactory receptor 49 [Bombyx mori]
MLTCFATIFSAVNQTGYIVLFINLLAHELGHFYVITDVLNGIFEKNDADRDPVFIDRKLKFCAKHYQYLL
KFHNEIKNLYKIIFGAHFLMMTIVLVTTLQTMNSWDIRNTVLTAVTGIMPLFIYCFGGELLITAGMDMST
AIYQCGWEKMGVKQAKVVSVILCLSQRPLCLTAANVFVMNRETFGGIAQVVYKIYAVFN

>BmOR50a
PIFAAKTLKDKQHLIQNKKEVTRFARLLLTYVTVGGFIWPMSFCFRRIKDPNTVVPFYVPFTPDNWTKLI
NEVTDIFNPCLTFQFFTSSVAICMVIYKLSDTYIVSLEFVFLLNFIFVLLTQMFIYCYYGNVVSYESKYI
NTSLYLSDWSSASPGVRKMFLIVMPRWTRPLVVRIARVVPLSLDSFVSVRKYKCSIEHFIYSHVKQTRAR
DIFSGASNKP

>BmOR50b
MEGTSEGDEPFIYRLNDNGTGPNLVVKILYCPCWYTAHLMPFKLHYCFTAEIVRLMIVPLVDSQDVLPPV
ITQIVISIFITRCYSFIVEVNREHLLMQETLLKPARRDPKIANYNMVRNDRLSARGGGTVIYYRRALHCV
PLDPPALANIEASVCR

>BmOR51
MDCTIVAFYSQAKTQIKMLRYDLEQLGKIDNIETKFTENIFERSSHIWKALKDEKIKIHSKLVFCVEHYR
QIVWFVKEVESIFGEAMTVQFFVMAWVICMTVYKIVGLSIYSAEFVSMGVYLGCMLAQLFIYCYYGTQLK
VESESVNTSLYCSNWLSSLPKVRRQMLIMMQYCSKPLTPRTAYVIPMSLETYISVLKSSYSLFTLLNQKH

>BmOR53
MALKKMLALTKGLEDPTHPLLGPTLKALSVFGLWQTGSQKSTVIYNTFHFLTFLFVITEYIDLYTVRKEL
SKMLNNLSVTVLSTICMIKTLSYVCRQSHLKVLVREISELELELMKTTDKNIVKRLRQYTVYTRAVTYVY
WFLVVGINVVLLTSPLLKYASSEIYRSEIKNGTEPPPLILCSWFPFDSARMPGYFWATMVHIIMSIQGCG

VVATYDMNAVAVMSYLKGQTSILKDKCKAIFDETASSRDVLNRIRDCHRHHNILLRHYYMFNSLLSPIMF
VYMLICSFTICCSIIQLDSSETTISQRIWIIQYSIGQISQLFLYCWHSNEFAAKVKKKHFPLFPINLF

>BmOR54
MGLNTIKEFFVNVKRRFQDVSIDSLLWIVNIVPSLAGFSIRSDRVSAPFWIVHWSLLVYVYAVGNAVYQW
KFANEAIDYITSFINVSLLILIGNNSWWFLANRRLLKSVLHKIEVNDELSRRSEQSRLKHKKLLKIIKRI
VLVFYMSNYVNASFIYLPNRVDVLNNYAMTPCVGMEPLTVSPNRELCLTILCMQEFSIMTVVLNFQALLL
CFIAHTAVMFQILADEIMALNNYENLEEHQAYVKEMLPIFVKRHSLTLSAVDNYKSLYSVPLGVNFGSNA
LTILLILYLPVLEWFKFIPIFVFCFMLFFLYCFLCQKLVNASEAFETAIYCCGWENFALREMKMIYVMLH
QAQKPVELLAADIVPVNMNTFATTLQAMYKFVTVVKF

>BmOR55
MCFLKIKQQIIDIQKHFKDYSLNGSLWIVNLLPRLMGFNLRADKVGVFFWTIYILLLVYVFGIGIFVYLW
KHVDTMSGLMKSYLNLSLILVIVNNSCWFLSKRSLLNKVLKKIHLIEDLSCESEHALAKYRRVFKIVTHL
LLASYVLFYFTEIYFMFLFRNYDLLEDYSLAPCVGLEPLSSSPNSEICLIIVLIHEFISTTVMMSFAALF
LVLIAHTAVMFLVLAEDMTKLTDLINLADHRKMIRESLRSLIHRHSLLLQIVYELRLLYSVPLGINFISN
AMSILVLLCLPIHEWPSFLHIIGYCFFAFFLYCFLGQNVINASEKFIDAIYCCGWEHFGVAEKKLVHVML
RQAQKPVEIIALGMISVNMNTYVEALQLIYKFVTVLKI

>BmOR56
MKLLEKLEDPDRPLLGPNVKALKFWGLLLPESRSKKYFYLFMHFAVTVFTATEYIDVWFVKSDLALLLNN
LKITMLATVSVLKVTTFLLWQNAWRDLIGYVSRADLEQRATSDSRKLALINGFTGYCRKITYYYWFLMYT
TVAIVTVQPIFKFFSSAAYRLDVQSGNGTYLQVVSSWIPWDKNTLPGYLLASIYQTYAAIYGGGWITSFD
TNAIVIMVFFRAELELLRIDCAALFDDEKSFGDMAFMRRLKECHRRHTELVKHSRLFDSCLSPIMLLYMF
VCSVMLCVTAYQITIETNPMERFLMTEYLVFGVAQLFMYCWHSNDVLYASQDLSRGPYESAWWSRDVKYR
KNLYILVAQFNKVIVFSAGPFTKLTVATFIRILKGAYSYYTLLSQSQMNKT

>BmOR57
MPSLLKTESLALTLTLNTLSWAGLILRDDYTKTQRIIMKVYGGLVFLYLFVFTAYVQIADLVVIWGNIDF
MTETSLILFMQLAVSAKVLTLMLKSKKIMEVTNEADAILNSEKKVEGQRIIASIDKNTTLFLKYYGFFVA
FTIICWFMGENTSTFFIRSKYPFNELKSPGREFAFVHQCIVVIFTGSFDFNVDIIIISLVAVCRCRLKLV
ALSLRNLCLDIPMNKRNLITSDEEKVITERLRNIISQHKRALDAAEAIKHYLSGALLVQLMVSIVVICTT
AYQLAVKKSTTMQSLTMAGYLFGTSLEVFLFCYQGEFLRESSEEIADAAYECPWYTLTRPLKKTLLIIMT
RAQRPATLTAGGFVTLDITEYMAIMKASYSFFTVLQQVSE

>BmOR58
MKLVFDNFIFALKVTLNWCRYFGIFIPDELTGRRQKLLVQAYSVFMFMLFIGFFIITQIILFILVWGDLS
LMTDVGLVLGTNLALSAKIAVFFFKREELASILKKNDDTLRFETREEGKKIISEYPCDTKRSPAYEIIMI
HQTIAVAVIASLAITADLLMLSMIAVCRCRVKLVGLYLQTICDDLPCNVKNKLTSDEEVIVAKRIREYVI
EHQAVLDCISELQNHFSPALLVQLLTSVVIICVTAYQLAVEKSSDMLRKFTMASFLFGMSTEMFMFGYQG
GHLSHDSMEVATAAYSCPWYTFPTSLKRSLLVIMIRAQQPALLTAGGFTTLSLETFVTVS

>BmOR59
MDTNPSAAGDSVAPHLRRLRQVGFCQLDPTSQSRRPILALMHRVYHRLVLAATVLYIFEQLTYAYQARND
MERLSRVLFLMLCHLTCIAKQFVFHSDADKINQLVVGLDDALCNQPVETHRLLLLETSRRAARLLMLYSG
CAVSTCILWAVFPLLDQLRGRTVEFAFWIPIDYRHNAFQFAVVLAYAFYSTSLVAVANTTMDAFIATVLY
QCTTQLRILRMNFESLPERAYALSRKTRQDYHTVTHELLVDCLLHYKKITETCNLLEQIFGKAILVQFGV
GGWILCMAAYQIVDMEILSIEFASTALFMGCILTELFLYCYYGNEVTVQSGLVSESVYAMSWLSLCPRER
RALVVVLERARRPLRPAAGRVVPLTLNTYLKILKSSYSFYAVLRQTK

>BmOR60
MVRPCRYFAIHFILLRFLGLGWWHHPHENETRNYPGLYLYYSILTQLVWVVGLVGLETIDPFVGEKDMDR
FMFSLSFVITHDLTLIKLYIFYFRNVEIQDIVRTIEIDLYRYYQNDDKIRATIRISRIFTAAFLFFGWVT
IGNANIYGIVQDLRWKDIVKNLNETTSKPLRTLPQPIFIPWPYQEDKHYILTFILETMGLLWTGHIVMTI
DTFIASVILHMSTQFAILREAIVTAYDRTMIALSEGALQSGVLCENSNGNEENNQIFLESFYSKEHIESV
LESTLLSCIRQHQLLIGCVEKFSKTYSYGFMTQLLSSMAGICVVMVQVSQGASSFKSVRLVTSLAFFFAM
VIQLAIQCFTGNELTIQAERIADAVMESKWEKMPVRLRRLLLVTMMRAQRPLHLTAAGFAYIDNTCFLSI
LKAAYSYYAVLSQKQG

>BmOR61
MARITDVFRLNFIFWKFLGIWGKSAPSKYNMAYTALYLSASLFVYDIFLTLNLIHTPRKLETLLRETMFY
FNHLVAMTKILKMFIRRKKILVIFDLLDCEEFKPSDEDSQEIMKRKNEFYYIYWRIVAVTSNLSCFMQVV
GPLIKMLIWKSELGLPVCKYYFMSDEFRNKYFVIWYIYQSFGIYNQMVNNLNLDTFNCGMLWMAVGQLQI
LKTKFVNFKLNDIENSLDLKTRDDMQTERLRKYLTHYEIILKYCATVQDILNITIFVQLGMSSIVICVGL
CGFVAMPSNTETAIFMSSYLITMTMQIFVPSWMGTQISFECGELMSAAYCCEWIPRSKLFKRSLILFVER
AKTPVRITGLKIFTLSLDTFTSIMKTTYSFFTLIRQLQVDEVN

>BmOR62
QNVILEYAKKGRWLCRAWAILTTCGMAQFFLKSIIVCTIYSAIQGNFRIVQYYEVIYPEVIERHRNNPVI
FITMYFCTFFYSLYTSALYTSVLPLGPIFLLHGCAKLEIVRLNIKNLFDNDDYVVQERLKKTVLQMQEIY
CYSNEINECFQVIYEFLLKSSSLVLPITIFAVIQVSSLHICFIFLIPNTQWNVCSQNKHFLGSLEMSCRNC
HSK

>gi|238623765|dbj|BAH66356.1| olfactory receptor 62[Bombyx mori]
MELNFDKIFRIAIISQKFSGTYPYTKRDKKWATHFILMHGELTIICMLFIYNIIEFDLKAVDYSQMCRNM
CLSFVYLVITLLYINMLYYQSKLKM

>BmOR63
MKLWIRNANFTISLSLTLLRCLGFWSPDGLAGNKRLLYNCYSFVFFMFLLGIYILIQVVDMIKIWGDLPL
MTGTAFLLFTNFAHATKVINIVIRKNRIQRVIQQANAVLMGVQSEEARRIVKSCDFETSIQLCLYFLLTF
VTTVGWATSAEKHQLPLRAWYPYDTSKSPAYELTYIHQVAALLIAAYINVAKDSLVSSLIAQCRCRLRLV
GLALASLGQDLKIDYQSQLSPAQENILNLRLKTCVLEHQTVLAAVTELQACFSKPTFAQFTVSLIIICVT
AFQLVSQTGNLVRLLSMGTYLMNMIFQVFIYCYQGNKLSVESSEIAGSVYFSPWYLGSVKLRRALLIVMV
RSRRVAKLTAGGFTTLSLASFMAIIKASYSLFTLLQQVKQKK

>BmOR64
MGVSNGRGTVKPFLYPLVDELDYNLIVGVHLPFEYKTPSRYPLAYITVVIAFIYVSYFVMVTDLIMQAHL
LHLLCQFNVLADCFENMLNDCVKGFEGPLVSLHEYIHPLIDEFEYNLMVGLRLPFSFDTPLRYLFTYVIV
LIAFNYTAHYVMVTDLIMQSYLIPLICQYAVLADCFENILIDCSNDYGDHARRNDIVYSRSMELRAILSR
PMLGQLASSGLLICFVGYQATTSISVNIVKCLMSLFYLGYNMFTLFVVCRWCEEITNKSLNIGNAVYCSG
WESGMTVVPTVRSTILLVILRANKPIVFTAGGMYNLSLTSYTSLVKGSYSALTFLLRIQHE

>BmOR65
MRLGFEVSISEYLYRNIFYIYTLFHILLHFYYILHMIKLDLEAIFDDIDESVALLPHRDTRRIEVQKILN
GRMKRVVTWHISVFKAVEAVSSIYGPPLAYQVMFTSIAICLIAIQITQKLENGILDIRFTMLGVAACLQM
WIPCYLGTLLRNKAFGVGEACWNSGWHQTPLGRMIRQDIIIVLLRAQQPVTIKFPGLQSIQLETFSSVI
NLYGYYYFLLLRWVDELTAHLVLSGYWSP

>BmOR66
MRFGLKVYIYTLFHILLHFYYILHMIKFDLEAIFDDIDESVALLPHRDTRRIEVQKILNGRMKRIVTWHI
SVFKAVEAVSSIYGPPLAYQVMFTSIAICLIAIQITQKLENGILDIRFTMLGVAACLQMWIPCYLGTLLR
NKAFGVGEACWNSGWHQTPLGRMIRQDIIIVLLRAQQPVTIKFPGLQSIQLETFSSVIFNLYGYYYYYCL
DG

>BmOR67
MRFGLKGGAAVVTILETLELISQGGFIETIQVTFGGQLSSMLFISACIICSTAVQILAIESPLDNLTTVG
WILVYLSLCILILFVDCYFGNTITVKCAYLPTAVFSIPWLDQPKNIQVSTLLFMAKTQQPVQLIAAKLVP
VSLTTFTQVSYCPPLDLKCLQGGVIAHLAKD

>BmOR68
MFTIDFHDERITSFNKNQTRKIIICVITGGRTSCESARVGTTTLPISASEDVCMTLYSCGWETRFDLNTR
KCIILMLCRALRPVSIRTIFRSVSLTTLTGVFQQAYALFNLLNAVWN

II. Predicted amino acid sequences of Gustatory Receptor queries

>BmGr1N
DIYGPEITDKDGALLDKHDSFYLNTKSLLVLFQIMGVMPIMRVPKSAQTTRRTTYNWISKATLWAYLVWGL
ECIIVVKVGQERLANFQIGSNKRFDEVIYNIIFLSILIPHFLLPIASWRHGPQVAIFKNMWTHYQLKYLKI
TGKPIVFPNLYILTWGLCIFSWVLSFAVVLSQHYLQDDFELWHSFAYYHIIAMLDGFCSLWYINCNAFGTA
SRGLAINLHKALEAEHPALKLAQYRHLWVDLSHMMQQLGRAYSNMYGIYCMVIFFTTTISLYGALSEILEH
GLSYKEMGLFVIVAYCMTLLFIICNEAYHASRKVGHEFQDRLLNVNLGAIDRSTQREVEMFLVAIAKNPPI
MNLDGFTNINRELFTANISFMSTYLIVLMQFKLTLLRQGARKTVTAIVRAIFNTTITDNGAGGSDEDQE

>BmGr2NJ
EQEQRDLLSSQDGDTCEIHDQFYRDHKLLLVLFRALAVMPITRSRPGTITFSWKSTATIYAVCFYIAATAV
VLIVGYERIQILQSIKRFDDYIYAILFIVFLVPHFWIPFVGWGVAHQVAIYKTNWGKFQVRYYRVTGENLK
FPNLKTLIVIISVGCLLLAVCFLLSLCALLDGFLLKHTSAYYHIITMINMNCALWYINCKAIKIASQSLSE
CFQRDVDIECSAQLIARYRYLWLNLSELLQSLGNAYARTYSTYCLFMFANITIAVYGALSEIVDHGFGFTF
KEVGLFVDAAYCSTLLFVFADCSHKSTLKVAAGVQDTLLSIDVLAVDRPTQKEIDHFIQAIEMNPAFVSLK
GYAHVNRELLTSAISMITIYLIVLLQFKISLPKEPHGTGQ

>BmGr3F
MSFEIKNNFFRTSVPIPNGFPVQTEAKSKNKPIFLDVSPAPTPKVNSPNAIIPMKNNLIDPFINKDIIYEN
IKPVFMVLRIMGVLPLTRTTSGVNEFHFISPAMVYSLTVFIILVSYISYLSLHKVQIVRNSEGKFEEAVIE
YLFTVYLFPLTVVPILWYETRKIANVLNGWVQFEVTYKQLSNRILPVKLYKKSLLIAIIIPILSTTSVIVT
HVTMVHFKTSQIIPYVFLEILTYMLGGYWYLLCEILSLCANVLADDFQQALRHVGPAGKVAKYRALWLRLS
KLARNTGVANCYTFTFVNLYLFLIITLSIYGLLSKISEGFGTKDIGLALTALCSVFLLFFICDEAHYASHN
VRTNFQKKLLMVELSWMNTDAQTEVNMFLRATEMNPSQISLGGFFDVNRTLFKSLLATMVTYLVVLLQFQI
SIPDATQPEIPTNIDDHVQNITDTTTEASSPISTLMSAFAKRKND

>BmGr4S
MDKDKFQEFLPTMSRIFSMTRYFGVSTCKPSIAFGWTVILLLMLLAIEVGAIWKIVRLLGGWAVHSTDSRG
FTARLSGCIFYGNALLSLILSIKFVSSWEQLSERWSRTETDPGLRLPSDSRIKRRTVLVSAFVMTCACVEH
MLSMMSATGFDCPPEEYTERYILSSHGFLVQNDEYNLWLAIPIFIMSKLATALWNFQDLIIILISMGFTSR
YNRLNTYVHRVVMLERNLKEGAQVSSENYMRFQIWRRIRQAYVRQAALVRLVDDQLGALVLLSNVNNLYFI
CLQLFLGINSKDRGSFINRLYYFISLGWLMFRACGVVLAAADVYIHSKKALISLYLCPELAYNLEIKRLKY
QLKNDEVALTGMGLFSLNRELLLEVAAAVLKYELVLVQYDK

>HvCr5N
AYLDKVFLWSCLYGVFGSKRFISLIWSTLILGSLVIIEVLAIWKVIRALAGVARDMSGHRSVTARLAGTIF
YSISILSLVLVSKLYYNWRTNIAGVWGKVERSVGVKIPVDRTLKCRMTFVAGLMTFFSIFEHAMSILSSVG
LDCPPSLILKRYVLVSHGFIFMGQDYSEWFAMPLVIISTIATLLWNFQDQVIVLISMGLTSRYRRLNECLA
KVCELEKQHKDSDKKIEAVKVYTWRKIREAYVKQAMLVRKIDVALGGIVILSCSCNFYFICLQMFLGITQG
LSSDLLSLIYYVISLAWLCTRVISVVLAASSVNTHSKLALNHLYNYETHCYNVEVERLQDQLTKDYIALSG
MGFFYLNKTILLQMAGAIVTYELVLIQFDDQGNDALNATKI

>BmGr5NJF
YFAFYLSTGCNTFIFLRVASKWPTLIKHVYETQLDSYIDVKVKNKCFAAYIIFFSMSMTEHMLSLLSKFVI
TMDCLPKGSDLFESYIIRNFPWLFEFDVPYYLPIGVILQFLTLVSTINWSYSDLFIVCMSIYLTSILKQIN
KKIEMAGNSNHLPIPFWRTLREDYTRATRLVRSFDDTISSVIFLSFASNLFFICLQLYNILSNGVTSKYNL
LKEMCPNYPSGPLGGYEQIMYLLFSLSFLLGRSLVVSLVAAKVHSASMVPASALYNIPRNMYCSEIQRFLD
QVHGDKVALSGLRFFYVTRSLVLSVAGTIVTYELVLLQFSNED

>BmGr6P
MLLRNYKQNLSFWTSAKKSKIHKIQSQETVTFQGSLKLVLFIGQLFSLFPVCGLLSNDANKVXFVPISWKC
GYSMLSMIGQLFIIVMCILYVAHFETTLNGTTPIIFYGVTFISMIAFIRASRRWPELIQHISKSEELDPSF
DFRLKKKCNITLLLVLVLAILEHIFSIRSAYSASQICYPHTGFYEGFVRYLYPWVFDFLPYSEELGMVTQF
LNIQSHFIWNFTDLFVICMSYYLTSRLDLVNKKLLPAQGKYLPEIFWRTTRETYCRATKLVRKVDEIINGI
LFISFANNLFFVCVQLFNTFDDSVDMVGLCYNYSERRTKPVGREPVIYLLFSLGFLISRSITVSLIASQVN
LASTVPAPILYDVPSAVYCVEVQRFLEQVNGDNVALTGLQFFSVTRGLLLSVAGTIVTYELVMVQFNQAPA
SDSFTEKLVENNISTIETFYNYS

>HvCr1
MGPTSLKRNMFFWIPVKKNKVDVAKPKVKNITTFQDALRATLIIGQVFSLLPFVGVFTNVASNVKFIKTSW
KCGYSLLSLIGQMFMAVLCVNKLAKSNVSLNGTSPVIFYVTTCVTMMLFFQVARRWPALVQHISKAEDMDP
NFDCSLTRKCNITCAVVLILALLEHILSLLSAFAGASACYTGMDTYQGFVTHFYPWVFNYLPYSIVLGVIT
QFLHFQSTFIWNFSDLFVICMSYYLTSRLEQVNRKLLAAQGKYLPEIFWRATREDYCRVTQIVRKVDEVIS
GVVFISFANNLFFICLQLFNTLEDGLKGTGECTQLNSQSKLKKIVVSKSGPLGGHEAAAYFLFSLVYLLSR
SVAVSLIASQVNSASSVPAPVLYDVPSPVYCVEVQRFLDQVNGDKVALSGLQFFSVTRGLLLTVAGTIVTY
ELVMFQFNSSTPSLNITSPTSATHIITTLAT

>BmGr7
MVLEAHTQIQYCTAKANYCEFHAGLRHLMRLARWAGFFPVQGLSQTNPDDVRFEFRSLYALYHAITVIGQT
VMTFLAFYSFVDSNVSLSVVSNFLFYFTNYVTLVLLWRLSKNWSALISKTLEFEQSVTEIRTTRNLVSRTN
TLTYVVLIFAMVEHALSKVFNIRSVMCCLGETSLNHTVINNYFKFKWKFVFDYFSTSTTYSYFVGFIAEFL
CMQATFLWSFTDVLIMCFSIYLSSFFEDFNSTVSSFMKKASKTVPWSTLRVQYSQIVLIVKQMDEQLDYFV
LISYFTNLFFICFQLYNSLNRIYDANDVCNENMDIIATASVTYLTYYVFSFLFLVTRALLLSIMAANVHSC
AQVPQLALYEVPTADYSLDVQRFQLQLRYTTVGLSGVCFNVTRGMILRVIGTIVTYELVLIQLTKKNLDND
TSIRDYYLPKHLI

>BmGr8R
MAPRSVRSMVGTSKKDMLKGGFYETVRIPLYIYRLIGILPISGLWHRSSKYNRFSLKSFYTIIYAPTIVMQ
TFLLLVHIYDLFAFFFGHQRLGRLIYHMNFYTITILIFMGSRKWKNVIKEIETIELTLPRLRNSKKALALT
KSFVFAFFVFSLAEVVLILQFTLRLTKQRHVLPGDSGLYLRSYFVYIFPYLYDHFPFSYVMGFIVQIIKVQ
GIITLNMVNCSVVILSIYLTNRLKHYNRIVFAKGSKTNNTRLKWVELNLLYTRISNLVKIIDKNLNPFVFI
SFTANLSYICAQLFYILNKLTSSRTVKITSFLEDKRCDWETVLYISISFALVVLKVLLVSIIAAEVHTTSR
EPLRLLYTLPTAEYTIETQRLMTQVYYSNLSLSGLNFFHITRGMLLGMVATLLTYEIVLLQI

>BmGr9R
MPPSPDLRADEPKTPCLVGGAHAFILKISSFCGLAPLRFEPRSQEYAVTISKGKCFYSYILVTFLVICTIY
GLVAEIGVGVEKSVRMSSRMSQVVSACDILVVAVTAGVGVYGAPARMRTMLSYMENIVAVDRELGRHHSAA
TERKLCALLLLILLSFTILLVDDFCFYAMQAGKTGRQWEIVTNYAGFYFLWYIVMVLELQFAFTALSLRAR
LKLFNEALNVTASQVCKPVKKPKNSQLSVYATSVRPVSCKRENVIVETIRVRDKDDAFVMMKTADGVPCLQ
VPPCEAVGRLSRMRCTLCEVTRHIADGYGLPLVIILMSTLLHLIVTPYFLIMEIIVSTHRLHFLVLQFLWC
TTHLIRMLVVVEPCHYTIREGKRTEDILCRLMTLAPHGGVLSSRLEVLSRLLMLQNISYSPLGMCTLDRPL
MVTVLGAVTTYLVILIQFQRYDS

>BmGr10
MTMSIKPRLQCMVPPSLALALRVSRLAGIAPLKFVAKQSNIMIRLSTSLCVYSYLLVTALNVCTLIAVMID
FSVPVKLSIRMQTETKRFVWIADVVIVGMLSGVGVYTAPIQMRRLIAYLHRIHKINSDLQTYSSSLTDKML
HRLTIGMLLITSVIIVTDFTFVMYLADLNHRQLLIAIMYWCYYCSYFIAHLLEMQFVLIAALALSSLKLVN
NGLRTMLHQSGIESLTEIPNSNEQHAANAVLPQPPKKSVNNSIDTLAFVVTKRSVRFPTAGWTDQRTIRRL
ALSYGSICEVVRQIDNNNGIIVLLLLASFLLHLVVTPYYLIISFVTESPHTGFEKVLNPILQTVWCLYHTF
GLVMIIEPCHRTHEEMETTRELVSRVMCSADPRDPISVELEMFFRQLVLNKASYAPLKVCTLTRSLVATIL
GSITTYLIVIVQLEIKNMQ

>HvCr4N
EVTAAPVPSESGSRPSRPTHCVVGGAHAFILRISSFFGLAPLRFESRSNGFTVSISGAMCVYSYILVTVLV
ICTLFGLVAEINVGVELSVRMSSRMSQVVSTCDVLVVVATAGAGVYGAPRRMRNMLKFMENIASVDTSIGG
QYSRVTERKLCGIILAILIFSVLIADDFTFYALQAKKLDREWDVVTNYLGFYLLWFVVLILELQFAFTALS
VRARFSAVNDALALTARQVSIPVEKPKTSSPLNIYAIRVAPVDSQRSANVSLLVDTMTGREHVVIIKRTAS
GEPRLIVSPCDAVRRLAALHGTLCDVVNSIDDSYGLPLVVILISTLLHLIVTPYFLIMEIIVSTNRIHFLV
LQFLWCVTHMLRMIVVVEPGHYTIAEGKRTEGLVCRLMTSAPSTGVLPSRLEIFSRQLMLQSVSYAPMGMC
TLHRPLIASVIGAVTTYLVILIQFQRYDN

>BmGr11F
MKPFRFFLFVENVICVYRNYSFHKRYARAIILSRVMFEVSLIILTLHSCRNFGAVKYKTEIIFTYLATASS
TILILLALYKTNRFTELFLNFKAFYRNRNLDVDHLEKWNRKQKMATVIIVLFCVIKFSTLIYTDLIGEYST
PCRGYFTEYLFYTNLFMCNARYLFEFSTACVVLHLVSEQLDYIAISMDCTMFLYIDISKKNIMSSAKKRKL

KYFDIFKQFEKWTDAYMNVKRSANLCDTVFRAQLAIMITTITLYYIILLYGITSFNIERGKFSVVKSLSYL
ISLFGFLIALLLLSKAGQRIQKSAENLRRKLSKFLLHSLEDPEFHRAATNLLRLVCTHHIKMRCFGFIDID
MTLLPSCLMFVTSYTVIALQFNNVV

>BmGr12P
MKNLKLCRTTFYFKIIMCSRFISGLYFTATSKKWISYLYKVICVLYIICITRLFYAKEDTFKPLVFXQFIG
NSIESLRTGEGHVLKCYSTIFSLKLIRNYLPDSNNHIPISSITNFLVIIWKVFDQVYIVLMHYFYTTDIII
HLRILSILTTIGVNLSLMPIIVIFELMWRAVKALRKSLGEHLKGPVLIEGRERLKAQQILRCLNVYKDLNA
TLKFNSTPMKTMILISTLATFIRLTLFLYQAILGHNEGLHLPRKILAIIYYALPVCLLGVLMELVARECDK
LKTLMTKELLVCKDDSYCTVIVDAVSYIELNPLKFSILRAFNVNSTLILGLTNLCTTYLIAVIQFTYSCED
INGLSHSHSH

>BmGr13
MEDSFNRLLSIRNMIIFQNVCGFYHMCTEKLYISRIIKMYCVALAIVLSVFCFQNPDITYLSWDVVWVTFG
YTLNVIICLRYNGNYFFQYWNGLHEIDIKMNLTSIDKEKVPISRAVFTVFLILRSTAFAMTIFVFGYLETG
ILSNTIISIYSINLTEFYRNMSNIPMILMFETFYVRIKILKEQLCSELSTVLGCNNDARQLKLILKYLRNY
RSLVRHLMDTTLPFKILILVILVGSFLRSLLIGYAFVYNSDQIILLSLPVMFSTKILSEVVEIKLICTKEL
LKNKNEGLVLLDLDSKKPTFLTSKACGEQLQDALSFLNNRSYSYTLLQVIEFDCSLAFVFTSFCITHLIVV
VQFTHVLD

>BmGr14
MNLHKNIIPIRNNLFANKVTAIALPKTLSVLFKLIHIFFLLDLGVYEYKTFKIKCIVKFLTISGSLTISVV
CFSFMVSNLSEHTFVGWYGFFISTYIFVVLFFNLSNRMTFVEFYKTLLRFDANYGIDSNEYKFNFKIIFVN
ILFIANRMVLSFVYCSYYPQNCIRPRYAQILFMLPWLTLDVLLTTNMFLFYATYCRIAKFPMLIKNSMNIV
ALRNSYKLIVDSLEKTQTSFDIVFIIALVFSVPEIMMSIYSTLLEVISKHFLEVASILSLNYVAIAQSLLL
TLAPSLCAGVLPWKTNNIKIILHEKLFTEKDKASAREIELFIKYIESRPLKLRACNLVPLDFSLTIIVLNI
CVTYLIVIIQFTHLY

>BmGr15
MISSSDINHKRNKVFAYNVPGIALSKTLTVLFKLLHYVLLLDVGIYEYKTFKNKCIVKFLTIATGVSVSIV
YFCLIATVLRKNAFFYWFYVLFISQYMIIVFIFTLSNGMSFTDYYKMLLRFDAKYQINSNNYYFNIKIILV
IIISILNRIGMAIIYCSYYTKNCYEMSFSQIIFVLPWLTRDVILIMNVFLFYVTYCRITKFPALLENTKNV
GSLRNSYKLIVDSLEKTQKPFDFVFTISLVFNIPEIMLSIYFTLLQVIHSHFLEVAPTLSISYFSITHSVV
LILAPSLCAGVLPWKTNTIKIVLHDKLFLEKDKNSARNIKLFIKYIEARPLKLRACNLVPLDFSLPVIVLN
LCVTYLIVIVQFSHLS

>BmGr16F
MIMNLTTDRISKRNKVFAYNVPEVTLPTTLKVLFKLIQFTLSLDFGVYKYKTFKMKCVAKVLTLAGCLAAS
AACVSLIISNIFENQLFFGWYTLFVCQYTIVIFMFTFSNGMTFIDYKMMLLRFDAKYQIDSNVYHFNIKIV
LVVVISVTSRLFLCAVYCIYSTENCIKPWYNQLLFFPWLSLDIVLIMNMFLFYATYCRLAKFPSLFENPKN
VVPLRNSYKLIVDSLEKTKKSFDAVLIAALIFNIPEIMMSIYYTLFQVMNKHFQEVAPVLSLSYFTIILSV
LLILAPSLCAGVLPWKTRHMRLILLEKLFAEKDKNSAREIELFIKYIEARPLQLRACNLVPLDFNLPVIVL
NLCITYLIVIIQFTHLF

>BmGr17F
MGFSLGTTALSMFFFEKPVVFTIIQITMIIVKPAKYKLSDPFRPKDTSKLSESIIMYFKLFHIFLGIDLGG
FRYQNRQVKYAVRLISLIQPLAIYGLCIYALLKIIANTEFLWYTISFTEYVAMSVAITLFSNEMTYCNFMI
NLKFIDTKLKIGDESFRIGVKLISSTILIGVTRCFTTTTYCLLGFCAKPTAAQILFQIPWLTIDLMLLQYM
FIFYACYCRLVKILRILKKRNTDIEEMRRIYKTLVDVLDRARAPFDLAYLLGLLFSIPDVLYSIYESIIKV
GEINTAKALSMSIIYITNIQSLALMFAPALTAGFLPSLTMKMRIILHDKLLEEQDKKTYRHIVLFIKYIET
CPLKLKACQIIPLDFSFPIIILNIVVTYLIVAIQLTHFL

>BmGr18
MRRSTKVISMVNQSDKGEIKTCSRFMKIYFFVIYILTGFNFGFYTGRGLNFLRVIQASVLLLRFIIASNCI
YIAFHFRLLEAIWYSLTFSESLAIVVCFMLSRSALSCKNLFEYLYSVDQELKKSVGPSIEVKLALYTVVVS
VLRLTVYVFCAIAYYETLHEGFCVELVYNTPCYCSDLYLVIHFTIFHSVYCRLKALRISMNEKFDVYKGTL
IYKSLIDNLEEIKKSLDVPFFVILLNAVAIAMINILVTLEISYGQTMKFIRTAPRYLETVLLFSSAFAPVL

AADMMASEAQKIKVTLNNILQRDDSLLEDDRRKVKQFAGYVSARPFRLRACRVLSLDCTLPVTVLSICVTY
LIVVVQFTHLY

>BmGr19J
MRRSTKVISLVNQSDKGEIKTCSRFMKIYFFVIYILTGFNFGFYTGCGLNFLRVIQASVLLLRLSVASYSM
YIARYSPLLEVIWCCLTASENLAVVVCFMLSRSALSCKNLFEYLYSVDQELKKSVGPSIEVKLALYTVVVS
VLRLIIYVFCATAYYRKLFDGLRLELLYHTPCYSLDLYLVVHFTIFHSVYCRLKALRISLNEKFDVYKGTL
IYKSLIDNLEEIKKSLDVPLFVILLNAVAIAMINILVTLHISYGKTMKLITAAPRYLETVLLFSAAFAPVL
AADMMASEAQKIKVTLNNILQRDDSLLEDDRRKVKQFAGYVSARPFRLRACRVLSLDCTLPVTVLSICVTY
LIVVVQFMHLY

>BmGr20J
MRRSTKVISLVKQSDKGEIKTCSRFMKIYFFVIYILTGFNFGFYTGCGLNFLRVIQASVLLLRSIIASYSI
YVAIHFRVLEAIWYCLTFSESLMVVVCFMLSRSALSCKSLFEYLYSVDQELKKSVGPSIEVKLVLYTVVVS
VLRLTVYVFCAIAYYESLHEGFSVELIYNTPCYCSDLYLVVHFTIFHSVYCRLKALRISMNEKFDVYKGTL
IYKSLIDNLEEIKKSLDVPFFVILLNAVAIAMINILVTLEISYGQTMKFIRTASRYLETVLLFSSAFAPVL
AADMMASEAQKIKVTLNNILQTDDSLLEDDRRKVKQFAGYVSARPFRLRACRVLSLDCTLPVTVLSICVTY
LIVVVQFMHLY

>BmGr21
MAHRTNSINLFRSRPPDIRAGVGEPRIFSKFICGTMFTQKSLVNFDLGKTPRGGDQEHSKFFKIYFLAVHS
VTALDFGFDRNAKKLTKILISMFSISVRMGLAAVSFMSLWGRPNALALGWAPGTLLCENILVAVTYSASRS
TFKCGDLFADLSTIDELFGSACDYRIESKMLLFTATMTVLRVVIYSTSRLVRADGFDFVDVLEVLNNLETM
CMYLFLTVYFFVLFSIYCRFKKLRELMKNDFEIRRANLIYIALKDCTDKIKQSLDVPFLVVLVFTVLVVMV
DVFITLEMIISNKYNMAVYVVRYLEITLDFLMLFAPVLLADMMAVQVDGLKITLHDRLCLNNGVDMKRYSS
LAEFIGYVEARGCRLRACRVVPLDLTLPVTVFNVCVTYLIVMIQFADLY

>BmGr22J
MTRFEFSQRTKTKSVSKTLNILFNCIYVLTCMDFGFSLGVYKRMKVLENISLVLRVMVAIMCAAMVMKQDI
LDSAWADITLTESLLVIVSFKLSKPKLSYRELLENLSIVDETQGAPPAGYKVERKLITYIAGVTALRLTVL
CLYCVAHTEQYSIDNFIEFLYNVPCYCLDLYLIVHFIIFHSIYCRLRTLRKALSNNFDVYRAHLIYKTLID
CTEEIKKCLDIPLVVILIATILVVMVNVLVTLRMLFKGESIISAFLLRYIEVILSLALLFVPVLVADMMAL
EAHKIHIFLHGRMQTANDAAEERREVRQFAHYVGTRPFRLRACHVLALDSSLPITVVSVCVTYLIVIVQFT
HLY

>BmGr23J
MAQFQIPSMAGSGLNVAPFSRGRRHEGPKHSNFTKKYFLLVHLVTCLDFGFHRDNDTKTYKWFHAANIGVR
LVLSAYVCSVSLSQDLSFASAAWTILNNSKHLLVVAIFTIFKPKSSCAEILKDLLMIDEALKIHRGCDVKG
QITVCIVLVTAARLLIAAASSLSLHEAFSASVGAAEVLYSFQSYCLDFYILANFFIFYSVYCRLKNLRRVL
QNNFNIYRGNMIYKVLVEHMDDIKKFLDIPFVTSLLVTVIMAMINVLKTLQLIHDGENDVLTIVLRYLEMF
LSFSLIFAPVILSDLMSIEADNINVVLHNYIYETDAAEERERRHLSQFAWYVSARPFRLRACRVLSLDCTL
PVTVLSICVTYLIVVVQFTHLY

>BmGr64F
MKISLRKIVSIRNMTLIQNMFGFYHKFTDNRAIGVLLKIFCGFYSLFLSFLCINCTPRFTNDFLTYDIFFF
VIEYLTSVLVCLLYDGQYFLNYLYDLKLIDREAGIEESLEKLPISQPLFSLIFITRVIYLLSCLLMFDGIK
DSLFLPAQSSVFGANFTEFARTIGYFPRVIMFEMFYKRVNYLKSQLRNDLAHANLYPIGFVCSKVIMKYIN
FYKLLLRNLQQNSLQFKILMSMSSLYIIIKALASAYAFIYREDGVHVFIFIEFATGVFLFFVMSSIIISIF
NEIEDIRQIVLAQLRYCKQGANTKRVQDALTILNIRCFKYALCRIYTVDFTFILRILDVSVTYVIVLVQFT
HILD

>BmGr65JI
MKISLRKIVSIRNMTLIQNMFGFYHKFTDNRAIGVLLKIFCGFYSLFLSFLCINCTPRFTNDFLTYDIVVF
VIEYLTSVLVCLLYDGQYFFNYLYDLKLMDREAGFEESCSQKLPISQPLFCFVFITRVIYLFLCLLMYDGI
NDTLFLHGQISVFGVHVFIFIEFAIGVFLFFVMSSIIISIFNEIEDIRQIVLAQLRYCKQGANTKRVQDAL
TILNIRSFKYALCRIYTVDFTFILRILDVSVTYVIVLVQFTHVLD

>BmGr24P

CINKKIQSIIKSLVSIRTIMLVQSILGFYHKMSNNFFVSFLFLTYTTILISVLSFYSVNDVMAHKFAYTLS
MILEYDINTILSLITAGRQYFNFFEEMKKIDFSIGFGELNIEDLPLSRTLFVTIFVTNILLSIMTAALILF
FSTPFLIISSGSTYAMAIVFFGLSLNVLPRIIIFELIYKRIKYINLSLKRKLKALALECDHTIARFEIINE
NLIIYNKLLQSLGNVNVSLKSSILLTTFTCFFRCSLICYYVITMNEDKVYIMQIIELTKQTLFLGVLIILA
EYIKNEIENLKMTVSLQLFTCTDTQLYHQVSDXFEIHRITPIQLCGFKNMSVDTNLFLGLINVCSTYLIII
TQFLNAYVN

>BmGr25J

MFVKCLKYVKKFKPMFSVMFIMNFRLICGLYYRIHSDAFVCFVFKVYCILCSMFLFFTSSDLAAPFSRSIP
ILATLFEYVANVLDCILTGQSYFFHLRMELMRIDPRLRGLDRPPASSIVFTAILSYKIFILAVYIHGKART
TYLQYEWFSSIGIHLLVLFSNLVHMNRMLIFEMVTFSLEAQKKTLGELLKSSLRRERVERKCEILNRFLKT
YKRIIELFNNTMAATKLMTLISVVSCFIRILTYLYQVLTTQLSSSSAGSIFTTRHLTFIVTFVREHVLSVY
FLKKVGICQRLQINFKIKCNVKTETLELISQDDEYTEKLEDALDFINSCSSKITILRAMTVDATLPLTFIS
LCTTYIIVVIQFSHIYD

>BmGr26
MNKTKIYRKKLDKNERLVCSVQPAMFARLIVGLYYDIKVSNRVKWMIKSYCISLSSFICYLIIFRDDNFSL
HPKLTSVMEYITYVTFSFLTCDKYLFRYLRFNPRTDGYPIFLYLCKKFEKFFKIIICLFVSFKILGVVLMM
QSWPILSTPKYIWGTLALHFLWLASHMGRLVFILVYGILFCRMRTIRIIFENRGFQNTPQNRLTPKRYILM
YEAVLNSIESVDFPVKFLIFTFICCFAPKLVVSLFEIMEEMKKGELSLTTFIWFLVELSPSYLFLLLSAIA
LDLVSEDVQELLSITIDRRLNCKNEKERSEIQEFFQYLRNNPFNYTLWQVVSLNLRTLLVATSFSIANVIA
IMQIKNSKI

>BmGr27
MVFKYKIMTKAPKSLPVLKILMLFRLVFGNYFRLSSNRYINFLVKSYCSTFTILLSVMCGKRLKNDSPYML
SLTEYILNKILNYATSEGYIFKYCNSIKTCDKIMGFKKLPIITIDVFIAIIITVITRTAITIYFGFLFPFD
KYQVVLYVGCIVFSNDLNSLTIMNVFGLLNNRMNLLRKSLEAMTVPINIIGKNEVAPKVRLVRNAFRYYSN
LLDNLDSVNHCVQYSLSVTLLLKFPKAVLLCYDSIKTYFVKIDNNFAMDIVDPTEIILSIVVMSFPAMLCE
MITNEVEKIKAILTKHLIQCSDNSLRFELNITLLYICHRPFKYILWRAIPLDTSVPIGIVSLIITYVIVLI
QLLHFST

>BmGr28P
MAHKIATVGPTNATATVKNNKRKLKISKRVTIFKVVRCLRFILGHYTELTSSKLKAFLIKCCSLLLAVIII
YAPLNYIKMAYVMGLIEYLLFVLLSLFTGDEYFYKFHNSIKSIDVLMGYKRGKIIDSNAIIFLLSVITIMR
IVIIYCRSTVLAFRFTIIGVYLAIFSLRISYMLITVIFFAMYHRMKFLRKKFEIITIPVTIIGKQKVASKI
RLIRKYLINYHHLLDCLRDINGGLQYFLAIMIACNLPKYIFFAYSAIKIQVLEHITIHSAVQNVKCZRILF
VVVPAIFAELTTAEVERIIDVINRQLLRCTDEHMELELKVALEFIRRRPFDYVIWRTVPLNASLPIAIISL
CITYVVIVIQLTQFHDNF

>BmGr29
MYLRSKKSRFKLFSFERMIKILLMICGHYVQTDSSNVVSSIHRIFSIVITICLCPYFQFNPFFFHVIESVW
YSILSQFTQYGFFFRYCSTIKTFDLLSGFKQIPLYTKRVCFFLLITLLVRLIIVLIHFSAHQTKLKTFCAF
LIILSANTGHILMTIMFSILNTRMTLIQKLFANNPIPVNIVGKNQNASHIKRVRKGLICYNNLLDTLKVAE
KEIQFTLTVTYLCHVPTIICYVYFVITVIYKSKFSGYNLIPMLDMILACMAVTAPALFAELTKNTVDKIKK
ILGSQLLRCSDESLRYELEITLEYVIQRPFSFSIWRAVSLDASLPVAMTSLCITYVIVILQLTQLRP

>BmGr30S
MYLRSKKSRFKLFSFERMIKILLMICGHYVQTDSSNVVSSIHRIFSIVITICLCPYFQFNPFFFHVIESVL
YSILSQFTQYGFFFRYCSTIKTFDLLSGFKQIPLYTKRVCFFLLITLLVRLIIVLIHFSAHQTKLKTFCAF
LIILSANTGHILMTIMFSILNTRMTLIQKLFANNPIPVNIVGKNQNASHIKRVRKGLICYNNLLDTLKVAE
KEIQFTLTVTYLCHVPKIICYVYFVITVIYKSKFSGYNLVPLFDMILACMAVTAPAVFAELTKNTVDKIKK
ILGSQLLRCSDESLRYELEITLEYVIQRPFSFSIWRAVSLDASLPVAMTSLCITYVIVILQLTQLRP

>BmGr31JI
MYLRSKKSRFKLFSFERMIKILLMICGHYVQTDSSNVVSSIHRLFSIAITICLCPNFEFNPFYFHVIESVL
YSILSQFTQYGFFFRFCSTIKTFDLLSGFKQIPLYTKRVCFFLLITLFMRLFTVLIHFLAYQSKFVTFCAF
IIMLSANTGHILMTIMFSTLHTRMKSIQKLFANNPIPVNIVGKNENASHIKRVRKGLICYNNLLDTLKDAE

KEIQFTLTVTCLCHVPKIICYVYFVITVIYKSESLRYELEITLEYVIQRPFSFSIWRAVSLDASLPVAMTS
LCITYVIVILQLTQFRP

>BmGr32S
MCYTNFVSRQVSKCINFFSTIRYVIYLRMFCGLYYNCSSSFKIRCIARLYCFIIYCLNLHYNLYIFTSSVS
LTNFLHTFITLAEVSIHILFSLYTGESNFMSFCIEMNKLTSGPIDFVATKCVATHFIAFFVIGLHILSSTL
ICGAEVSCFTFSVVLASMTFLTTLLSRFTTIIMFDLVWIRMRSLRKILVNALESDLSEDEKVKSIESFLKA
YKQIIASIRITKLATRNLVTFNFVSLFGKIMTLIYFCINCPGYLNTYLISSWIFGILLAGFVTCAPPVLVE
MNVNELDEIKYALADQLVDYTDDNYRTAIYNALDYVEVHSIRYTLWKNFPMDLTMFFGFAGFCATYIIGLL
QFTY

>BmGr33F
MCYTNFVSRQVSKCIHFFSTIRYIIYLRMFCGLYYNCSSSFKIRCIARLYCFIIYCLNLHYNSYIFTTNVS
LTNFFHTFIILAEVSVHILFSLYTGESNFISFCIEMNKLTSDPNEFIATKCVTTHFIAYLVIVSHILSSTL
ICGARASCFTFSVILTSMTFLTTLLSRFTTIIMFDVVWIRMRSLRKILVNALESDLAENEKAKSIESFLNA
YKQIIASTRITKLATRNLVIFNFVSMFGRIMTLIYFCINNPGYLDTYHMSLWIFGILLAGFVTCAPPVLVE
MNVNELDEIKYALADQLVDYTDDNYRTAIYNALDYVEVHSIRYTLWKNFPMDLTMFFGFAGFCATYIIGLL
QFTY

>BmGr34J
LTNFFYSFITFAEVSIHILLPLYTGESSFMSFCIEMNKLTSGPNEFIATKCVATHFIALLVIVSHILSSTL
MCGARASCFTFSVILASMMFLTTLLSRFTTIIMFDVVWIRMRSLRKILVNALESDLAEDEKAKSIENFLNA
YKKVIASIRITKLATRNLVTFNFVFMFGKIMTLIYFCINNPGYLNTYLISSWIFGILLAGFVTCAPPVLVE
MNVNELDEIKYALADQLVDYTDDNYRTAIYNALDYVEVHSIRYTLWKNFPMDLTMFFGFAGFCATYIIGLL
QFTY

>BmGr38P
MKPKYVTNIKLVLFLRFLCGYYYEMEIPRRLKTVAKAYCIFFLFFYLVLHHLYCSFSNHTAKWSLYLEYSI
YVFMSLYSKKMYLMDYYTSSRIIDFEPHSRIYKKLNIYLAILIPFLVVLKIVNMVTFCLSKSFNCWSWVSL
LHNLLWNFTVLGRIPPVFVFALLFCRTRIIRRTLVSITVGPGSTVLKSLFKCTRYWLTVLKRPNTHLNSXL
TVFLLCSTPKLILETFLMLNKIKESGPIVEKLAVYAIETFYTHLFFVVSSILFDLINVDLQRIKILIVEKR
MKTKNTKHRIEVEKLFQFVKSQTIECTLWRVLSLNVRNILSFVSFAVTTIIAVLQIKNNNIY

>BmGr40SP
MLPICYIKLVYNISSIMFLRLIFGLYFELNVSNRMRWLQRMYCVFISIVIMYFCYFKTYRILMTVKVPFIL
EYGMHVILSLYLRENFEYTRYDPIIDSAADTKNFYKKFDIFIKIFLFVAILLKMZSMLVFCVLWSGLCYTG
FIGICGMNFIWIATFMGRMVLPLIFGILLCRIRMFRLTLQKQGFDNLPYNRFSPRRYIMMYDSIVRGLEKT
DFPAKNIMFVFTICIYSKILTGLFDLISVLKREGPKLMNVMLFTLEFLPSYVLLMIYSVTLDMVSTEMKEI
LKIVTEKRVFYEKQHANIQELCQYIKNNQLKYTIWRLVSLNMQSLLRATSFCIVSTIAILQIKDWNG

>BmGr47JR
MKIRFLFGFYCDFPFNKRFQNILKFYCISVLVVLILGSWACSTGFRSDKKIVIYCEYIAYFLISLSTKDRY
IFDYYKQQPLIDGSTTSKVLYKKLERLLKYFVTITIVLKMLNIFVFCGWNLTKCINELDGVLFINLLWIGL
LLARLSLPVIYGLLYFRLRVLRMTLESKGFSNSPQNRFTPKKYITIYEKIMKDLLKMDYPLKYVFIIFLIG
SVPKLLQNSWQFLNSLKNYGPEISKILEFTLECLHSYIVIILPIVVALDLSEDEIKKMKIITLNKRLACLN
ERQKMEIQQLFLLLKNNSLRYNLWRVVPVNLKSVLIFLSFGVTNAIAIMQAKNLN

>BmGr35S
MYSSLKLKDYIVNMESVMCSDQSITISFKNVFKVFFDYISSLDFMMVCRLCFGYYYEFNCSNLCKIMFKCF
SISVCIFCVSMHLVQLISPPYLNHCVIMLESTVSIITSLVTEDKYFFEFCLDMKDINSMMNQPRNIKSFKI
IYVIISGAICHVIRHISICREKALSFCFSTEYLTASFTIISGYWNYLNITMMFDLLYQRLVAVKQMLTNGL
NICDTDEYKIKSVQKFIDVYKALTVSLSKTSNLIKHTVSLGIFCALWRIIFFVYYCISMDFQVESAQFITW
VSSMCLSVFLVYIPALIVELCSNEVDAIKWILASELLEYRDKRLRTSLRDALDYIDVCPIDFEIWHCFPMN
LSLCLGFIDISSSYIISILQFKY

>BmGr36JP
MTVPYDKIKSALSKFVQLLFSINVVLFVRFLFGFYMKIGSRKYFHIATKIWIVTLTIFRVYFQCRNFMNYP
SYALILHDFTCTVELVILCIISSLGGEQHFYTYCSEMAELIDNRKNKRASYFTTSTLLIGFIILIVPTSIS

CKKVSNALSMLFLNIFNYVACFMHHLTIIYVFELLWREIRKCRISLERLEIMSVDDKIMKIENFLDSYKRS
LDSLNKANGVMIPTMALAYFAIIAKIVFFTYNILSLRGFNILVHGDSWLLSTSIAIIFICAPALLVELAAN
EVNKIQNLLAVELLKMKDDKFZYRATLYDALDYIEVRSVKSMIWRNFPMDLNLCVGFINLIITYVIGLLQL
LY

>BmGr37JI
MSAKINYQEKLCSIKSIMYLGLFCGLYFRSSTSRMMLLMTKVYCIVLLFLGISFHLNMLSSDLPTETTLHS
SIIMLEFFIHIITSLSTGQAKFLHFCTEMMKINGHNSKGSLDFQLVITNIALIIIIITQTTSSLLYCIIRS
KCLSHSYVFTVITSLCVLFSSFTMIIKYELIWNTVRSLKNTLVSNLDSFDLSEQEKVNSVYNFLSTYRDIK
ANVDLTIKGTRTTDCLHASARVSAIVLSVAVTCIAPILVEINVYEFGRIKFALADQLLEYTDSKLRRSVYD
VVEYITVAPPTVTIWPEFTVDLGLYMDFIGLTVCYCIMILQFEY

>BmGr39SI
MNVSPQYSVLKIFKPLFKVQTLFGSVRVKVGDNGITKTTKLQKFYSIFNILFATTGHFYTSFVYSVCVPCV
GNSVAETSMALIQIYAGHLMNSFIVFSNTFLHNEKNVQMFKSLCSIDELMKIVRLEHRDLKLFIAIILLLS
STVIMNVYFLIYMVIILPISEKWVPFANIGIMNEDLEAITFVSVLYMLYDRVKYINQTALDPVNIAKLIKE
SDGQNDEETVSRILKAFKEISKAYKIVEKTFRVFNLSWGTFMSKNLIGVKFIARIMLISVCVSYLERELQK
TKGLCNLAVRNCENDIVRCHLKNIYRIIDTEIEPMTVFGLFYINNVPLDLISLTATYTVVLLQFAFL

>BmGr48F
MSGNYLEEYINMSFSPIYKYQKFLGSNRISLKAKNKITVANNWEKLYAFLWMLAASYSIHHFISFFYSYYY
ERSKILFLGCSLGTSSQYLTYILTITYDKFLTREADIDLFINLQKIDRLLKLDRCTVLFKKIRLIYIFLLI
LVTVPFISGFLIHVFDYIDQPYKTFFLGLGLTIIYVDVLVTAFFIANLTLRLAYINDRIAMYYKRSLPLRK
DSGIRRSRSWICGSWIFQIMPKIKNNGTRMKNYTFIKYQSLIFNILKCYRLITEIYSLPVFLITATVSIWT
FLVIGSIVAGSRSEIKLFSIVAMITVGLWNLFFIIQLTSLAFVNDLFLMEVKNTKQLCIRVLSYTRDDSIN
KAVNTILKDIEYAPPIFSVYGIFVFDKSIILFLIGIITGNIMTVIQFSY

>BmGr41SF
MSENSLEEYIHMSFSPIYKYQKFLGSNRISLKAKNKITVANNWEKLYAFLWMLAASYSIHHFISFFYSYYY
ERSNIIFLACSLGISMHYLTYILTITYDKFLTREADIDLFIDIQKIDRLLKLDRCTVLFKKFRLINIFLLI
LVTVPFISGFLIHVFDYIDKPYKTFFLGLGVTITYVDVLVTAFFITKLTLRLAYINDRIAMYNKINIPHKK
YSGIRRSILWIFGWRIFKIMPKIKKNGTREKKSTFIKYPSIIFNILKCYRSITEIYSLPVFLITATVSIWT
FLVIGSLVAGSRSEIKIFPVVAMITVGLWNFYYIIQLTSLAFVNDLFLMEVKNTKQLCISVLLYTCDDSIN
KAANTILKNIECVPPIFSVYGIFVFDKSIILFLFGIITSNVMTVIQFSY

>BmGr42P
MNKTKKIERLSRDILDEDFIDVFKSIFIFQRIFGLLSVNITYKYITETSKLYKLFVMSLWTVNVLCLVDYI
LNYRTSFDVATDSMLKLVMSVNVTTNALIVWRNNFKLNTLKSQIYVKLQNLDRDLKTKDAVTMNKKLSALS
IALMICGFIWCTIWLFVYNAIAMNTFCVPLTIILSANVGNWLEMVLLFIIFYFVNVRAEYVNKLLRRRLNQ
TECPDRVFLIQNAKPSDTVSREFICGMQSLLEIIGNIKDIYQFPIFLSTCQVMLCILVIVQNLIISVKEQT
STMVDSMLCMLPALLLMLTIFFSLCVIAEALTSKLDITKKLCAMGMHSFTDDISRRNSXQIVLLLKAKRPL
SVFKIYTLGTRLPIHLLGVTASYTIVLLQFAVL

>BmGr44P
IFLSTCQMMLCILVILQNLIISVKQZTSTIVDSMLCMLTALLIMLTIFFSLCVIAEALTSKLDMTKKLCAM
SMHSFADDISRRSSKQIVLLLEAKRSMSVFNIYTLGTRLPIHLLGVTASYTIVLLQFAVL

>BmGr43JI
MLKKSAMITMKSPEYLSKDILDEDFVRVFRFPFLVQMVLGSCRVHLKARFITIPTLGQKLYTVMSIIICSL
LYFNITKLYISLYYEQSIVYFLFLTVAGLDQLSFFANLIHVRFLNGETNTGFCIMMQRIDRKMKIDHNNIF
NKTVIRANILTITLIILLYMSLVISTIILKKYSLVTLFGLVHGQLILLVEMAYCSNLIIFFFIRVRFVNAI
IKNHVHPENQNQPPKLVRYFVTNRIMRYLAAQTHDFIVNDTDVYLKQIFEGFSMFIDIYRFQVCLFCIKLI
VMSLLTFEFCLVGIQKNGPLRKKATRMLKIIEESTPQFSIYDMWQMDGYTFVKICSLVTNLIVTSLQFAYL

>BmGr45JR
MITMKSPEYLSKDILDEDFVRVFRFPFLVQMALGSCRVHLKARFITIPTLGQKLYTVMSIIICSLLYFNMT
KLYLPLYYEHSIVYYIFVTVTGLDQLSFFANLIHLRFLNGETNTAFYIMMQRIDRNMKIDHNNIFNKTVTL
ANILTITLIILHYVGLVISTIILKEYSLLSLFGLLYGQLMLMVEMALCSNLIIFFFMRVRFVNAILKNHVH

PENQNQPPKLVRYFVTNRITRYLAAQTHDFIVNDTDVYLKQIFEGFSMFIDIYRFQVCLFCIKLVVLSLLN
FEFCLVGIQRNLLETKNLTNYYIMTYSVIGFFTALYVSGRCELFFREIRETKRLAVAVLLQYQEGPLREKA
TRMLKIIEESTPQFSIYDMWNMDGYIFIRICSLVTYLIVTLLQFAYL

>BmGr46JR
MITMKSPEYLSKDILDEDFVRVFRFPFLVQMALGSCRVHLKARFITIPTLGQKLYTVMSIIICSLLYFNIT
KLYLPLYYQHSIVYYLFLAVTGLDQLSFFANLIHVRFLNGETNTAFCIMMQRIDRNMKIDHNNILNKTVIR
ANIFTITFIILIYVVLVISTIMLNEYSLVTLFGLLYGQLIFMVEMAHCSNLILFFFTRVRFVNAIIKNHVH
PENQNQPPKLVRYFVTNRITRYLAAQTHDFIVNDTDVYLKQIFEGFSMFTDIYRFQVCLFCIKIVVLSLLT
FELCFVAVQRNLLETKNLTNYYIMTYSVIGFFTALYVSGRCELFFREIRETKRLAVAVLLQYQEGPLREKA
TRMLKIIEESTPQFSVYDMWNMDGYIFIKICSLVTNLIVTLLQFAYL

>BmGr49SJI
MAGIRTISFKVKPLELPDVSENNFADDGLKIVQRFKFFIYIQVITGINRLYLLKCNKFVMLFSYLYAIFLI
SFVASVYWTKEPMKNSHLVIRLFSFIEYILLICISVFLKKKKMMKFFENLSMFDQILKIDKNVNSTFCMKR
VFFWVTGSIVYNLIEFYALEFYDNTSKGLMTIICTYTIAPAAIGVCVYLIVHCIKYTLLVVIPCYYSSITA
TQVSLIRITLHDAINTIPLGKLQRRKVKAFYLMTKEYSFVYTLAGVIKLNMSLPLSYISLCTTYLVIIIQF
SKFLD

>BmGr50S
MAGIRTISSKVKPLELPDVSENNFADDGLKIVQPFKFFIYIQAITGINRLYLLKCNKFVLMFSYLYAIFLI
SFVALVYWTTEPKKNSHLVIRLFTFFEYTLLACISVFLKKKKMIKFFENLSLLDKMLKINKNVNSTCCMKQ
VFFWVTGSIVYNLIEFYAMEFYDNTNKGLKTIICTYAIALAHDCEQIFFFTLQRVVYLRLLVVKRHIQEYF
KVDEDSSRKKPNKYEMLSNNVQLNLTALHEVYALLHNCAEKLNTVMSIPVLLMLFTSGLSTTILLKFFVRV
IQLTDPSNPGSAIGVCMYLIVRCIKYTLLVVISCYYSSITATQVSLIRITIHDAINTVPLGKLQRRKVKAF
YLMTKEYSFVYALAGVIKLNMSLPLSYISLCTTYLVIIIQFSKFLD

>BmGr51F
MAMGIRTILSKVKPLELPDVSENNFADDGLKIVQRFKFFIYIQVLTGINRLYLLKCNKFVMLFSYLYAIFL
ISFVASVYWTKEPMKNSHLVIRLFSFIEYILLICISVFLKKKKMMKFFENLSMFDQILKIDKNVNSTFCMK
RVFFWVTGSIVYNLIEFYALEFYDNTSKGLMTIICTYTIALTHDCEQIFFFTLQRVVYLRLLVVKRHIQEH
FKVDEDSNRKKPNKYEMLSKNVQLNLTALHEVYGLLHNCAEKLNKIMSIPVLLMLFTSGLTTTILLRILVR
VIQLADPSNPGSAIGLCVYLIVRCIKYTLLVVISCYYSSITATQVSLIRITINDAINTIAFGKLQRRKVKA
FYLMTKEYSFVYTLAGVIKLNMSLPLSYISLCTTYLVIIIQFSKFFD

>BmGr52J
MAGIRTISSKVKPLELPDVSENNFADDGLKIVQRFKFFIYIQVITGINRLYLLKCNKFVMLFSYLYAMFLI
SFVVLVYWTTEAMKNSNLVIRNFTCLEYILLICIAMFLKKKKMIKFFENLSCLDKMLKIDKNVNSTCCMKR
VSFWVAGSIVYNLIEFYAIEFYDNTNKGLVTIICTYTFALAHDCEQIFFFTLQRVVYLRLLVVKRHIQEYF
KVDEDSSRKKPNKYEMLSNNVQLNLTALHEVYALLHNCAEKLNTVMSIPVLLILFTSGLSTTILLKILVRV
IQFTDPSNPGSTIGVCVYLIVRCIKYTLLVVIPCYYSSITATQVSLIRITLHDAINTIPLGKLQRRKVKAF
YLMTKEYSFVYDLGGVIKLNMSLPLSYISLCISYLVIIIQFSKFLD

>BmGr53
MAHIKDENQSKQQQKEHETLNKNKLKKVVYTLKPALMLENWFGLSDFLLVNEDELVLLMQTEKFGVILSIF
FIVMFAVFVDFPDTETESIMELMDEVPSMVVLSQYFIASITTSSCLSAIAIRIFETFADLDSMLLITTTQD
FYNKSRYQTNKYLIILGVSHIISSTLDLLTDDEIVWCKFFVLPIYFLQKLEVLTFCKLIVMIQCRLQIINK
YLTNFIEEQEKNKALVFTLAESNPKKTDKFNWIGCPSPNNMKIRDLATMYDVIGTICSLINDLFNIQIFMT
LVSTFTYIVIAIWSTLYFYRAPNFTFGTLTTIIIWCITIILSVVVMSFVCERLVSVRNNTKILVNKVIMNY
DLPKTMRVQAKAFMELIESWPLKIMVYDMFSVDISLMLKFISVATTYLIVIIQLSHFV

>BmGr54S
MITIKNNFGFRQNNLSFYRPILIIILQLCGYDFDYYNINLVLNVLTKAYCASLTCVVVYATIACCSSIQLSH
IWSLIEYGTSVVIIACFRSQTKLFLKQLTTLDVYLRISNRRFVLEKCKIFTITSVIFLLRIVYTSIYCSTH
HCFNVLIYFLLSQFALVCLDVNRIWRCIVFDAIRYRLKTLRLRMEENPDCNYYLYVKNNKSIRKNKISFCL
FLYRTIADLVDLVSPELNVSLFLSVACSLPKIVSNAYHLLLIIEDREPLETGGYVLMHTLQVSLLLFTPFI
IVECYTMEVEKIKLYLVHRLIDENGEDTTMRDNIRLFLEYMSVRTFRYRIFRIVPVNATLPLELVNLCVNY
VIVLINFTHLYG

>BmGr55
MERINLLKSFAFLENVMCIYRNFMFYNQRARFIIIGRIVAELVFYIFSAYNGFLLVYTDWFSQNFSVFFIE
IISKSSFYVITFFTMVNGILKSREYKTFIFSINKIHDYILNDTDYLKRLKCTNIFCTATIIILFVVTLIRT
AIDGSNYGQLSGINARSVIWMLTTILLECQYQTECVVYFGFILFIHAIMKYLNIRVTNTIIKIARSDMAVK
RIPKYIIGRTELKDETDTGVDVNNVVDLEEVRYWVFIYRQLGLTTELLQKCFGMQTAFIFVTAVLNQIITV
FRVIAVFIYGSLANRGAEHSIIANFLFTLLYRLPGLLMIIVGGQMVQNQTDMLRRSMARLNNIISNPHRET
FSALSDFHRMIVKNPVKIYVLSVLPVGAYMLPLFMTLLINHIIILLQFNHVA

>BmGr56
MKKIRLLRSIVFLENLLCIYRNFLFFNKKARAIILIHITIELVLYVLSIVNNSFIIYSYFHSDNRSMLIVF
TTICCFYVVTFVSIVMGILRSEEFKDLVTSLELINKFFTNNKTYLKSLGRSNTMIIAITTILYCVTCIGIA
VDKITLNDFYEFTSSDVIWTVSSTLLELRYQTECVVYFGIEYLFLIFTKHLNLLVKEAIKKVSLDNNGTVK
DVPISSDAVTKNEVKRWATIYRQLMMSSKLLQACFSLQIICVFVSAVINFITTAFRMVKVSVLGSIATDMN
EIIIVNLIFTLLYQNIGLVLIIVTGQRVWNQILLLNVLLARLYNGILIQPCRDTLRTLKNLQRMVVKNPVQ
IKMLSVLPVGSYMLPMFMTLSVSYIIVMLQFGHVV

>BmGr57
MEEIKAIKLVTFIENCICVYRNYAMCTKRNKKIISLRIIVEIIIVFFVNINNILLLHKYYNGSGLLYIIYL
FLVVYYINYMFCIFYGALQGKAYRQLIFCFNKINAIAKRDKSYKKSLARLKNMCIVISIALLIISALSVFV
DRSNSWNIYEVSLRDSLLILSKIHMDFFYHFEYVVYFTHIKIFHLTLRYLNSRVKMAQFEMKMTRRDVHDE
GERNIRILLTKELTTEWAVLYKCLVFGTKTMKSLFGLQMLIAMVMSFVNFTLSLYGIILICSIEQSQTASQ
HNLLLILTYYTATMLLIFIVAQSVYNEVEMLKRNLARMYNILAVDSDETQQKLVKDFLRMVYKNKVEIKML
SIFPVGMPMLTFFLSLSASYVVVMVQFSNVF

>BmGr58
MSSRRVLYRAEVLLSNNVDAHVQDMLKPLNFFQFILFFPKYTIRDGYITPNSLIRNIWSATGAFVFISICV
FRILTMNKIAVYDTFTTMLLISKYFDVALYCIGFIVNTYVNIAYSNVNVLLYLKLQTIKTFIPRNNEIMKN
VKWYSVILIIVLFCGTLAMFSFFHLSFSYFNIFDLTTDLAVFSFDLNLVYACSVLNFLAQSLDELNKEIWR
LGNAKVTVCKDGSKPDWNGINLTYINVLDAYNYFKEAFRLLIFFHTFKTLTHMFIYIQSIIELCKKFYPGD
DYDAITVGAVVGVWFFRNITLQCLVGVSCQNFYSATSNTESICAVQVGSIVSDEHKLFLKAVRRLNNVVFY
KWSMYGMFIVDATLPRRLIELIATYTVVFLQFAFK

>BmGr59F
MPYKKDSNRCEVLLYNNVDTDLQDMLRPLNFIQTIYLSPKYTIKDGYITPNSLFCNILSAAGAIVFFSICV
YRILTASKIGTFEGFSTTLLITKYFDAILFSLGFVANAYVSIRLSHLNVLLYLKLQAIKTFVPCKKIMQKV
KYYSIVLIIGLIIVRLIMYIHFHWSLGYLSYLDLITDLGVISFDLNLVYASSIVKFLGYNLEELNKEILRL
DEIKATMDEEGSKPDWNGIRRTYLKFSEAYNYFKDAFRILILFHTLNTFAHVFIYVQSVIELCKAPADNYS
AAFSVLIAVIVWLLRNIILQSLIGISCQSFYSATSNTQSICSILVRSVLSDDQKLFLKTVQRLNKANISKL
DVYGLFIVDATLPLRLIQVIATYTIVLLQFAFQ

>BmGr60JR
MLTPRSDLCNEKLSPSFPSGKTTAADKDDTEARCQVDSSLERLLLPFNLVQHVSFIPMYSIRRGLVSPDGP
LAYLYSLLGFCLFTSVSVYRNAIMHGTRLSSLHLFTLYSDLVSFVINYSLSLICNVVNSKSNVEFVCRLQR
LQTVLRRNQREQEQFARSNWAHLAVVTALYLAVVGLLNVVVLKQSLPDTLYLLLLFCIDVNVLYATRMLAL
LRCYLQLWTRKINEKAFNPVHHNMFTAYLDILQEYEVYTTLFKKIITYYVLETFLHGLLYVQVAIQICKSI
RRSGRFSEQLMMIVSIFTWTIKNMIIMTLHNVECEKFYLAVEQAVAACQTQRASTTRCREEKRLYKNVCRV
SRAAFSRERGWGLLAAGAALTLRFMDLATTYVTVLLQFAFVSRT

>BmGr61
MSIRFEKDLLHNYVEIELQYFLRPFNVMQSLFFQSKYRIVDNFILPNTLFKNIMSFVVSVLCALSFIYTII
SVWQNTHATSFHALVTSVYLSYNIYGILIGSVLIIWLSDRNIEFVLKIQDLIKILEFNKCFLIEYAFINSI
IMAAIFILNFLLYGYFVVHLQKFALGLTFSAIVCILNQDLDIIYVIIFANILKKCASRWTVEARQKNNFND
QGKWVKLFNAFLNLTESYQLYQKIFEFYELLRRVGIVFLGLQLTVCRVCSNDIKSIQCTVMLHAFQLICVW
IVKKFITLSILSFEMEIFYEKLREIETVCIILVSSDNPSERELKIWKNIIRVSSCSVRKTTACGLCEVGAA
LPQWLLQATTAYTIVLLQFHITTFSRARNDIYDLD

>BmGr62

MNDLFLSKIVKWTKTTKYKLDDDFQSLFRVFNIAQAMNLCPKFLIYDKYITNNAWFIHILAISSFIVLVCL
DSFFANFRLVLSEAMGPPFYGFSFYFISILYENIGVIIQITMNGYLTKNNVLIITKLQDTFKDFRTTDYIT
KSNRWTNWFIFFIYMNFIANYSYFNFYVNTFSFHKFCFAFIKMCFDLNIVYTIFIFKMIGDSLTMFKDTAF
CSKNMKLYEVSNRVYWNKMLRLYSNILDVFELSKRTLNFFIFYFVSNILLRILSHVQLAILMNSINWLQHV
AYSNIVMVLLTLAKEGIILIVLIAKCEKIYCVIGDVQTACQLALGNAACPEKRRFCKNVRRSSSAAFSKIY
ICNILAVDAKLAVSLMSVTTTYTIVMLQAILIK

>BmGr63F
MQIGNAVIHLKSTKLTTMNTISPTTKLLKIFALNSNIEEIDLKCSTKLRITMTAFVLCSLIFYSLYYKFIY
VFDYVNISIKITDCVQMVYDFCQYIVDLYFVTNYGRNISSEYFQQYKIIDKILEVVCYEIIKHRIVKLLWV
FMCIWFSSSCFDFIAWFLNYGWITPLVYSVAYIFLLIKILTTLDLSAHIMNVEIRLKMIADLIHHYYMSCE
DNFQAEETLCHKNWLNSKERAKYYELQFRIHALKQLSCNNNEIKLLSRCYLMLTEQVEIINRMYGFRILLN
SLSLLIDMVRFTNISVRIMIGSQNLAYNCGYFPAVSSIFRLLTCGAVIINLVSHCERVYYQRTRICNVIDH
MIVNKNLSRESTEALQEFRNLVQNHPIEFNMANFFQLNYSLLVSIASVVVTYTIILLQSVN

>DmGr5a
MRQLKGRNRCNRAVRHLKVQGKMWLKNLKSGLEQIRESQVRGTRKNFLHDGSFHEAVAPVLAVAQCFCLMP
VCGISAPTYRGLSFNRRSWRFWYSSLYLCSTSVDLAFSIRRVAHSVLDVRSVEPIVFHVSILIASWQFLNL
AQLWPGLMRHWAAVERRLPGYTCCLQRARPARRLKLVAFVLLVVSLMEHLLSIISVVYYDFCPRRSDPVES
YLLGASAQLFEVFPYSNWLAWLGKIQNVLLTFGWSYMDIFLMMLGMGLSEMLARLNRSLEQQVRQPMPEAY
WTWSRTLYRSIVELIREVDDAVSGIMLISFGSNLYFICLQLLKSINTMPSSAHAVYFYFSLLFLLSRSTAV
LLFVSAINDQAREPLRLLRLVPLKGYHPEVFRFAAELASDQVALTGLKFFNVTRKLFLAMAGTVATYELVL
IQFHEDKKTWDCSPFNLD

>DmGr64e
MARTTGDPAKRRRCMSRIKFWRRSRVGSEATLGIIKYRVVEKDTKRFKLSLIKAWLLRIRQEDYKYSGSFQ
EAIKPVLIIAQIFALMPVRKVSSKFAEDLTFTWFSVRSYYALVTILFFGVSSGYMVAFVTSVSFNFDSVET
LVFYLSIFLISLSFFQLARKWPEIAQSWQLVEAKLPPLKLPKERRSLAQHINMITIVATTCSLVEHIMSML
SMGYYVNSCPRWPDRPIDSFLYLSFSSVFYFVDYTRFLGIVGKVVNVLSTFAWNFNDIFVMAVSVALAARF
RQLNDYMMREARLPTTVDYWMQCRINFRNLCKLCEEVDDAISTITLLCFSNNLYFICGKILKSMQAKPSIW
HALYFWFSLVYLLGRTLILSLYSSSINDESKRPLVIFRLVPREYWCDELKRFSEEVQMDNVALTGMKFFRL
TRGVVISVAGTIVTYELILLQFNGEEKVPGCFEN

>DpGr64eP
MARTTGDPVKRQKCIARIKFWRRSRVGSDITLGILKYKVVSNQAQRFQFSKINAFLRRAVRDDYRYSGSFQ
EAIKPVLIIAQIFALMPVRGIGSKLAEDLTFAWSSARTYYALAMMISFGVTSGYIVAFMTNISFDFDSVET
MVFYGSIFLISMSFLQLATRWPAIAQEWQAVETKLPPLRLGKERRSLAHHIKMITLVATTCSLVEHLLSMT
STMTYSVACPRWPGHPVDNFLYFNFATVFHFVDYSTFLGLLGKVINVLSTFAWNFNDIFVMAVSVALASRF
RHLNDYMQREARSATTVGLLDAVQSQFRNLCKLCQVVDDGISTITLLCFSNNLYFICGKILKSMQTKPSAS
HTMYFWFSLTYLLGRTLVLSLYSSSINDESKRPLRIFRMVPREYWCDELKRFSEEVHMDTVALTGMKFFRL
TRGVVISVAGTIVTYELILLQFNKEETTAFTCENA

>DmGr64f
MKILPKLERKLRRLKKRVTRTSLFRKLDLVHERARKKAFQESCETYKNQIENEYEIRNSLPKLSRSDKEAF
LSDGSFHQAVGRVLLVAEFFAMMPVKGVTGKHPSDLSFSWRNIRTCFSLLFIASSLANFGLSLFKVLNNPI
SFNSIKPIIFRGSVLLVLIVALNLARQWPQLMMYWHTVEKDLPQYKTQLTKWKMGHTISMVMLLGMMLSFA
EHILSMVSAINYASFCNRTADPIQNYFLRTNDEIFFVTSYSTTLALWGKFQNVFSTFIWNYMDLFVMIVSI
GLASKFRQLNDDLRNFKGMNMAPSYWSERRIQYRNICILCDKMDDAISLITMVSFSNNLYFICVQLLRSLN
TMPSVAHAVYFYFSLIFLIGRTLAVSLYSSSVHDESRLTLRYLRCVPKESWCPEVKRFTEEVISDEVALTG
MKFFHLTRKLVLSVAGTIVTYELVLIQFHEDNDLWDCDQSYYS

>DpGr64f
MKFLPAKLERKFRRLKKHSRSSLTRKLDVMHESARKKVIEENCDAYKNQKQSEYECRKRPTKFPGGTRETF
LSEGSFHQAVGRVLLVAEFFAMMPVKGVTAKHPGDLSFSWRNVRTCFCLVFIASSLANFGLSLFKVLNNPI
SFNSVKPIIFRGSVLLVLIVALRLAQQWPTLMMYWHEVEQGLPQYPSQVGKGQMGHTIRMVMLVGMMLSFA
EHLLSMISAIHYARYCNSTSDPIKNYFLRTNDEIFYVTSYSTALALWGKFQNVYSTFIWNYMDMFVMIVSI
GLAAKFRQLNNDLRNFKGMHMAPSYWSERRIQYRNICVLCDKMDDAISLITMVSFSNNLYFICVQLLRSLN

TMPSVAHAVYFYFSLIFLIGRTLAVSLYSASVHDESRLTLRYLRCVPKDSWCPEVKRFSEEVISDEVALSG
MKFFHLTRKLVLSVAGTIVTYELVLIQFHEDNDLWDCNQSYYS

>AgGr15
MGFAVSENHDTKPLIQPTWHTCLKRWCASWLHWPRVQRRASREDWLFNGTFHEASRGVLMMAQLFSIMPVC
GILAKDPRKLRFSYTAGRTFYAYFCAIGIGFLATMSVYFFASKRYHFQKMVTAFFYCYNLYAMYRFGRLGQ
RWPALMVKWARVDDSLPPQKGLFERAVLAYRIKLCSIMVMALSLSEHLLSIVAAVHYSNNCPAVHDPYEAF
FKSNFAFVYYYFPYSTWRGFLTKFFNVICNFMWSYVDLFVIVISMGLSHAFRRINAHLFLHKREKMTEQFW
GEQRQKYRNVCDLVTTVDDHISAITMLSISNNLFFICVQILNSMNSRPTLVHTVYFWFNLIILIGRTLAVA
MFAAEVNDESKRPIEVLRTIPREGWCLEAKRFAEEVTTDTVALTGLKFFSMTRQLVLNVTGAIITYELVLI
QFHKDEASDVDLCKLKRMDTL

>AaGr4
MDSRFNGELVPVQSKSRAFLKHFKYPKRATRENWIHDGSFHDAVSGLLITAQLFSIMPVCGIGQKDTTKLH
FSWKSKRIFYSYAACMGTAFLAVTSTIRFVDRNFNFSRLTGVFFYFYNLYGMYCFVRVAQKWPVLMQKWFN
VEQLLPQSSNIIERGKLANKIKLISILVITLSLMEHMLSIVAAVYYTPNCPNIKDPVKMFFKSNFLFVFYY
FEYSEIRGFVVKFINVISTFVWSYIDLFVIIVSIGLSHTFRRINNHLMNHKREKMTEQFWGEQRQNYRNIC
DLVRFVDDAISIITMLSISNNLFFICASILNSLNTHPTLVHTVYFWFGLAFLIGRTLAVSMCTAAVNDESQ
RPFEVLRAIPRDGWCVEAKRFAEEVINDTVALTGMKFFNMTRKLVLKVTGSIITYELVLIQFHQDETADYD
LCTFRRT

>CpGr4
MARKLVQDRLFRRRTNKVTRFNNDVAPSVERKSHFVTELNLFKRASREDWIRDGTFHDAVGGLLATAQLFA
VMPVCDVTAKDPRRLHFSWFSKRALYTYVGLAGTAFLAMNAIIRFLMKNFNFNRLTTVFFYSYNLYGMYRF
LLLARKWPKLMQSWYDAEQTLPQLGNVVDRGALAWKIKMISLLVITMSLTEHLISIIAAVYYTSDCPDIGD
PVDFFFKSNFVFIFHYFEYSKVRGFFIKYINILCTFLWSYIDLFVIVTSIGLSHTLKRINEYLMKHKRESM
TEKFWGEQRQNYRNICDLIQKVDDVVSIITMLSISNNLFFICVSILQSLNSHPTFVHTVYFWLGLIFLIGR
TLAVSMYAAEVNDESKRPIEVLRTIPRDGWCLEAKRFAEEVVNDTVALTGMKFFNMTRKLVLKVTGSIITY
ELVLIQFHQDEPVEVDLCKMRSF

>AgGr16
MGLQKIYPAEKSNNSTEMLSKTVRILDKDFYHPPNRPRFINGGGYFCRSIRPVLIVGQMFGLLPLDGVWCG
RWWSIHWRLLSWRNLYALFVQLGALIMACFSFATFWYSGVEFAKIMSWWFFTLNLLISINFAVLARSWPQL
MSRWVQLEQSLPDQPRLSAACRRNARQVGLVATVLLTSGLIEHVLSKPAGLHRAYRCPIPNLLEAHYKQAF
PEMFSFVPYNPYIGFLAQTITSLLTVYWNYVDLFLISVSVGLRTNLAQVNDVIASSEKLYHRGIFWKDQCT
HYRRVLGLIRHVNNHIGVFIVISYASNLFFICVQLVNVFQQNSSFIVTSYFWYSLFHLIGRIVAVSLYGSA
IHDEYCRTRTLFYNLPDEYGYTDEVQRFHRQVEHDSVALNGYGFFYLTRKLILKIAATVVTYELVLTQVNE
AEAKNGDDNPCT

>AaGr5
MSKTREYRMMFFTLESVTITLTVYQKIIALWEFYRQRQVNLDQEGSYLAVFRPVIILGQVFAIFPVVGYGV
ALAERIQFKWCSLRMLYTMMFQLGGAIMSGFSLATFWTTGVEFSKILSWMFFTINLSITIGFTVLARRWSA
MMTEWENTEQSLPFRPQLTASNRRVRRKVITIMVTLMLSALFEHALAKPSGLYRAYKCGIKDLLEAHLMQA
FPEMFSFIPYDIYVGFVAQVVTSVLTFYWNYVDLFLIVLSIGLRQSVRHVNEIILNSKAQYHSDTFWHDYR
KHHQRVCNLVHIVGQNVAYLVVISFANNMFFICIQLIGVLKPYPGIIVAIYVWYSLAHLMTRMVMVAVYAA
AIHDESRRILPTFRTLPTQYYSKEVQRFHQQMENETVALSGFRFFHLTRKLILKISGTIVTYELVLLQVND
AEEKNGDQNPCT

>CpGr5
MFTTSPRSDNLILVRPKGASLEKEFYQQRRTVKFDQEGSFLAVVRPVIILGQVFGIFPVVGYGAAQADRIR
FEVCSLRMFYSVLLQLGGATMSGFSLATFWTTGVEFSKILSWMFFTINLLITTSFTVLARRWPELMKEWEN
TEQSLPDQPLLAASNKKLHRKVVTTMSVLMVSALFEHALAKPAGLYRAYRCGIQDLLEAHLMQAFPEMFSF
IAYDIYVGFVAQVVTTILTFYWNFVDLFLITLSMGLRQNLVHMNQIIMSSRGQFHSELFWYDHWKHFQKVC
ELVHLFKRKVAYLVILSFTNNLFFICIQLIGVLKPVPGLIVAIYVWYSLAHLMTRMIMVAMYAASIHDESR
KLLPMFRTMPTQFYNKEIQRFHQQMENDTVALSGYGFFHLTRKLILKVAGTVVTYELVLLQVNDSEEKNGD
QNPCT

>DmGr61a
MSRTSDDIRKHLKVRRQKQRAILAMRWRCAQGGLEFEQLDTFYGAIRPYLCVAQFFGIMPLSNIRSRDPQD
VKFKVRSIGLAVTGLFLLLGGMKTLVGANILFTEGLNAKNIVGLVFLIVGMVNWLNFVGFARSWSHIMLPW
SSVDILMLFPPYKRGKRSLRSKVNVLALSVVVLAVGDHMLYYASGYCSYSMHILQCHTNHSRITFGLYLEK
EFSDIMFIMPFNIFSMCYGFWLNGAFTFLWNFMDIFIVMTSIGLAQRFQQFAARVGALEGRHVPEALWYDI
RRDHIRLCELASLVEASMSNIVFVSCANNVYVICNQALAIFTKLRHPINYVYFWYSLIFLLARTSLVFMTA
SKIHDASLLPLRSLYLVPSDGWTQEVQRFADQLTSEFVGLSGYRLFCLTRKSLFGMLATLVTYELMLLQID
AKSHKGLRCA

>DpGr61a
MSKAPDSILRRLKVRRQKQRTILAMRWRCAKGGKEFKELDTFYRAIRPYLCVAQLFGIMPLSNVLSRDPQD
VKFRLRSVGMCFTGLFLLLGGIKTVMQANILFRTGLNAKNMMNLVFLIVGIVNWLNFTGFARSWSKLILPW
SSLDILMQFAPYAPSKHSLRSKLRLIGCVVGSLAVVDHLLYYASGYYSYHMHIFHCHTNHSRLSFGSYLEK
EFSETFELLPYNMFSVCYGFWLNAAFTFLWNFMDIFIVLTSIGLAQRFRQFADRVLALQGRQVPDTLWYDI
RRDHIRLCELASLVDESMSNIVLMSCANNVYVICNQALAIFTKLRHPINYVYFWYSLLFLLSRTSLVFMSA
SKIHDASLLPLRTLYLVPSTHWTEEVQRFVSQLTSEFVGLSGYRLFYLTRKSLFGMMATLVTYELMLLQMD
AKSHKAGLPDLCA

>DmGr64a
MKGPNLNFRKTPSKDNGVKQVESLARPETPPPKFVEDSNLEFNVLASEKLPNYTNLDLFHRAVFPFMFLAQ
CVAIMPLVGIRESNPRRVRFAYKSIPMFVTLIFMIATSILFLSMFTHLLKIGITAKNFVGLVFFGCVLSAY
VVFIRLAKKWPAVVRIWTRTEIPFTKPPYEIPKRNLSRRVQLAALAIIGLSLGEHALYQVSAILSYTRRIQ
MCANITTVPSFNNYMQTNYDYVFQLLPYSPIIAVLILLINGACTFVWNYMDLFIMMISKGLSYRFEQITTR
IRKLEHEEVCESVFIQIREHYVKMCELLEFVDSAMSSLILLSCVNNLYFVCYQLLNVFNKLRWPINYIYFW
YSLLYLIGRTAFVFLTAADINEESKRGLGVLRRVSSRSWCVEVERLIFQMTTQTVALSGKKFYFLTRRLLF
GMAGTIVTYELVLLQFDEPNRRKGLQPLCA

>DpGr64a
MEGPALNARKTGPKRRHESLLRTLAQPANTVPKLADKTHLEFNVITSEKLPEYARLDIFHRAVYPFMFLAQ
CFAVMPLTGIREPNPRRVRFTYKSLPMLVTLTFIAAALMMELAMLKHLLQIGINAKNFVGLVFFGCVLLAC
VVFIRLARRWPPLIRYWTRTELVFTRAPYEMPKRNLYRRVQLAGMMIIGLSLGEHAMYQVSAILSYKRRVN
LCSAAANITAVTSFEDYITLNYDYVFQWLPYSPIIASLILLINGACTFVWNYMDLFIMMVSKGLAYRFEQI
TARIRHLEHEEVAESTFIEIREHYVKMCELLEYVDSSMSSLILLSCVNNLYFVCYQLLNVFNKLRWPINYV
YFWYSLLYLIGRTAFVFLTAADINEESKRGLGVLRRVSSKSWCVEVERLIFQMTTQTVALSGKKFYFLTRR
LLFGMAGTIVTYELVLLQFDEPNRRKGLLPLCA

>AgGr20
MIEVGFGFRMVPGPVRSSNATRPTRSRWHWLRKRGNVTFVRPAAEIDDRKERFYLAIASVLRWARLFGVFP
LSNITATDPSAFRFHYFSPYIVLSALSIVGGLFIMAAALVRLNRVGINAMNIAEPIFFGMCTLLQLLFVRL
AQAWRGFMVYWAEREEMFFARPYGAINLRRKVIGLAVCILTSALVEHVVYVINQAYNVYQESLTCQYNVTN
PLKLYGTLTFGSVYQSVPYHHLLTMYLLYTTISLTFIWTFTDLFIMLVATGIACRFGQLNKRIDSNLQNGS
EAFWGEMRTHFVGLIELVERTNRIVGPLLIASCANDMYFLCLQTLNALEDKPYDINDWYFRYSFTFLILRT
SVKLWFAADVDENSVRTHKLVQKIRSEHYNDELEILRICSSGGVSISGMGFFTITRRIFLTMAGSILTYEL
VLMRFHRSSKGVGEDLPCGYID

>AaGr6
MIDPLWRDRYILWKNSKILQPDEEPVNNEENVSEYDFFHIAIAPVLRFSQLFGVFPLNSVMNRLPGNMLYK
SVSFATALSMMAIFGGYAVSLLSLKRLARTGLDAINMAEPFFFAVCATSAVLFWALAKEWQFVVTVWSETE
RVFLRKPFRGKALRSSIRRTAFVVLTLALAEHIFSVANNIANLRREVHHCNWTISSPVKYFCLKTFSSTFD
SIPYNLPVALYNEYVVVAMTFAWNFVDLFIVLVSIGLTTRFTQLNIRISERIQSRKGTTEDFWEQIRIQYV
SLCDLVVLLNRSINRLVFVSYANDLYFICLQIMHATLEQPFLINRVYFFYSFSFLLLRTFLMFWYSSQVQD
ASHQPCRLILRVPNHEYCDELQRVQMYSRRGVSLTGMGVFLVSRRIFLTIAGTIITYELVLLSFRKRIMDE
PDDNNDVSCEPLHLD

>CpGr6
MNYDPYWRNPNILWKKSSILAAEPFTVAEYDKFHNEATGSDHFHTAIAPVLTMSQLFGVFPLQSVRNRAPE
SMTFRTVSVTCVVSAISIFGGYAISLLSLIRQGRALNAINIAEPFFFSICATSSVIFWQMAKQWQAIIVTW
SRTERKFLHHPFRKTVLKWKIRSVATVLLLLAFAEHLLSVANNVSNLRREVDYCNWTIRDPVKFFCVRTFS

TAFHTVAYSLPVAAYNEYIIISMTFVWNFVDLFIMLVSIGISARFDQLRDRVFERMNSRTPTTEHFWEQIR
ILYVALCELAGTINRAISKLVFVSYANDTYFICLQIMNASQEQPSAVNKVYFCYSFVYLLLRTFLMFWYSS
EVQHASHATYRLILRVPNEEYCDELQRLQMYSKCGASLNGMGVFFVSRRILLTLTGTIITYELVMLSYRKD
SSEQHSSKLSCDPW

>CpGr7
MHTYPYWRNPNVLWKKSSILAAKPFTVVECGKPQKGASESDHFHTAIAPVLTLSQLFGVFPLQSVLNRAPE
SMTFRTVSVTCVVSAISIFGGYTMLLLSLVRLGRDLNAINVAEPIFFGICATSLVIFWQLAKQWQAIAVAW
SRTERTFVRYKSNLKGKIRFVAAALLLMALAEHLLFVANNVSYMLKEIDYCNWTIPDPAKFFFAKTFTTTF
HTFAYSLPIAIYNEYIIISMTFVWNFIDLFIMLISIGIAARFDQLRDRVFDRINSWTPTTEHFWEQIRIQY
VALCELAASINRAISKLVFVSYANDTYYIVLQIMNANQEQPSVVNKVYFWYSFAYLLLRTFLMFWYSAQVE
HTSHGMCRLIQLVPTAEYCDELQRLQMYTKCGASLNGMGVFSVSRRIVLTLTGTIITYELVLLSYREGSSD
QHSTTVSSCEPWQLT

>AgGr21
MCVRSNPDKENRAPVLESVRTLATSMCSSVTTTAMKLAAKGYTLRQRVRQKLCPKIVDDDRDTFLRGIRPV
VILGQMFGIFPIYGVTRNDPKRFRLKWFSLRVILNLTVVVTALLQAYYEYGRLKAIGINAKNVSSLIFFID
ACLINVLFLNLATKWRSVAMKWDEVDDTFNRPPYHMQSWSLRKRLGVVSFTLVFLAAVEHILSIVSNVHNQ
MVEIKYCNWTEPNYFQHYSLRRFANIYLNFPYNSLSAVFFTYVSSALTMYWNYQDIFIIMISIGLATRFQQ
INNYLKILSDGVLIPGEDFWIRVRTNYVAVCELLDDVDRAISWTMLISCATNLYYICLQILHVSKKLANTV
EDAYYGFSLGFLIVRTVIVFLSAAHIHDCAKKPLDIIMKIPNVGWCVELERFSTQLKSEKVALSGMGFFSL
TRQLLFSMAGTIVTYELVMLKFDQESESKGNIPLCTKFRRFEHVSV

>AaGr7
MQCIFIVHSFIVLTDNFQKDHYKLNSEKFYYINSVYILTGQLFGIFPLSGVFDKDPNRIRLVWPTVRVALD
LIVLGAGIANTIAECMRLRMVGVNAKNINGLIFFVDGCIINVLFLLMATKWNRVAVKWDSVDRIFLTESYR
IESKWTLKRRLWTATALLLGLACCEHLLATINNLNDQWHEIEHCGWKENITDTFRHFSLRKFSNMYSIVPY
STVSAVFFSYVSFALTLYWNYLDVFIILISIAIATRFDQINTHLRTLAGGGVLIPNEPFWIRVRTHYVSLC
ELLDEVDQAVAWIVLISCATNLYFICLQILNVSQKLRYPMNDVYYWFSLLFLMGRTATLFLCAAHIHEAAK
RPLDIVAKIPNNGWSVELDRFSSQLKSETVALSGMGFFHITRQLLFSMAGTIVTYELVMLKFDKESEGKGY
IRPCSFFDIEKKWLT

>CpGr8
MVASDKALKEQPPVENFPWSLHDTYHEAVRPLLIIGQIFGMFPVTGIFNRNPCKVHLEWISVRVVLNLVIF
GAALVNAVAEFLRLRQVGANAKNINGLVFYIDCGTISVLFLKMATSWNQVAVKWAHVERIFLEETYRVESW
WTMKRKIRIVAGMLLLGAVAEHLLSIVNLANDHRFEAINCGWQHNITDAFRHFALRKFSNIYIHFPYSTAS
AVFFLYVSFALTLSWNYMDIFIILLSVAIASRFNQINAYLETLAAGGVLVPNEPFWIRVRIHYVALCELLA
KVDRTMSWLMLVSCATNLYFICLQTLNVISQKHPHVMNDLYYGYSLFFLIVRTVTMFLCAARIHEAAKKPL
DIVSKIPNTGWCVELDRFSTQLKSETVALSGMGFFHITRQLLFSMAGTIVTYELVMLKFDRESEGKGYIPP
CSMFDIERQWLPSH

>AgGr19F
MNFHNTIRPILVIFLLFGQFPLYGVLTRKPLRWHFRWCSLQTVLSLGLIHVGLFLCFVEYDRLKAIGVNAD
NLIGPLFYLDVIIIMLLLLRVAYRWPTVVPKWEQIESLEVMQYNARQQNARSCRRIRAIALLLIVLGFAEH
MLSIGKTVNARVYEARTCHWNYSNLPEYYALRTYGFFFRRVSYNFPSFIFLEYANTALTMAWTVQDVLLIM
ISDSIAGYFKRINSRIQFYTTVQVVAREKFWSEIHSDYVMVCELLEHVMSICSPLLVVSCGTNLYLICYQL
FHLVDRTDDFIIVTVFTYFSLFFIILRTFLTMHYCSAVHEVARKPLKLFRRVPTSNWCSELERFYSFIRKS
SIAINAMGLFRLTKKTMLTMLGAVITYELVMLHFAQTTANQGIVRACSPEQFLFQPKMQITTN

>AaGr13P
MPREETFNTAMRPVLLIFQLIGVFPLCGILRKDSRELRFQWFTLKALLSVVIILIGLTMSYVEYEZLDRVG
GVNAQNIIGIVFFVDTVISTSLLVILAQKWHKLAVHFDEIHRIFSISDEKIIKGIHFPIRFTAGVILTCGI
LQHMSSKTADVYNQYHEAEYCGWEMKNFPYYFASRNYSFIFKHIPYNILILLIFEYAATVLTLSWSCHDLM
IILTSFGITFYFRKIYEKILPFHSGVMIASEKFWMEIRSHYVILCELVKATNGSLSLIIIHSCGKNLYLMC
YELINIARKDESILSSVHHWCSLINLTIQTTMVFYSASMMHEIAKAPLAVCNRIPNFGWCSELERYIGQLK
SDRVAYTGMGFFHLTKRSILAMAGSVVTYELVMLKFAEDTEGVGDVVPCSNLAFSKD

>CpGr16

MDSLNNALRPVFVVYQLMGTFPVGGILQRSSVGLRFRWLSVQFLFSLTLIIVGLVMVYIEYERLERIGANA
NNTIGILFYVDTVLIMALLANLARKWRPLALEWERVDQEFLAKETGEAKRSLRKAVWFTSGVMIVCGLLEH
LLSKTAGIINQSHEAKFCNWEIQSFAHYYASRQYAFIFKHIPFNIPVLAFFEYCNAALTMAWTCQDLLIIL
ISMALSHRFRQIYAQVQPFSSGIVIAAEKFWSDIRAQHTLLGQLVRDTNRLLAPLIIASCGTNLYLICFQL
LNISRKQESLASSVNHWYALCYLIVKTNLVFYHTAMVNETARAPLAICRRIPNIGWCLELERLVDQLRNER
VSLSGMGFFHLTKRTMLAMAGTVVTYELVMLKFAEDTEGIGDVKPCSRLAFSKDT

>CpGr17
MNLNRFACYFIFSIKTFRMSQSCLKLSFMPRKSRSPSSAMDSLNNALRPVFVVFQLMGTFPVGGILQRSPI
GLRFRWLSVQFLFSLTLIGFGLLMVYIEYKRLERIGANANNTIGILVYVDTVLIIALLVNLARKWRPLALE
WERVDREFLAKETGEAKKSLRKTVWFTSGVMIVCALLDHLLSKTADIINQSNEARFCNWEIKSSAHYFASR
QYAFIFNHIPFNIPVLAFFEYYTAALTMAWTCQDLLIIILTGMALSHRFRQIYAQIQPFSSGIVVAAEKFWS
DIRAQHSLLGQLVHDTNRLLAPLIIASSGTNLYLICFQLLNISKKQESLASSIHRWFALSYLIVRTTLVFY
HTAMVNETARAPLTVCRRIPNIGWCLELERLVDQLRNERVSLSGMGFFHLTKRTMLAMAGTVVTYELVMLK
FAKDTEGIGDVKPCSRLAFSKDT

>AaGr8P
MTEGGFHCIMRPIIVPVQFLGMFPIFGAAZPSSSALNFRWTSCRTAWSGIIVCCSLLMATAEMRRIARTGI
NFSNIIGTYFFVDTAIIVCLTVLMASGWRRFLHEVEKLELIWTRWPYNLRRDNLKLNCWILTMFWMFFATV
EHCLASSVQVYIQYQESLRCNWIYSNAMKNYAHRNYAYIFNWMPYNVPVLFFVRYVTYSVTMAWTYQDILI
MVISAYVLTRYRQFFWRIEVACNGTVLPTESFWVEIREHYVIISEFLVHVDKMYSPLVLSSCCNDVFLICY
LLLHSLKPHAYAISFVYFWYTLCFLIGRTLTTLWMAAELNREKRSALRVVQRISSDGWCTELERYYLQLRA
EVGALSGSRFFYLTHQTTFTIVAVIFTYELVMIKYSRMTAADVGIPENCSAMAFSQD

>CpGr9
MFKYSSTSSSTRPVLSNNQRDTFHSIMQPIILSAQCLGLFPIVGITEKTTSALQFRWFSLRTVLSVTVMTS
AFLVAIAELRRIARTGVNFSNIINPYFFADTAIIVGLLIRLGRRWEHLMVQVENVERILLKPCYNLGQVHL
KRNCWLAAGSWLVLATTEHFFATTSFVNDQRMESLRCNWTYTNAIENYARRNYAYVFNLPWMPYNIPVLLY
LRYVTFSVTLAWTYQDVILIVVCIYMNTRYRQFFTRIEVAASVERPPPAESFWGEIRYHYIALSDLLQSMN
YELSALVINSCSNNIFLVCYILLHSLGPKQSTLSFTYFWFAVCFFVGRTCTVMLTAAKFDRTLKRALQVVH
RIPNEGWCDELSRYYHHLRIEKVSLSGKRFFYLTRRTAFGMFSVIFTIEIVMIKYSKMARNSGVPADCSTL
AFSQD

>DmGr64b
MPQGETFHRAVSNVLFISQIYGLLPVSNVRALDVADIRFRWCSPRILYSLLIGILNLSEFGAVINYVIKVT
INFHTSSTLSLYIVCLLEHLFFWRLAIQWPRIMRTWHGVEQLFLRVPYRFYGEYRIKRRIYIVFTIVMSSA
LVEHCLLLGNSFHLSNMERTQCKINVTYFESIYKWERPHLYMILPYHFWMLPILEWVNQTIAYPRSFTDCF
IMCIGIGLAARFHQLYRRIAAVHRKVMPAVFWTEVREHYLALKRLVHLLDAAIAPLVLLAFGNNMSFICFQ
LFNSFKNIGVDFLVMLAFWYSLGFAVVRTLLTIFVASSINDYERKIVTALRDVPSRAWSIEVQRFSEQLGN
DTTALSGSGFFYLTRSLVLAMGTTIITYELMISDVINQGSIRQKTQYCREY

>DpGr64b
MPQGETFHRAVSKVLFISQIYGLLPVSNVRALDVEDIRYRWLSPRIFYSALIIALNICEFGAVLNYVGQVA
INFHNSSTLSLYVVCLLEHFFFWRLAIQWPSIMRSWHSVEQLFLRVPYRFYGEYRMKKRIYIVFAVVMLSA
LAEHCLLLTNSFHLSNMERTQCKNNVTYFESIYRWERPHLYMILPYHFWMLPFLEWINETIAYPRSFTDCF
IMCIGIGLAARFHQLYRRIAAVHRKVMPAVFWTEVRQHYLALKRLVRLLDAAIAPLVLLAFGNNMSFICFQ
LFNSFKNIGVDFMVMLAFWYSLVFAVVRTLLTIFVASSINDFERKIVTALRDVPSRAWSIEVQRFSEHLGN
DMTALSGSGFFYLTRSLVLAMGTTIITYELMISDVINQGGIRQKTQYCREF

>DmGr64c
MQQSGQKGTRNTLQHAIGPVLVIAQFFGVLPVAGVWPSCRPERVRFRWISLSLLAALILFVFSIVDCALSS
KVVFDHGLKIYTIGSLSFSVICIFCFGVFLLLSRRWPYIIRRTAECEQIFLEPEYDCSYGRGYSSRLRLWG
VCMLVAALCEHSTYVGSALYNNHLAIVECKLDANFWQNYFQRERQQLFLIMHFTAWWIPFIEWTTLSMTFV
WNFVDIFLILICRGMQMRFQQMHWRIRQHVRQQMPNEFWQRIRCDLLDLSDLLGIYDKELSGLIVLSCAHN
MYFVCVQIYHSFQSKGNYADELYFWFCLSYVIIRVLNMMFAASSIPQEAKEISYTLYEIPTEFWCVELRRL
NEIFLSDHFALSGKGYFLLTRRLIFAMAATLMVYELVLINQMAGSEVQKSFCEGGVGSSKSIFS

>DpGr64c

MKQVATRNTLQHAIGPLMVVAQFFGVLPVSGIWPSSPAEKVSFRWFSLSFLAAVGIFVFSIMDCVLSSKVV
LDHGLKIYTIGSLSFSVICIFCFGVFLQVSRRWPHLIQRTAECEQIFMQPGYECCFGRRFSRRLRLWGVIL
FVAALCEHCTYVASALYNNHLQIVECKLNVNFWLNYFQRERQQLFLVLQFSSWWIPFIEWTTVSMTFVWNF
VDIFLILNFRGLQMRFQQMHWRIRQNAFQRMPNEFWQTVRGDFLDLNDLLSIYDKELSGLIVLSCAHNMYF
VCVQIYHSFQSKGNYADELYFWFCLFYVILRVLNMMFAASSIPQEAKAISDTLYEIPTEFWSVELRRLNEV
LISENFALSGKGYFFLTRRLIFAMVGTLMVYELVLINQMAGTVSQKSFCDGGVGSSKSVFA

>DmGr64d
MLRSHLSVHGLQMERSVQENTLHYTIGHVLIIARIFGVLPLAGINPNGKPENVRFRWFSPYILFFVVAFTF
VIADFMLSTKIVLNDGLQLYTMGSLSFSVICIFCFGSFIKLSRRWPHIIRETALCERIFLKPCYANQEGLN
FTRFLRRWALILLVAALCEHLTYVGSAAWSNYVQIRDCNLKVGFVENYFLRERQELFSVFEYRAWMVFFIE
WNTMAMTFVWNFGDIFLFLMCRGLKIRFQQLHWRIRQNLGKPMAKEFWQEIRSDFLDLDSLLKLYDKELSG
LILVCCAHNMYFICVQVYHSFQVKGAFMDELYFWFCLLYVISRLMNMMLAASSIPQEIKDISNTLYEVRSS
PWCDELGRLSEMLRNETFALSGMGYFYVTRRLIFAMAGALMGYELVLFRQMQGAVVQKSICSRGPGSSMSI
FFS

>DpGr64d
MEWSAQSVPNKNTLHHAIGYVLVVAQFFGVLPLSGVEPSVPVASVRFRWFSPLNLLPVAALCFVLLDFVLS
AKLVIQNGLKLYTIGSLSFSVICIFCFGAFLLLAPRWPHIIRRTFECERIFLQSCYNSSIGRRFSQRLRRW
AIALLVTALCEHLSYVVSAVWSNWKQIRECHLDIDFWQNYFLRERQELFSILPYSTWFALYVEWCTLSMTF
VWNFVDIFLILVCRSMQMRFQQLHWRIRQHIGRRMSDEFWQEVRYDLLDLNDLLKLYDKELSGLVLVACAN
NMYFICVQIYHSFQVKGAVLDEVYFWFCLLYVVSRIVNMVLAASSIPQEAKQINFTLDEVPTSCWSKELER
LSEIFHNEAFALSGKGYFVLNRRLLFTMAATLMVYELVLINQMEGEEVQRSICNRGAGSSMSIFFS

>AgGr14
MLNMTPPEVTPVKRFPGVSRPPSTPSTALAEETPPADGAAERECSTHEAVAAVIFMGQLFSLIPIDGYARS
TDPRDVRMRLRSVQFVYGCVTLFIMLTLIIMLCVHTAHEPSFGVQQATSLVYYAIIVFFMVELMLLARNWS
QIMGRWYTDEAPFRTDPYRPPSRTLPFRRKVHLIAFGVMFLAFVEDTLNFVSAYRLNELHIRYCPHTAGFW
KNFFHREHPYVLRVIPYHPVVGWTIELTMRIAKFTWHYVDVFIICLSLGLQRRFVQFNERLERLDGQPQSQ
GVWRALRLDYVRLSELVTFVDERFSKLILFCCANDMFFITVQLFNSFDLKPTTVTTVYFWYSLGFLIGRCF
LMLFVVSSISRASEKPLETLRRFPSTNWNLDLRRLCDAVATSENALSGKRFFFVRRPLILAMAGTIITYEL
VLLDQVKKTPDTTRDCNF

>AaGr11
MISIIHKQGYFANPLPTNLFTNRFIVTLLAVLDSYIFQLFQKMISFQTPSAPTKKTIIVQEVNVKFKAKAK
GCTAHEALAPIILVGQLFSLMPISGYFRTPISKLKFTLKSVHFAYGCFTVFIMGAIMSMFFAFRIQRGTFG
IGATTTCIYYAVIITAMIEFIILARNWPLIMQRWTADEDVFLSNPYETGQYLPLESLVKRVAFTIIFFAFV
EDTINFISAYLLNVVHMKYCTHATDFWRNFFRREHAYIVRFIPYHPALGVAIEVVMRVAKFTWHYIDVFII
CVSLVLQRRFQQYNDRIRTFNGNQQPEEVWRTLRLDFLRLSELVTYLDTKLSRIILLSCASDMFFISVQLY
NIFDPKQTTVTTFYYWYSLLFLICRCFVMLYVTSSIYEASLKPLELLRDFATSSWNLDIQRLLDHASLKSI
AFSGKRFFFITRPLILAMAGTIITYELVLLDQVAKEQDTTRDCDF

>AaGr12P
MISIIHKQVYFANPLPTYLFTNRSIVTLLAVLDSSIFQLFQKMISFQTPSPPKKKTIIVQEVNVKFKAKGK
GCTAHEALAPIILVGQLFSLMPISGYFRTPISKLKFTLKSVHFAYGCFTVFIMGAIMFMFFAFRIQRGTFG
IGATTTCIYYAVIITAMIEFIILARNWPLIMQRWTADEGVFLSNPYETGQYLPLESLVKRVAFTIIFFAFV
EDTINFISAYLLNVVHMKYCTHATDFWRNFFRREHAYIVRFIPYHPALGVAIEVVMRVAKFTWHYIDVFII
CVSLVLQRRFQQYNDRIRTFNGNQQPEEVWRTLRLDFLRLSELVTYLDTKLSRIILLSCASDMFFISVQLY
NIFE

>CpGr15
MLFATKSAGSLKKGPILVVQEVKEESPIPNDCTAHVALAPIILFGQLFSLMPVSGYFHRTPDKLAFRVRSL
RFLYSCVTLFGIVSIVVLFLMYSIRRGVLGLSSAATFIYYTVITLALIEFMNLGRNWHWIVAYWTEQEKPF
LYYPYSTRKGLKLDKLVKCVASAVIFFAFVEDIMNFISAYKLNELHIKYCSHRDDFWRNFFHREHAHIVKV
IPYHTVVGVGIELMMRVAKFTWHYIDVFIICVCLSLEKRFGQFNSRIERFKGVDQPPEVWREIRLDYLRLS
ELVNFMDDRLSRIILMSCANNMFFISVQLYNIFELKPTPMTSVYFWYSLLFLMSRCFVTLYISASIYEASL
KPLELLRDFSTLNWNLDLQRLLDHISLKNIAFSGKRFFYITRPLILAMAGTIVTYELVLLDQVSKDQDTTK
DCNF

>AgGr17
MESPAIDDFHRAVRPFLLISQLFSLFPLGGLYGRTLQDIRFRWFGPGTVYSFYFFLSGLLTLVAHIYYSLT
VETLGTSEISNIIYYVLNLSGAIVLLAIAARWRTIMEKWRSLEENFLHPPYAERRFWSLKRVVAAIGSTMV
VLAFVEDTLHVASVYYTNLQYFKRCDNSTPFWTLFYQREHPKFFHYLPYSLPAVLLLELTHKIFLYVWTFM
DLFIIFVALGLARRYEQFYRHAAQYKGRHVMGPVWQRLRLDYGRISSLVAYMEGIMAPIIVCTTASDLYFI
FYQMYNAFQFSASLISELYFKFSLAFLIFRTLVMLLIASNIHVASLRPLDILRSVPMSCWTIDVQRFTQEL
LSGRNCLSGHGFFFLNRSVILAMAGTLITYELVMLKEVNPSSEKSDFCDGVKRLY

>AaGr9
MQAPNQHCLAQLRKWHRHQFPSDNAVMDSNSKIDDFWGAVRPIIFVAQLFTLFPVQGVFGRDLQQIRFQWL
HLRTLYSLTFLVLAALIIIAQINHTVVSTANTSMITSVLYYILNFSGSVCFLIIATKWRNIMLNWKMYEEV
FLHKPYLMKGRSLKFKVRMVGGGILLLAFVEDLLHMLSCCRTIEVYIERCDNSSSFWETFYTREHSKTFDY
ISYSLPLALLLEFVHKVYLFVWTFMDVFISVVSIGLATRFEQLFHRIEHLKGKMLPESFWAEIRLDYTKIS
NLVIYMDGVLSPMIMITSASNIFFITYQLYMSVQLDASSMTTFYYRFSLLFLILRTLVMLLTSSRVYVASR
KPLEILRAVPMSSWTTSVQRFTNEILNIENALSGHKFFFLKRGIILAMAGTMITYELVMLSEVRHSDNTQF
CDGGHRLY

>CpGr11
MNSLPMDDFWGALKPVIFVAQIFTLFPVQGVLAKDVYGINFRWRSLRTLYSLFFVVLAFIALCAQINLTAL
FTVTTGQVAGILYFGLNLTGAICFLIISRKWRAMMIRWKSQEDVYLRPPYRVYGRSLKFKIRLIGFSVIIL
AIIEDLLHVASSIKIHHKYINFCNVTGTFWELYYNREHPQVFKYVSYNLPTVLLVEFTHKVYLFIWTFMDL
FITLISIGLLTRFEQFYQRIEHLKGKSKPEVFWAEVRGDYTKISSLVTYLDEILSPMILITCASDVFFITF
QLYMTVRMKTTSITTIYYRFSLIFLIFRALLMLLTSSHVYVASRKPLEILRAVPMSSWTTSVQRFINEILT
IDNALSGHRFFYLKKSVILAMAGTLITYELVMLSEEKPLDSSNICG

>CpGr10
MISPNQDDFWGALKPVIFVSQLFSLFPVQGLFAKDASAIQFRWMSLRTLYALFFLTLAGLAICAQVNFIVG
TKVSTGVVTEQLYFTFNFCGTVCFFVIACRWKQIMMYWRKQEDIFLRPPYKPYGISLRVKIGVTGLSLIAL
SFAEDILHILSSMKVNQDYIDFCNITEPFWMIFYTREHRKVFDYVPYNWPMTLLVEFTHKVYLFVWSFMDV
FIALISIGLLTRFEQLYSRIEHLKGKPMSEAFWAEVRRDYMQISNLVAYMDRVLSPMILITCASDVYFITY
QLYKSIQLKLTSGTTFYYRFSLVFLIFRVLVMLLTSSHVYVASRKPLEILRAVPMSSWTTSVQRFTNEILN
IDSVLSGHKFFFLKRRIILAMAGTLITYELVMLSQVKATDSEIKCED

>CpGr13P
MSSLPMDDFWGALKPVIFVXQIFTFFPVQGVFAKDIYGINFRWRSLRTLYSLFFLVLGFIFLCTQINLTVL
YKGTLWEVGGILYFGLNLTGAIYFLIISRKWRSIMIRWKSKEDVFLRPPYRMYGRSLKFKIRLIGFSVIIL
AIIEESLHLASTIKVHHKYINFCNVTGTFWELYYNREHTRVFKYVSYNVLTVLLVEFTHKVYLFIWSFMDL
FITLISIGLLTRFEQFYQRIEHLKGKSKPEVFWAEVRGDYTKISSLVTYLDEILSPMILITCASDVFFITF
QLYMTVRMKTTSITKIYYRFSLIFLIFRALLMLLISSHVYVASRKPLKVLRAVPMSSWSTSVQRFINEILT
IDNALSGHRFFYLKRSVILTMAGTLITYELVMLSEEKPLENSNICG

>AgGr18
MYEKAANHFNKTMSIENPHIRKGNGYGRGNWKSFGRQYVRPCVGSFHHAVAPVLLLGQCITLLPVVNIFSA
NYRTARFKLRSFRCIYSLVYLALTGIYCTLFIRWYIRKGLNLAYFANCIYMVVVYLSAWLFFFIALRWQSI
LGAFSRCERSFLSDHYRRQSKGRFALGWKIRFTGFAIFALALVEDWLNYYSAYQSNIVQIATCNRTNVTVW
QNFYLREHPHVFHWLPFNGFTIAITEWINRCMRYTWTYLDIFIISFCYGAQFRYEQIFRRLVAVQGIACPT
NFWHDVRMDYVAVSELVQVLDAQFGHLILLACANDMYFIATQLFNGFQRRRVIANYVYFWYSLLLLMFRTI
VMLYVGSGVYAASTSPLQLLRNVPSQHWGIDLQRLTDEVASGENVLSGKQFFFLKRQLILAMAGTLVTYEL
VLLDQVKKIPDTSTDCSYF

>AaGr10
MSPSVDMKLLTLTSRRAATAVAIGKESFFPNFHLPRWSKWIGRLKGKSTFDGSFHQAVAPVLFVGQCFTLM
PVVEIFSHNPRNTRFKLLSVRFAYTAIYLIAAGFYSILACRRFILRGLNVSSFADMFYLMFNYIITVLFLL
IAIQWQKVLKEFANCERLMLKDAYTKLTERVTRFNLAWRIRMVIAGIIVLAFFEDFLNFYSAYQDNYVQMD
YCNRTEISFWENFYIRDHPQVFQYVPVNIGSILFVEWINRCLRYTWTYLDLFIISFSYAAQFRYTQIYQRL
VSVEGTHYPTTFWREIRTDYVAVSQLVAFLDEQFGHLILLSCANDMYFIATQLFNGFQRRPAFMTMVYFWY

SLALLIFRTLCMLYIGSGVHVASMSPLNILRNVPSKYWGLDLQRLTDDVASGENTLSGKKFFYLKRQIILA
MAGTLVTYELVLMDQVKQAPDPTTDCSFY

>CpGr12
MKLLALTSRHFQLPWPQPRKGSAAVSSSYHGSFHQAVAPVLFIGQCIALMPVVEIFNHNFRRARFKPLSAR
FLYSMLYLVIAGVYGVCTCRWCFVKGLDVTLFGDSVFIVVVYMTAVLFLLIAPQWHTVLKMFNECEKIMLR
DTYRKVTERYTRFNLAWQIRLIAFGLLVLAFIEDALNCNSVYKGNVVNLQFCNHSNVTFWENLFIREYPQI
WRNIPVNFGLVLIIEWIIRCMRLTWTYLDVFIISFSLAAQFRYNQIYYRLISLPSVASLPSTFWRNIRTDY
LAVSQLVAFLDDKFGHLILLACANDMLFIATQLFQGFQRRPTFATIIYYWYSLGLLIFRTLCTLYVGSGVH
VASMSSLNILRSVPSEDWGLDLQRLMEDVASGDNTLSGKKFFYLRRQIILAMAATLVTYELVLMDQIKQAP
DKTKDCSYF

>CpGr14
MKLLALTSRHFQLPWPQPRKGSAAVSSSYHGSFHQAIAPVLFIGQCIALMPVVEIFNHNFRRARFKPLSAR
FLYSMLYLAIAGVYGVCTCRWCFVKGLDVTLFGDSVFIVVVYMTAVLFLLIAPQWHTVLKMFNECEKVMLR
DAYRKVTERYTRFNLAWQIRLIAFGIMLLAVIEDSLSFNSAYQGNVVNLQFCNHSNVTFWENFYIREHPQV
LRNIPVNFGSVLIIEWLNKCMRLTWTYLDVFIISFSLAAQFRYNQIYYRLISLPSVASLPSTFWRNIRTDY
LAVSQLVAFLDDKFGHLILLACANDMFFIATQLFQGFQRRPAFATMIYYWYSLSLLIFRTLCMLYVGSGIH
VASMSPLTILRNVPSKNWGLDLQRFMDDVASGDNTLSGKKFFYLRRQIILAMAGTLVTYELVLMDQIKQAP
DGSKDCSYF

>TcGr4
MLNNSAQRINNFFKLILSLGCFLGFFPSFQMSFAWKSWTTFYTIFITSLSVIYNFIPILWVVNYHQQNISF
EDVEIIIHSAAVVFNFFILFGLAKNWNKIIDEWHNMDTTLNKNFSYPKHLKLLVTALTTLFLLFGIVVYFL
KQMAFFSKLSETEGPISIELFYKHSGKTIFWIIPYNWITATIFTFLQFNSFIVFIFTDILLISISLILANR
FQQLSKTLTKRQKIPQFHPDNASFWKNVRKDYCKLSSLLFLIDDHISLAIIFSYCLNFFSLLRFLTKLLRD
SEQNLIVKFHDYCDALNFTLRIICLTLFSSWINEASQEPVAILNSVVEREYSAEVGRLLLQIGFDEVALTG
CKMFKLNKGLFLNIVSAIVTYELIVIQYNNN

>TcGr5
MVFAELWKKIRNRGTPPKFELPTTHNCLKKVLLLSQIVGVFPLNHLNEEPEKLHFTFKSWKVLYTSLTSFG
YLFCASLSFYKAFKIGILLNQLITPLFFFHSFMTSVLFSQVASRWPLFLNEWTKIEINLLKHYQSTTDLHK
KIRYSAFGMIFVALLEHCFSMLNYVYSSKCENNSTGVEHFFKKQFHYIFTYMPYNIVFGLCLTFVSWTAAF
VWNFGDIFIIILLSMIMTERFRQINQQIQKKAEELISTPSPNTKNFIKKADYQFWRQIREDYDHLTNIVRYL
DQILSNLVLLSYSCNLTFILIQLFNSLRQMKSAGESIYFFYSFGFVIMRIVFVSIFGAFINEESQAALSYL
TSLPTEHYNEEIQRLVTQTHIDSAALTGHNFFRLTKGLVLSVAAAIITYELVLIQFNQATLNKYMQANETI
CF

>TcGr6
MKVLSNDLFHHRVKWILLLGQTFGLLPVNGVTSEKCNLTFSWYSKKVFYSKIIISGSIFMTTTSFYRILNS
GYNLTTFGSFIFNANSAIEGIIFFNLAKSWPQLIEKWSRVEMALDNWKNDKSLKRKFYLTICTIMSAAAVE
HILSIVNTCSQIPSEVEDKYTTYFLNTYPHLFNFFEFSVPLAIFAVVMNLCNVFVWNFLDAFLIIISIALT
EKFRQVTAKVIVAHNEKIHLKHHWVKLREDYNQISILCKTVNKKISTLIIVSFGTNMFFIMSQLYWSLSIV
KTTPVESIYFSFSFGLLVLRTIAVTLFASNINDESKKSMNYLLSLSSDIYNSEVERFAFQIHSQPVALTGN
DYFTITRGLLFSMAGSIVTYELFLIQSNEAVSY

>TcGr7
MRLIRKMKSDLVLTIEASDAVLAYPQPSFHQTFSFVVIFGQFFGIMPLHGVSRKNVQEIRLEWKSFRFVYA
VYNIFGAFVMGLFCILKFALDGLMLDKATMSFYVLNFFGSIQFIIISKHWVTIMKEWSFMEMSMRNYGSSI
NMKKRFVVMTSVIMTLALVEHLLFIANAFITSQSCENATLYSGDEMYFRVAFPSVFTLIDYSLWKACFVEI
ANILSTATWNYTDLFIILISCSLASRFAQINHRLKNNKLLHEKFWREIREDYNKLAHLTAVVDRNIAALVV
ISFVSNAFFICVQLYNSLKIRVGTVETVYYFFSFGFLVARTIAVTLYGAWINDESRKPLEILHSVPSEHYC
EEISRFIQQINSSPVGITGSKFFILTRNFLLKMASTIVTFELMFLQFGPLINTNINHRSTDCFM

>TcGr8
MTTTTHSSLRFILIAAQIFGMFPVSGVAKKDPTFLKFKWTSKRTIYSIIFALAAVVNTIIFLVHRVSLGRL
KFADWVTLVFFSVTFLIVVLLLQIAKHWPSLMKKWTQVDEAMSGYGFPPKLERKLRIIFAITVVASLVEHG
LFIGVEYMSCRGNNLSEALDRFLMFHYDYVFALVPYHVVLGIILEIVNIFSTISWTFMDLFIILVSLSLSA

RFKQVAKYIKFLVERNVLNKNSWQRARQDYTRLTNLCKDLDEVMSSTILLSFGNNIFIILVHLYNSLQKPL
EFGYLDEIYYLYSFICLLVRISAVALHAATINTESKRPIYLLSTIPHHRYNLEIDRLLLYTKYETAALTGY
KLFRITRTLILKITLAIVIYELVLVEYLKVESY

>TcGr9
MKENTTQNCLYFMLVFAQCFGMLPVTGISEENCKNLKFKWSSKRTFYSLTFALVATANTVFFLIKMIRGKR
NEFQEWVALLFFFVTVITVVIFLDVAKKWPQLMKKWTEVDTAMNSYGFPIALSKKLKTTTLLVISAAIVEH
GLFVAVASAPCEGKNWSELFNNYFKMRFDYVFDVIPYHFFWGIMFQVLNIFSTVSWNFMDLFIILISLCLS
ERFKQVATRTNQLAAKEVTDERIWEQLREDYTRLTILSHTVDKTLANTVVLSFANNLFVILVQLFYSLQAP
PRFGILNKLYYVYSFAFLIIRMVAVALNAATINNESLKPKYCLNTLPHFLWNVEIDRFIYQIKYSPVVLTG
HKLFKITNALVLQITVAIVTYELIMIQYVRVKWS

>TcGr10
MRVQPSNTPPEKSQLSTADTANLINKSLGKHNLQKSFAKVVILAQIFGFFPAQGILGRDFRAIHFTWASAR
VGYTIVTILGATFVTVLQLHKIFAKGLNVIEANRLFFYCSGLASGYLYLKLAMKWPRFMKDWSCVEVMMAS
YGWPAGLNRRLNVLLAVFMSLALIEYILMQTNKLVLALECNNSTSEGFDYFFGKMSYSHIFSLMDYNIVMA
LVLQFITLQHTFIWVFNDVFVMLLSTALAYRFTQVTDRTQSMSESKNKSESAWKNLREDYNRLCRLCKRVD
EEISYIVLMSFASDLLFILIQLFNSLRQMKNNLERIYFYWSFGFLIVRTVCLCLFGGKVNDESTQPMLVLN
SVSADVYNLEIQRFIHQIGTLEVAFTGKNFFSITRGLILSIAGAIVSYELVLMQFNDSLLETISEQIDSCP
VYL

>TcGr11
MFKSPTNMEKSTKIFPVPGDKNLHKSYSKMLILAQIFGFFPVQGVRGPDFRSLRFSWKSARVVYALFTLLG
TFLISGFQMQKIATKGLDLLEANRLFFFLTGVMASLLFLNLAKRWPKFVKDWCVVDATFASYGWPKGLNKK
LNTLTVVFMLIALVEHILVQTNKLVLALECNNTTAEGFTYFLGNMSFAHIFALVEYDIFKALVLQTINLQI
TFIWTYNDLFVMLISTALAYRFGQITRRIAAVASEKIKNEIIWKKLREDYTRQCRLVRKVDKEIAYIVLLT
FASDLLFILIQLFNSLRRMRNDLERLYFYWSFALLITRIVCLCLFGAKVHDESIKPLLTLNSVPTEIYNLE
IQRFIQQIGNSDIAITGKNFFSITRGLILSIAGAIVTYELVLIQFNVNLLQGELNKKGSCPVYV

>TcGr12
MKKNSKYVQNCIFYAMKRPLLVAQIFGYFPLYGTNSDPTCLKFKWISFKTTYSVFTLFVTFFIAVCQLHKM
IAVEMNILQMNYFVFLLCSILVNIAFIKLATEWPQLMKAWLKIELLVGNLGMRRNFRKKLDFIFTFITLLT
IVEHLLMELSRAIDSVACSKTVSDGIRHYYVNVTFPHLFNGLVDYSLWKALIFQISNLQTTFGGTFGDTFI
ILLSMAFATRMKQSRTKIEALVKSHVKATTPWRKIREEQCSLLYLCTLLEQKISYLVLLSFCSNLYFVLVQ
LFSALKQMGDTLQKTYFFISFGILIFRIIFVSLSAASINEESRKILILLLSTPSELYSVEVERLTNQINYK
AMAISGKNFFIITRGLILKIAGAVVTYELVLIQFNKKLLNEFDETSVQQLEILHNNTYWLC

>TcGr13
MKNIISHNTENTIHSSLKFSLKILHIFGLFPVSGLSGPDYKSLKFSWRSFKFLYSLCFFCILCLFVLTLLY
NVFFVKEATQEITNVLFYLSAAATNAVFLQLAKNWSRFIHEWHCVEVIMGSVAINHSLKKRLKIITIVILV
VATVEHLLIQCYIAVSIFGSSSFEADLRQFYKTAYSAIFTVIDFSLCKAILVHAITIRSTFSWTFIDVFIM
LTSTAFVFRLKQLNAKVEMLKNARVKNTALWKQLRYEHYRLYQLSVLIDNNMSYIIIVSFATNLYFIIIQL
FGSMKIVKGTLKTAYYLISFALLIMRLISVCLCGASVHSESSKVLPLLFSVSSSSYNCEVERFIDQVIKNE
IILTGKKFFKITKQLILQIAGAIVTYELVVIQFNLRLTEDNDSNEANLLA

>TcGr14
MLFSSAAKTRPKPKSPQHEKENVHKSLKSIIVIAQIFGYFPVQGVLSNEPIFLNFSWFSLRVVSSLVTIIT
GVVIVFGHIRLMAVGGGYSQFEMNGIIFYACGTLSCIFFLKLSMEWQSIMLKWQEVDLKMSSYGWPKNLNR
RINVTSAIFLLLEAAEHVLIQSNKLVVAIQCKGSFSKGAEHFFVNMSFPIIFDLIKPYYGAWLGVFLQIVN
TRMAFSWTFIDLFIILMSCALAARFRQINTRIRCLTNMKVTSEKSWIALNDDYNRLCHLCFLLDEKLSYII
LTSFLNNFYFIIFQVFESFYLHKATTLETVYYFISLGLLILRLVSVCFYGSWINEESKLSLDLLSYVPREV
YNKEISRLVETLKFQSVGLTGKRFFKITKQLILKITSAVVTYELIVIQFRKKMERDEKPYSRNSIC

>TcGr15
MAITVVAPQSKTKPFTQKTVYYTLNFFFVLLLFSGYLPVQGLFASPIHLKFKYFSREFFYSLYIIVVACIM
HFLRLYHYFTIKNDKITLGELIFMCTAFLSLVFFLQLARKWPRLIQKWCHLDEKFNVTYSYPKYLKLQLTL
VTSIYLLIVFGEYFYFMSVKLDGSENTFQKVVEKIYFYIFAHIPYNIAVVLVIVLPIFSNFIALSLIDVFV
ILVSITITFRFQQINELLKEQKNKNNSINFWMTVRRNYSEIGRLVSEINDTISSITVLSYGSNMILLINEL

SSFINVVVEESKCFYVYSFLVLIIRLLSVYYFSSRINAESRKPIRILFDVSSEIYNVEIKRWFMQMKLDSV
ALTGSTFFRITPGLILSIAGAVVTYELIFIQFSQPK

>TcGr16
MQSSEDGDHIQNSLKLLIKIGKVWGFFPLINSSNEQVSFKFRSCCFIYSLFVWTVLFYILLMAVYSCCVMP
NFFRYNFVHVMYSSNLFCLFTIYLRLAKKWPDIITYWKRVDQAMEPKYGYPKNLTRKVKRIVIFWACTSVV
NQIVVPFAPMYHLIVHGKLQFPLSPFAIFMHTILFLQSIIGKTTVHYADLFIVAIAISLKRRFQQITQRIV
NHKEQYKNVEFWRNIREDYDRLARLTQFLDKELSYLILFSIGFNCFWVMKLLYNVLRFGAESQTINFMFSF
GFILLRFLSVFESCTRLNNESRKPSFVIQFSHIPVDNIEINRLIQQINFDKVYFSGCQLFKIKNGIILSFA
GALVTYELTLFQYHAFYLT

>TcGr17
MKKIRIDNSRNQRKTPRKKRPQLINALLLLMKSGKYLGIFPLKRVTSFEEKYIDFKSCVFIYSSLVWILNF
VVVLLTLSDWVYTNFSVDKFVSLVFNSEIFFSLTLFIYLANNWHFVMSFWNQVDQKMEYKYNQPRGFNRNV
KLGLVVYSILSVGNHFGSPMHSVVSLFQSQNQITEVTFLPQYNDFFSKIFTIHSILGKTTMHYRDFFLIII
TLILREKFKQINQRIQDNSENISTDFWQEIRNHYDLLSHMVRHFDKEVSYLILASVGFNGFWVLLFFYKSY
TAKFSSSANLYFAYPFGYIIILRFLLLFHCGALLNEESKKPAIIIHFSEIPVLNTEITRLLTQIEFDNVFLS
GGKIFRMKSGILLSVVGATVTYELTIVQYNIFSN

>TcGr18
MKAPQSGLLLIQPQNNFHKSLRFLFVIAQVFGYFPVQGVSEEKVTGISFTWFSLRVLSSLLTAFLGIVVIF
AQIRFMKLITGYEQAQMNAIVFYGSGTLSSFLFIKLARDWHEIMTKWNQLDKALISHGWPKGLDKTLKRIA
IIFLALEAAEHFTIQANKLIVAVRCRGSFSKGFRYFAVDMSFAVVFDFVDYAHWLGMIFQLMNTRMAFAWT
FLDLFIILVSCALSERFRQINDRIQHLTDIKSKHFKEWIAINEDYNRLCLFCDYLDDKISWLILISFFNNF
YFIIFQVHLSLNLVLAPPLEIIYYLISSSLLILRMVSVCIYANRVHEESKSPMDILSVAPLEIYNQENIRL
IVTAKYQTTGLTARKMFLLTKNLIFAIAGAVLAYELIVIQLNKNQPRKQGPSIYSLC

>TcGr19
MILFPRHTEMDNTQKWSNSFKYVFFLAQCLALLPVNGKHQIYSKWKSLHILYSLFVIFVTSILLIFQIVFA
VTHEFDRNVLSSIMMKIYSICALFLNIRLGSRWQKIHKQWNQVDCVMDRRYGQLKKINLRIIAILSVYFAT
SLVNILKLQGTSNILEFELYSFVFNYVPPNYAMALIFLFVYFIYFFLSNFIDVFIASLSMAIALRFKQIRT
RLELSEKSQFDLGMTEFWLEMRRDYDRLSHLCKELDDGISGLILMSFAYNLFEVISYLFHQLMMDSQQNVA
FYFFFPYMVLRLLAVCLYTSWINDESLAPVNILNSVPSRNYNPEIGRWLVQMSFDNVALTGWKMFKVTRGI
FLGVASIVVTYELVIMQFYGFSGKT

>HvCr1
MGPTSLKRNMFFWIPVKKNKVDVAKPKVKNITTFQDALRATLIIGQVFSLLPFVGVFTNVASNVKFIKTSW
KCGYSLLSLIGQMFMAVLCVNKLAKSNVSLNGTSPVIFYVTTCVTMMLFFQVARRWPALVQHISKAEDMDP
NFDCSLTRKCNITCAVVLILALLEHILSLLSAFAGASACYTGMDTYQGFVTHFYPWVFNYLPYSIVLGVIT
QFLHFQSTFIWNFSDLFVICMSYYLTSRLEQVNRKLLAAQGKYLPEIFWRATREDYCRVTQIVRKVDEVIS
GVVFISFANNLFFICLQLFNTLEDGLKGTGECTQLNSQSKLKKIVVSKSGPLGGHEAAAYFLFSLVYLLSR
SVAVSLIASQVNSASSVPAPVLYDVPSPVYCVEVQRFLDQVNGDKVALSGLQFFSVTRGLLLTVAGTIVTY
ELVMFQFNSSTPSLNITSPTSATHIITTLAT

>BmGr6X
MLLRNYKQNLSFWTSAKKSKIHKIQSQETVTFQGSLKLVLFIGQLFSLFPVCGLLSNDANKVXFVPISWKC
GYSMLSMIGQLFIIVMCILYVAHFETTLNGTTPIIFYGVTFISMIAFIRASRRWPELIQHISKSEELDPSF
DFRLKKKCNITLLLVLVLAILEHIFSIRSAYSASQICYPHTGFYEGFVRYLYPWVFDFLPYSEELGMVTQF
LNIQSHFIWNFTDLFVICMSYYLTSRLDLVNKKLLPAQGKYLPEIFWRTTRETYCRATKLVRKVDEIINGI
LFISFANNLFFVCVQLFNTFDDSVDMVGLCYNYSERRTKPVGREPVIYLLFSLGFLISRSITVSLIASQVN
LASTVPAPILYDVPSAVYCVEVQRFLEQVNGDNVALTGLQFFSVTRGLLLSVAGTIVTYELVMVQFNQAPA
SDSFTEKLVENNISTIETFYNYS

>BmGr5N
YFAFYLSTGCNTFIFLRVASKWPTLIKHVYETQLDSYIDVKVKNKCFAAYIIFFSMSMTEHMLSLLSKFVI
TMDCLPKGSDLFESYIIRNFPWLFEFDVPYYLPIGVILQFLTLVSTINWSYSDLFIVCMSIYLTSILKQIN
KKIEMAGNSNHLPIPFWRTLREDYTRATRLVRSFDDTISSVIFLSFASNLFFICLQLYNILSNGVTSKYNL

LKEMCPNYPSGPLGGYEQIMYLLFSLSFLLGRSLVVSLVAAKVHSASMVPASALYNIPRNMYCSEIQRFLD
QVHGDKVALSGLRFFYVTRSLVLSVAGTIVTYELVLLQFSNED

>BmGr7
MVLEAHTQIQYCTAKANYCEFHAGLRHLMRLARWAGFFPVQGLSQTNPDDVRFEFRSLYALYHAITVIGQT
VMTFLAFYSFVDSNVSLSVVSNFLFYFTNYVTLVLLWRLSKNWSALISKTLEFEQSVTEIRTTRNLVSRTN
TLTYVVLIFAMVEHALSKVFNIRSVMCCLGETSLNHTVINNYFKFKWKFVFDYFSTSTTYSYFVGFIAEFL
CMQATFLWSFTDVLIMCFSIYLSSFFEDFNSTVSSFMKKASKTVPWSTLRVQYSQIVLIVKQMDEQLDYFV
LISYFTNLFFICFQLYNSLNRIYDANDVCNENMDIIATASVTYLTYYVFSFLFLVTRALLLSIMAANVHSC
AQVPQLALYEVPTADYSLDVQRFQLQLRYTTVGLSGVCFNVTRGMILRVIGTIVTYELVLIQLTKKNLDND
TSIRDYYLPKHLI

>BmGr8
MAPRSVRSMVGTSKKDMLKGGFYETVRIPLYIYRLIGILPISGLWHRSSKYNRFSLKSFYTIIYAPTIVMQ
TFLLLVHIYDLFAFFFGHQRLGRLIYHMNFYTITILIFMGSRKWKNVIKEIETIELTLPRLRNSKKALALT
KSFVFAFFVFSLAEVVLILQFTLRLTKQRHVLPGDSGLYLRSYFVYIFPYLYDHFPFSYVMGFIVQIIKVQ
GIITLNMVNCSVVILSIYLTNRLKHYNRIVFAKGSKTNNTRLKWVELNLLYTRISNLVKIIDKNLNPFVFI
SFTANLSYICAQLFYILNKLTSSRTVKITSFLEDKRCDWETVLYISISFALVVLKVLLVSIIAAEVHTTSR
EPLRLLYTLPTAEYTIETQRLMTQVYYSNLSLSGLNFFHITRGMLLGMVATLLTYEIVLLQI

>HvCr5N
AYLDKVFLWSCLYGVFGSKRFISLIWSTLILGSLVIIEVLAIWKVIRALAGVARDMSGHRSVTARLAGTIF
YSISILSLVLVSKLYYNWRTNIAGVWGKVERSVGVKIPVDRTLKCRMTFVAGLMTFFSIFEHAMSILSSVG
LDCPPSLILKRYVLVSHGFIFMGQDYSEWFAMPLVIISTIATLLWNFQDQVIVLISMGLTSRYRRLNECLA
KVCELEKQHKDSDKKIEAVKVYTWRKIREAYVKQAMLVRKIDVALGGIVILSCSCNFYFICLQMFLGITQG
LSSDLLSLIYYVISLAWLCTRVISVVLAASSVNTHSKLALNHLYNYETHCYNVEVERLQDQLTKDYIALSG
MGFFYLNKTILLQMAGAIVTYELVLIQFDDQGNDALNATKI

>BmGr4
MDKDKFQEFLPTMSRIFSMTRYFGVSTCKPSIAFGWTVILLLMLLAIEVGAIWKIVRLLGGWAVHSTDSRG
FTARLSGCIFYGNALLSLILSIKFVSSWEQLSERWSRTETDPGLRLPSDSRIKRRTVLVSAFVMTCACVEH
MLSMMSATGFDCPPEEYTERYILSSHGFLVQNDEYNLWLAIPIFIMSKLATALWNFQDLIIILISMGFTSR
YNRLNTYVHRVVMLERNLKEGAQVSSENYMRFQIWRRIRQAYVRQAALVRLVDDQLGALVLLSNVNNLYFI
CLQLFLGINSKDRGSFINRLYYFISLGWLMFRACGVVLAAADVYIHSKKALISLYLCPELAYNLEIKRLKY
QLKNDEVALTGMGLFSLNRELLLEVAAAVLKYELVLVQYDK

>AmGr1N
SEPVAFSANSFNPKTDSLHASMRPIIMLAQFFSLFPVSGVNSPDSSYLRFTWRSPKFIYCTISFLSSSIMT
IFNVLRIVTTGISSIKMTTFVFNGTNLIASFLFLKLAMRWPCLMVTWEKLEKELSQRHRKISKISLSMKFK
IVTIVVMTFALVEHSLSIIHGYFKAKECIEFHREQSILGVYFQMQFPQIFSRTSYSLWKGILVDIINILST
FSWNFVDLFLILISIALTDQFRQLNSRLYSIRGKAMPEWWWAEARSDYNHLATLTRQLDSHISIMVLLSFA
TDLYFICIQLLFSFNPMRGIIEKIYFGFSFGFLLARTTVVSLCAATIHDESLLPAPILYSVSSSSFSTEVM
RFLSQVTTDNICLTGMKFFSVTRSLVLTVAGTIVTYELVLVQFNTTQQTDASNATIVCEVK

>NvGr1
MSQLHFRSSKIDSSPKRGRYESLKMDSLPADVSRPSASNSGANLVESTRSFHCALRPIIILAQCFAVFPVS
GVRSPDATHLKFTWRSFKILYCCLSTLGSIVLMFFSVYRLATTTISSNKTSNLVFSLTAGITTLLFLKLAR
QWPSFAVSWENMERELATRHNPRRSSGINLATKFKILSVVVMVFALVEHTLSILSGYVSAVECASLRGDKD
IMATYFALQFPQMFTDSNYTLWKGLIVQFVNFLSTFSWNFMDLFLILVSVALTDQFRQLNQRLYSIRGKAM
PEWWWAEARIDFNRLATMTRRVDSQISDIVLLSFSTNLYFICIQLLNSFKPMPNAIQTVYFCFSFGFLLSR
TSAVSLYAATVHDESLLPAPILYSVCSASYSTEVRRFLTQVTTDNISLTGMKFFSITRSLILTVAGTIVTY
ELVLVQFNAVQAEHQQSESNITKVCEVK

>AmGr2
MHSEDQIQLMMLKTKDGLGEIPKGKGRGSNLKIWSSVMYHKDDNNIEDISANQENDLSTKRPRAERNYFRN
SEALENFHCAIGPVLKAAQIFGMFPVSGIGSSSLSKLQFKIFSLLTMYSGFIALMISFMTIVSMIHMLKTF
NASTFQIRGGLGAATVGAVFYGNSLVGSILFFSLSSRWVSLQYEWRAMERYIDSNSTEPTRLRWKFFIIST
MVLVLSLIEHVLSIFNNIDGYEWNESNSTFHNFLEIYTLRSHSFIFDTLNYNFVYGLYVFVVSKLATFTWN

FTDLFIMLVATGLAERYKSLNKKLAVTMTKCQAAFNWRELREDYAILSCIVKKVDDHISPIILLSFANNVY
FICLQLLNGLSISDKNSVLSEAYFFGSFAFLICRTCAVTLLTARIHDQSKQSLPYLYNCSTSSYSVEVQRL
QCQLATDDIALTGLRFFSITRNFMLAVAGAIITYEVVLLQFNGK

>NvGr2
MTAEAKPKGHRNASPRRIHFGIGRKMMSFFRSSKSGRGKPRSQHTPIFSKIHPAKRPALHSRRQSSNEDEP
ECFHRAIGNILLMSQFFGILPIRYIRSSSVRNFSFYKFAPRVIYSYFVLLAISVMTSISFLHLFRTLNANS
FQTKGGIADATVGAMFYGNSLLGNLMFLRLCPKWISIQHDWRAMERLIDNNGKWKGPVLRWRFTLISSTIL
SLALLEHILSMVNNTPSDVWFGKKNLEDFLIIYTNKSHRFIVRNVDYNFTLGLFIFFISKVSTFTWNFTDL
FIMLVSTGLAERYKRLNARILEATPAQLSVTDWHELRECYAVLSALVKKVDNEISGIILLSFTNNIYFICL
QLLNGLSPSTAEHPIINSIYFFGSFIFLIGRTTAVTLLTARINDQCKLILPILYNCPSVNYCREAQRLQQQ
IATDDVALTGHRFFSITRNFMLAVAGAIVTYEVVLLQFNIALQRDEELNNMAAGSNG

III. Predicted amino acid sequences of Odorant Binding Protein queries

>BM_BM_OBP28
MLKVFIVTFFAFQLSAIARLQANGCVAVPFPKDKTIIIVEAMKSCIAKTGANPNFIDVIRSGKVSEDEKFK
EFYYCTCNDTGFVNPDGHIKVKECIELFPKETQPLVEPVIKNCDKEEGVNKYDTLFKFLKCFQETSPVRVA
LA

>BM_BM_OBP22
MLKVFVVVVCTLGASQLCAALYTQKVAVSFPKDKTTIVVEAMKSCIAKTGANPNVIEVISSGKVSEDEKFK
EFFYCACNDIGVVNPDGHIKVKECIELFPKETQPLVEPVIKNCDKEGVNKYDTLFKYLKCFQETSPVRVTL
A

>BM_BM_OBP26
MKSVVLICLAFAVFNCGADNVHLAETQKEKAKQYTSECVRESGVSTEAINAAKIGKYSKDKAFKNFVLCFF
NKSAIFNSDGTLNMDVALAKLPPGVNKSEAQSVLKQCKNKTGQGAADKAFEIFRCYYKGTKTHILF

>BM_BM_OBP27
MKSVVLICLAFAVFNCGADNVHLTETQKEKAKQYTSECVKESGVSTEVINAAKTGQYSEDKAFKKFVLCFF
NKSAILNSDGTLNMDVALAKLPPGVNKSEAQSVLEQCKDKTGQDAADKAFEIFQCYYKGTKTHILF

>BM_BM_OBP25
MKSVVLICLAFAVFNCGADNVHLNEDEREKANWYTAECGVETGVSTEVINAAKIGKYSKDKAFKKFVLCFF
KKSAILNSDGTLNMVVALAKLPSGVNKSEAQSVLEQCKNKTGQDAADKAFAILQCFHKGTKTHILF

>BM_BM_OBP23
MTSKVLLSCVVLAVLATTVLAEDSRKLVSFAPEVAKKLKVLIQECLNENGLGEDAIEVIRAGEYREDEPFQ
NLVYCAYKKFGALDENNRIISQVAAASFPKDIDVVTVIESCGKEDGNTPVEQVFKYFKCFQKNSPVRMQLY

>BM_OBP30
MRSFVILLNYGLLCCGQFMAEDYYYDIVTRDPDDLMREKENEVRALRAFQADCAEDVQVKPDLVVNLKSGD
WQTEDVSLKKWALCVLMKLGLMTAQGVFKMNEAMSKIPDMNDKIIAEKLIDDCLSLQATTPHDAAWNYIKC
HHQKDPEGNFSSLNIF

>BM_OBP31
MKTFIVFVVCVVLAQALTDEQKENLKKHRADCLSETKADEQLVNKLKTGDFKTENEPLKKYALCMLIKSQL
MTKDGKFKKDVALAKVPNAEDKLKVEKLIDACLANKGNSPHQTAWNYVKCYHEKDPKHALFL

>BM_OBP29
MTGPAAAAVLLALLAAAGQATTGCKNCVILGKEERAMFRSHSDACLAQSRVEPRLLESMMNGELIDDAALR
KHVYCVLLSCKMIGKDGKLLKAAILGKLAARPAGRDVTKVLEACAEQPGASPEDVAWNIFRCGYNRKAVLF
DYMPAGGASSGNTENHP

>BM_OBP14
MERKDFYLLIVVVALTSGVSSMSRQQLKNSGKMLKKQCMGKNDVTEEEIGDIEKGKFIEQKNVMCYIACIY
QMTQIIKNNKISYEASIKQIDLMYPPELKESAKASAGRCKDVSKKYKDICEASYWTAKCMYEDNPKDFIFA

>BM_OBP17
MTRQQLKNSGKIMKKTCMPKNDVTEEEIGQIEQGKFLEQRNVMCYIACIYTVTQVVKNNKLSYDAVIKQVD
VMFPAEMRPAVKAAAENCKDISKTFKDICEASYWTAKCMYDFDPKNFVFP

>BM_OBP15
MFLKNIFIECVLLYFVMLNTSFVNTMTKQQIKNSGKILKKACISKNDVTEDQISDIDKGKFIEDKNVMCYI
ACVYSMSQVVKNNKFVHDAMVKQVDMMFPTEMRDAVKASIANCRGVAKNYKDICEASFWTAKCMYEFDPAN
FVFA

>BM_OBP16
MRISFLFLISVTIITFDSVFAMTRAQVKKTMTIMKNQCMPKNGVTEDQVGKIEEGIFLENHNVMCYIACVY
KTIQVVKNDRLDKDLISKQIDVLYPQEIRESTKKAVGDCINLQEKYDDWCEGIFRSTKCLYEKDPANFIFP

>BM_OBP18
MILIVIAKFLILISLCETMTMKQIKNTGKMMRKSCQPKNNVDDEKINPINDGVFIEENEVKCYIACIMKMA
NTMKNGKLNFEAAMKQADLLLPDEMKEPTKEAIVACRKVADSYKDVCDASFHVTKCIYNHNPSVFFFP

>BM_OBP21
MITASLHVIFALLAFVYGGKDKPVLSEEIKEIIQTVHDECVGKTGVSEEDITNCESGIFKEDVKLKCYMFC
LLEEAGLVNDDGTVDYEMFTSLIPEEYFDRATKMIFSCKELDTPDKDKCERAFEVHKCSYEKDPDFYFLF

>BM_OBP20
MAVHIFLILASYMALAAHGQLDDEIAELAAMVRENCADESSVDLNLVEKVNAGTDLATITDGKLKCYIKCT
METAGMMSDGVVDVEAVLSLLPDSLKTKNEASLKKCDTQKGSDDCDTAYLTQICWQAANKADYFLI

>BM_OBP12
MTSFMVFFVLSVLTLKYSDALTDEQKNKIQSKFIEIGAECIVEHPISIDDINSFKNKKFPSGVNAGCFVAC
IFNKIGLFDDKGNLSHNSALEKAKGIFNADEEVKNLEEFLNRCAKVNGEAVGDGVKGCERAKLAYNCLIEN
SLEFGFNIDF

>BM_OBP11
MSANSFVVLAFCALAVGVNALTEEQKAEITKSSLPLIAECSKEFSVNQGDIDAAKKLGDPSGLNSCFVGCF
MKKAGIINASGLFDVAATIEKSKKYLTSEEDLKAFEKLTETCAPENDKPVSDSDKGCERAKLLLDCFVANK
GSFSVFSL

>BM_OBP13
MLKIHVLLCFGMAILYFGSAKAVTPEESKAFEAFAKPVIEQCQKDFGMDKESFAQKNLDEIDECLIACVVE
KFGITNDEKIDGDALKALVTKFVGNEEERNKINKIVEECTEDANKSGDGTCNTSTILFLCLLKNGKDLWGF

>BM_OBP8
MLRVVVICVCFLVIAPYGINASSLDDLKMVYKNVIKECVGDYPITAADLKLIKARQIPNDDIKCVFACAYK
KTGMMTEEGMLSVEGIKDMSQKYLSDNPEQLRKSKEFAEACSSVNDQQVSDGTKGCERAALIFKCSTEKIT
NFGFEL

>BM_OBP9
MLRVVVICVCFLVVAPYGINAVSYEQKIKIRDQLDRAGFECFKDHKITEDDIKNLRANKPATGENVPCFIA
CVMKKTGVMNDQGVIRKGPVLELAKKVLADDKDIKKLQDYIHSCSHVNSETVHDKGKGCEFAMQAYTCMSA
NASKFGFNI

>BM_OBP10
MLRVVVICVCFLVIAPYGINAVSDEQKIKIREQIDKSGFECFKDHKITEDDIKNLRARKPATGENVPCFIA
CVMKKTGVMNDQGVIHTEPVLQLAKKVLTDDKDIKKLQDYIHSCSHVNSKTVHDKGQGCEFAIQTYTCMSA
NASKFGFDV

>BM_OBP7
AVTEEELKIEFTKLVMKCTKDHPVDMSELMQLQQLIAPKKTESKCLLACAYKLNGVMTSQGLYNLEHAYKI
AEMSKNGDEKRLENGKKVADICVKVNDVEVSDGEKGCERAALIFKCTLENAPKVFKFGSSEYNCQ

>BM_OBP19
MTSAKTDVEIKAWFLGQAVECSKDHPVTTEELRMHKHELPDSKNAKCLMKCVFRKCNWLDSKGMYDINAAY
ASSTKDFSDDKTKQENANKLFDTCKSVNEENVGDGEEGCDRSLLLAKCLTKAAPQVSIYYS

>BM_OBP1
MWKLVVVLTVNLLQGALTDVYVMKDVTLGFGQALEQCREESQLTEEKMEEFFHFWNDDFKFEHRELGCAIQ
CMSRHFNLLTDSSRMHHENTDKFIKSFPNGEILSQKMIDMIHTCEKKFDSEPDHCWRILRVAECFKDACNK
SGLAPSMELILAEFIMESEADK

>BM_OBP2

MFSFLILVFVASVADSVIGTAEVMSHVTAHFGKTLEECREESGLSVDILDEFKHFWSDDFDVVHRELGCAI
ICMSNKFSLMDDDVRMHHVNMDEYIKGFPNGQVLAEKMVKLIHNCEKQFDTETDDCTRVVKVAACFKKDSR
KEGIAPEVAMIEAVIEKY

>BM_OBP3
MSIQGQIALALMVYMAVGSVDASQEVMKNLSLNFGKALDECKKEMTLTDAINEDFYNFWKEGYEIKNRETG
CAIMCLSTKLNMLDPEGNLHHGNAMEFAKKHGADETMAQQLIDIVHGCEKSTPANDDKCIWTLGVATCFKA
EIHKLNWAPSMDVAVGEILAEV

>BM_OBP4
MKLQVVLVVLTVEMVCGSRDVMTNLSIQFAKPLEACKKEMGLTETVLKDFYNFWIEDYEFTDRNTGCAILC
MSKKLELMDGDYNLHHGKAHEFARKHGADETMAKQLVDLIHGCSQSVATMPDECERTLKVAKCFIAEIHKL
KWAPDVELLMAEVLNEVSWKS

>BM_OBP6
MARYNIVVAVLVLGVVGARGSSEAMRHIATGFIRVLDECKQELGLTDHILTDMYHFWKLDYSMMTRETGCA
IICMSKKLDLIDGDGKLHHGNAQAYALKHGAATEVAAKLVEVIHGCEKLHESIDDQCSRVLEVAKCFRTGV
HELHWAPKLDVIVGEVMTEI

>BM_OBP5
MKQRLRVLLLRFCILQTVLSESGVDVVKNLSLSFARFFLECDEERHFQPEVRLKVMTFWYSESSTWDRDVG
CAFLCIFKKMEIDNPQDPSYRTHLELLSFANSEDNKIANQMVEIFYACGENTETDPCLWALEQVKCYKNRI
NQLGLTPTF

>BM_OBP32
MYSHKYLNDFTNIPEILIIILLSSVALMSYGYNTKLFSHSLGSEPSLSILYARDKKSDKVTNECLMEMYPKN
LYKYPLRIDRNDIPCIIHCVLKKFGIISNDGFINIKNYYRRVQAIHRYDPRILISDVGETCAQNINGMNLD
HDVCKKAKVFNDCTQLYAISYREPEDW

>BM_OBP33
MYAHDKLSDMIADQCLNEMYPRSKRLEIEESDEPCIIFCVLKKFGIMSPTGVINLEAYRKRVQLPEQLAQR
NSINDFGSACLESAEATQHKQDVCKKAKVFNECTHLYKILLK

>BM_OBP34
MEKMILLNVFAVVLPCVLASRTRGSSGTLVDFTDPKVQGHLDALVRMAQSCVIKVRATPKDVRAYFTNSSP
VSRSGQCFATCMLEQSDIINHGKVNRDLLVHLAGLVNGKNSRVVRKLNSVSRLCLDSISGMTDRCQLASTY
NDCLNENMIEFAFPLDIAEEAVRKMPFHLIQPK

>BM_OBP35
GMSTHVLDFKRNMTECLKEVQNNDKRPIKRLSPKQESPIHGECLIACVLKKNGVIQNGKVNKDNLMALVSK
FHAKETKLMKKLEKNLDRCINISVKNHDECSLASQLNDCTNDIMASSKQKILFNY

>BM_OBP39
MVRKISALLCCFCVLGISMCDSAISTDNEQRCKNPPTAPQKIERVITLCQDEIKLSILREALDVIKEEHTM
PAERKRNKREVPFTHDEKRIAGCLLQCVYRKVKAVDGFGFPTLEGLVGLYSDGVNERGYFMAVLEASRECL
MKNHDKFSRTTPMDNGRNCDVSFDIFECISDRIGEYCGTSGL

>BM_OBP38
MANLVLLLTFVLMTLSMARLKSTEAPKSKTALFNDQDNMGYEELDMEEIMSACNESFRIEYAYLESLNDSG
SFPDETDKTPKCYIRCVLEKTEILSENGVLNPATAALVFAGERNGKPMSDLEEMAVACADRHEKCKCEKAY
NFVKCLMYMEIDKYEKKN

>BM_OBP36
MAVSEISRILTFLTIVSFIYIVYSFKPLTKDEHIERYNKMNEDIEPFRKNLTECARQVKASMADVEKFLKR
IPQSNMEGKCFVACILKRNSLIKNNKLSQENLLEVNRAVYGDDSEVMSRLKTAILECSKIVEDIFEICEYA
SVFNDCMHMKMEHILDKITMERRMEALGQMSSNPDEWSEEEDEMLKLVKDEL

>BM_OBP37

MFYPFRFTLLFYGLFVIYLVRAEPEKENHFTLALKKTLFSTARSCMSHVNANETDLEYLRKDPPFPDKAAC
IIKCLLEKIGVVKNNKYSKMGFLTAVSPLVFTNKKKLDHYKSVSENCEKEINHDQTTECELGNEVVSCIFK
YAPELHFKT

>BM_OBP40
MSEFIQPSWRTQCNFRLNWDNRNRLSIDISHGAATTQTPVPTTKPKALRDFMVVPQSCDKTTCVFKKLNIV
SDKGVVDVKSFIKLLDKFTNSYPVWNSAKARVITTCLRKSLIAYDGGCELNNILACTFDVLSENCPLNGNN
QTC

>BM_OBP41
MLTILFLLPIVVGVLSGNIPEQPRVYCGELPNTIYSCLGNPKIIQPEVSEKCNKPISECDKTRCIFKESGW
AKNNVIDKKKVSDYFEQFAKDNPDWSAAVQNFKTTCLSDSLKPQGVDTNCPAYDIIHCALISFIKFASPSQ
WSTSEQCVYPRQYAGACPVCPERCFAPSVPNGSCNACLALLRTP

>BM_OBP42
MMGYACVFVILAVLQAISAEDPPGLPPFLKDAPEKCKSPPRVKNPNECCISEPFFKEADFIECGIEKPGSE
RGPPDCSKQNCLLKKYNLLKNDETPDIEAIKSLLDKYIEKNPSFKSSVEKAKECLREDLPGPPQICLANRM
TLCIGTVLLMECPDEKWNTTDDCKAFKDHMTECQKYFPK

>BM_OBP43
MKVCVLFAIFTVAQAAKATLKPISACCNIPELGNPEPLAECSNPKLPGPCKDIQCVFEKSGFLTENKTLIK
EAYKTHLRQWAKEHEGWSVAVEKAISDCVDKDLRQYLEFPCSAYDVFTCTGIAMLKKCPNEHWTC

>BM_OBP44
MSRLVLFFTILVVLQEFIINLYFNFITEIDSCCVKKYPKLFDSEFITECYNTQRKANDKCERDMCVARKLN
LLTEEDSINKDALLRFVEEGFKTEIDLVNAIKKKCFEEDISNIGKPEMCEVAKYKICITSRMAEDCPKWDS
KGICSSAQQKVENFMKMLS

IV. Predicted amino acid sequences for Chemosensory Binding Protein queries

>bmo-csp1
MKVLIVLSCVLVAVLADDKYTDKYDKINLQEILENKRLLESYMDCVLGKGKCTPEGKELK
DHLQEALETGCEKCTEAQEKGAETSIDYLIKNELEIWKELTAHFDPDGKWRKKYEDRAKA
KGIVIPE

>bmo-csp2
MKSSLFCVLVLTVVVSSSRQQSYPRNDNININAILQNDRILLGYFKCVMDRGPCTKDGKT
FKRALPEALPTACARCSNKQKAAFRTLLLAIRARSEPSFLELLDKYDPSRSNRELL

>bmo-csp3
MACVAVTWARPESTYTDKWDNINVDEILESNRLLKGYVDCLLGKGRCTPDGKALKETLPD
ALEHECVKCTGKQKSGADKVIRHLVNKRPDLWKELAVKYDPDNIYQARYKDKIDAVKGSA

>bmo-csp4
MKTVIVCLLALTAVALARPEQYTDKYDTVDLDQLISNRRLLIPYVHCILEKGQCTAEGKE
LKSHIKEALETNCAKCTKAQKGGTEKMIGHLINHEAEFWEELKAKYDPTNEFTKKYETEL
KRVTA

>bmo-csp5
MNSLIAFCLFAVLAVALARPDDKYTDRYDNVNLDEVLSNSRLLKPYIKCILDKDRCAPDA
KELKEHIREALETECAKCTEAQKKGTRRVIGHLINNESKSWNELTAKYDPENKFTAKYEK
ELREIKA

>bmo-csp6
MKCLTIAALLFVAGLSIAEKYTDKYDNIDVDEILENRKLLVPYIKCVLDEGRCTPDGKEL
KAHIKDGMQTACAKCTDKQKVSARKIVKHIKQHEADYWEQMKAKYDPKDEFKEIYEGFLA
GQN

>bmo-csp7
MKSLIVLSCLLAACLAADLSKYENFDVEPIVTSDRLLKAYINCFLDKGRCTPEASDFKKA
LPDTIATNCGKCTEKQKANVRKVIKVIQQKHSTEWEKLVKKHDPSGKHRADFDKFLLGS

>bmo-csp8
MRAVIFLYTCVFVVVGQDINAMMSMPKYDERYDYLDVDDIFRNKRLVRNYVDCLINAQRC
TPEGKALKRILPEALRTKCIRCTERQKRTSVKVIRRLKNEYPEEWAKLASRWDPTGDFTR
YFEDYLAKEHFNTIPGSGPTVNVLSLQTTPPPPPPPPSRPASVFTNPPPPVMSTSPRPVV
LNRFRR

>Bombyx mori CSP9 mRNA for chemosensory protein9, complete cds
MKFVLALIALAVVVAARPNDDLFYDKKYDNFNVDEIIDNPRLLKAYTFCFNDKGKCTAEG
NDFKKWIPESLQTSCGKCSEKQKYLVAKFVHAIKDKMPDEFDILRKLHDPKGEYTENLDK
FLETYGH

>bmo-csp10
MKSSLFCVLVLTVVVSSSRQQSYPRNDNININAILQNDRILLGYFKCVMDRGPCTKDGKT
FKRALSEALPTACARCSNKQKAAFRTLLLAIRARSEPSFLELLDKYDPSRSNRELLYTFL
ATGL

>bmo-csp11
MKGFYVLCFALFAAVYCKETYSSENDDLDIEALVGNIDSLKAFIGCFLETSPCDAVSGDF
KKDIPEAVAEACGKCTPAQKHLFKRFLEVVKDKLPQEYEAFKTKYDPQGKHFDALLSAVA
NS

>bmo-csp12
MFMLFIISFIIVPVLKCCGTETSTYTTQYDEVDIKEIMGNERLLVAYIGCLLDKNPCTPE
GKELKRNIPDALQSDCSKCSDKQRENADAWIEFMIDNRPEDWTKLEER

>bmo-csp13
MKLLLVFLGLFLAVLAQDKYEPIDDSFDASEVLSNERLLKSYTKCLLNQGPCTAELKKIK
DKIPEALETHCAKCTDKQKQMAKQLAQGIKKTHPELWDEFITFYDPQGKYQTSFKDFLES

>bmo-csp14
MKSSLFCVLVLTVVVSSSRQQSYPRNDNININAILQNDRILLGYFKCVMDRGPCTKDGKT
FKRALPEALPTACARCSNKQKAAFRTLLLAIRARSEPSFLELLDKYDPSRSNRELLYTFL
ATGL

>bmo-csp15
MIENFYSKCTISKSVLFLCLIFLPYALNQKYYDSRYDYYDIDHLVQNPRLLKKYLDCFLG
KGPCTPIGRLFKQVMPEVITTACAKCTPTQKRFARKTFNAFRRYFPETLMELRRKFDPES
KYYDAFEKVITNA

>bmo-csp16
MIEWKRFKILHFLSYLGLLVLVVVCAAQQNRPQVTDTALDEALNDKRFIQRQLKCALGEA
PCDPIGKRLKTLAPLVLRGACPQCSPQETKQIQKTLSYVQRNFPQHWAKLVRQYAG
